# De Novo Origin of Human Protein-Coding Genes

**DOI:** 10.1371/journal.pgen.1002379

**Published:** 2011-11-10

**Authors:** Dong-Dong Wu, David M. Irwin, Ya-Ping Zhang

**Affiliations:** 1 State Key Laboratory of Genetic Resources and Evolution, Kunming Institute of Zoology, Chinese Academy of Sciences, Kunming, China; 2 Department of Laboratory Medicine and Pathobiology, University of Toronto, Toronto, Canada; 3 Banting and Best Diabetes Centre, University of Toronto, Toronto, Canada; 4 Laboratory for Conservation and Utilization of Bio-resource, Yunnan University, Kunming, China; University of California Davis, United States of America

## Abstract

The de novo origin of a new protein-coding gene from non-coding DNA is considered to be a very rare occurrence in genomes. Here we identify 60 new protein-coding genes that originated de novo on the human lineage since divergence from the chimpanzee. The functionality of these genes is supported by both transcriptional and proteomic evidence. RNA–seq data indicate that these genes have their highest expression levels in the cerebral cortex and testes, which might suggest that these genes contribute to phenotypic traits that are unique to humans, such as improved cognitive ability. Our results are inconsistent with the traditional view that the de novo origin of new genes is very rare, thus there should be greater appreciation of the importance of the de novo origination of genes.

## Author Summary

The origin of genes can involve mechanisms such as gene duplication, exon shuffling, retroposition, mobile elements, lateral gene transfer, gene fusion/fission, and de novo origination. However, de novo origin, which means genes originate from a non-coding DNA region, is considered to be a very rare occurrence. Here we identify 60 new protein-coding genes that originated de novo on the human lineage since divergence from the chimpanzee, supported by both transcriptional and proteomic evidence. It is inconsistent with the traditional view that the de novo origin of new genes is rare. RNA–seq data indicate that these de novo originated genes have their highest expression in the cerebral cortex and testes, suggesting these genes may contribute to phenotypic traits that are unique to humans, such as development of cognitive ability. Therefore, the importance of de novo origination needs greater appreciation.

## Introduction

The origin of new genes has always been an intriguing evolutionary question [1]. New genes play significant roles in the evolution of lineage specific phenotypes and adaptive innovation [2]. The origin of genes can involve gene duplication, exon shuffling, retroposition, mobile elements, lateral gene transfer, gene fusion/fission, and de novo origination [1]. The mechanisms for many of these processes have been extensively studied; however, studies focused on de novo origination are few, and it is commonly considered to be a very rare process [3], [4].

In 1970, Susumu Ohno proposed that new genes arise from existing genes, and that the de novo gene origination of a gene from a random sequence would be highly unlikely [3]. Francois Jacob even claimed that “the probability that a functional protein would appear de novo by random association of amino acid is practically zero” in a paper he published in 1976 [4]. Today, we know that this evolutionary process is not impossible. For the de novo origin of a protein-coding gene two steps are needed [2], [5]: (1), the DNA must be transcriptionally active, and (2) it must evolve a translatable open reading frame; however, these two steps can occur in either order. Pioneering research in 2006 clearly showed that new genes could originate from non-coding sequences in *Drosophila*. Levine et al. identified five novel genes in *Drosophila melanogaster* that were derived from non-coding DNA [6]. These *Drosophila* genes were found to be expressed predominantly in the testes, and four of them were X-linked [6]. Similarly, Begun et al. found that the *Acp* genes, which code for small proteins in *Drosophila*, originated from noncoding DNA [7]. Over the next few years, there were several additional reports of the characterization of de novo-originated *Drosophila* genes [8]–[10]. In particular, Zhou et al. (2008) identified nine genes that originated de novo through a systematic search strategy, and proposed that the de novo origin of genes plays an important role in the origination of new genes, and estimated that about 11.9% of the new genes that originated in the *Drosophila* lineage had arisen de novo [10], however, it is unclear whether all of these new *Drosophila* genes encode proteins. In 2009, Knowles and McLysaght identified three putative protein coding genes: *CLLU1*, *c22orf45*, and *DNAH10OS*, which had a de novo origin in the human genome. These genes were identified by employing a straightforward, but rigorous, procedure which provided transcriptional and translational evidence, and allowed them to estimate that about 0.075% of the human protein coding genes may have originated de novo from noncoding regions [5]. Li et al. (2010) described another de novo protein-coding gene: *C20orf203*, which is associated with brain function in humans [11]. Additional searches for de novo genes have resulted in the identification of two protein coding genes by Cai et al. [12] and Li et al. [13] in the *Saccharomyces cerevisiae* genome, a gene by Heinen et al. in *Mus musculus* that arose de novo within the past ∼2.5–3.5 million years in a large intergenic region [13], a gene in rice [14], at least 13 protein-coding genes by Yang and Huang in the *Plasmodium vivax* genome [15], and a *Drosophila* gene, *Noble*, in a recent study by Gontijo et al. (2011) [16]. Despite all of these studies, the de novo origin of new protein-coding genes from non-coding DNA region in the genome is still considered to be a very rare event.

The advent of large-scale genome sequencing has resulted in the bioinformatic prediction of many lineage-specific genes in genomes, suggesting that there may be a significant rate of de novo origin for genes. A large proportion of these genes, however, are likely falsely predicted genes [17], [18] and the true numbers of functional de novo originated genes remains unclear. While gene duplication certainly plays a role in the origin of new genes [3], we hypothesized that the rate of de novo gene origination is not extremely low and also plays an important role in the origin of new genes. Here by comparing genomes among primate species we identified 60 de novo-originated protein-coding genes in the human lineage, including 27 genes identified based only on genes found in Ensembl version 56, and 33 genes identified based on the genes that were now excluded in version 56 of Ensembl, but were present in versions 40–55 of the human genome. Each of these new genes has both transcriptional and proteomic evidences supporting their functionality. The number of de novo genes that we found in the human genomes is much higher than that expected based on previous estimates of the rate of de novo origination, therefore, we suggest that a greater appreciation of de novo origination of genes is needed.

## Results

### Search for De Novo-Originated Genes in the Human Lineage

**Figure 1:**
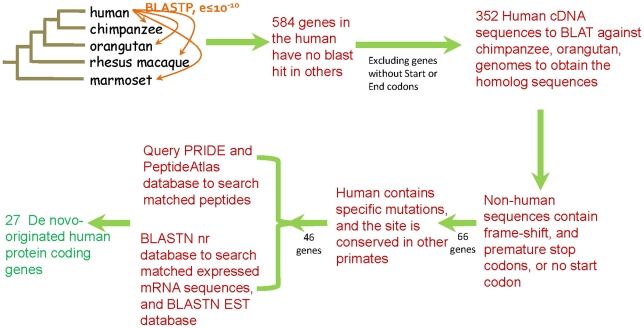
Pipeline for the identification of de novo originated protein-coding genes in the human genome. BLASTP searches of human protein sequences against proteins of other primates identified 548 human genes without protein orthologs. After excluding these genes having no start or stop codons, 352 human coding sequences were used in BLAT searches to find orthologous genes in chimpanzee and orangutan and these sequences were examined to confirm the presence of disrupting mutations. 66 genes with disrupted open reading frames in chimpanzee and orangutan were examined to identify those with human-specific mutations that generate intact open reading frames, resulting 46 candidates. These genes were used as queries of mRNA and proteomic databases to confirm transcription and translation. The pipeline yielded 27 de novo originated protein-coding genes.

We performed a simple, conservative, but systematic pipeline to search for genes that originated de novo in the human genome since divergence from the chimpanzee (Figure 1). All human protein sequences were searched using BLASTP against the protein databases of other primates, i.e. chimpanzee, orangutan, rhesus macaque, and marmoset, with orthologs identified using an E-value threshold of 10^−10^. After the BLAST procedure and excluding proteins shorter than 100 amino acids and short protein sequences from alternatively spliced genes, we retrieved 584 genes from the human genome that did not have a hit in other primates. Human sequences that did not have a start (i.e., ATG) or stop codons were excluded and the remaining 352 genes were searched using BLAT against the chimpanzee and orangutan genomes in the UCSC database (http://genome.ucsc.edu/, [19]) to identify orthologous sequences. In addition to the bioinformatic analyses all of the sequences underwent extensive manual checks. Human genes for which an orthologous gene region (i.e., highly similar sequences) could not be identified in the chimpanzee or orangutan were discarded. Genes that had many duplicates in the human genome were also discarded. To be a candidate de novo originated gene, in addition to having a potentially translatable open reading frame in the human genome, the gene must have been present, and disrupted (i.e., non-translatable), in both the chimpanzee and orangutan genomes, e.g., the chimpanzee and orangutan sequences must lack an ATG start codon or have frameshift-inducing indels or nucleotide differences that result in a premature stop codon. Chimpanzee and orangutan sequences lacking only an ATG start codons were searched to determine whether they had alternative start codons, either upstream or downstream of the human ATG that could generate frame complete translatable open reading frames. Chimpanzee or orangutan genes that possessed premature stop codons but retained predicted protein lengths longer than 80% of the human proteins were discarded for analysis, while those with predicted proteins that were shorter than 80% of the size of the human proteins were kept for the analysis of human de novo genes (see Dataset S1). To exclude the possibility that the new gene had been generated in the primate ancestor and then lost in parallel in both the chimpanzee and orangutan lineages we searched for human specific mutations that were responsible for generating the completed protein-coding open reading frame. Only those genes that had a human specific mutation that generates an open reading frame and where both the chimpanzee and orangutan retained the ancestral state at these positions, thus disrupting the open-reading frame, were kept (see Dataset S2). These stringent criteria yielded a set of 46 genes. Lastly, the coding sequences of these 46 putative de novo human genes were used as queries in searches of databases for evidence of expression at the mRNA and protein level. Expression at the mRNA level was assessed by BLASTN searches of the NCBI (http://www.ncbi.nlm.nih.gov/) nr (non-redundant) database, to search the corresponding matched expressed mRNA sequence, and the UCSC (http://genome.ucsc.edu/) EST database, to search for short expressed sequence tags. Evidence for the existence of the protein was obtained through searches of two proteomic databases, PRIDE [20] and PeptideAtlas [21] (Dataset S3). The PRIDE and PeptideAtlas databases are composed of peptide sequences derived from proteomic experiments. Searches of these databases resulted in the identification of 27 novel human genes that have matching expressed mRNA sequences in the GenBank or UCSC databases, thus must be transcribed, and also have evidence for being translated as they have matching peptides from the proteomic databases (Table S1). The mRNA evidence suggests that none of these human genes have splice variants.

Intriguingly, *CLLU1*, *c22orf45*, and *DNAH10OS*, three human genes identified as having a de novo-origin by Knowles and McLysaght [5] were not found by our search. Knowles and McLysaght [5] had used protein data from version 46 of Ensembl for their study while we use sequence data from version 56. *c22orf45* and *DNAH10OS* were no longer annotated as genes in version 56 of Ensembl, however *CLLU1* still was. The peptide, PAp00140670 (HIIYSTFLSK), that supported the translation of *CLLU1*, though, is no longer present in the current build of PeptideAtlas [21], yet the peptides that support the translation of *c22orf45* and *DNAH10OS* still remain in the proteomic database. Thus the absence of a supporting peptide, for *CLLU1*, and the absence of annotated genes, for *c22orf45* and *DNAH10OS*, prevented our approach from identifying these three previously identified genes as having a de novo origin. Given the differences in protein content between versions 46 and 56 of Ensembl, we therefore identified protein sequences that had been present in previous versions of the human genome (Ensembl versions 40–55) but were no longer annotated as gene products in version 56. These human protein sequences were then used in BLASTP searches against other primate protein databases, adopting the same pipeline that we described above, resulting in the identification of an additional 33 de novo-originated protein coding genes that are supported by human expression and proteomic data (Figure S1, Table S2, Dataset S1, Dataset S4 and Dataset S5). Of the three de novo genes, *CLLU1*, *c22orf45*, and *DNAH10OS*, identified by Knowles and McLysaght [5], only *DNAH10OS* (ENSG00000204626) was identified in our study. As described above, peptide PAp00140670 (HIIYSTFLSK) that supported the translation of *CLLU1* is no longer present in the current build of PeptideAtlas, thus does not meet our criteria of a de novo gene with transcription and translation evidence. The orangutan genome predicts a gene sequence orthologous to *c22orf45* that has a complete translatable open reading frame, suggesting that it has a much earlier origin. It is important to note that the sequences of all of our 60 predicted de novo genes, 27 from the original screen and 33 from our subsequent screen are present in the most current version of the human genome (GRCh37/hg19), thus all 60 genes were kept for our subsequent analyses.

We identified a total of 60 protein-coding genes that originated de novo on the human lineage since divergence from chimpanzee. Each of these new genes is found as a single copy coding gene, with no other highly similar coding sequence in the human genome, indicating that they were not generated by gene duplication in the human genome. In addition, the orthologous sequences in the chimpanzee and orangutan genomes are found as single copies (except ENSG00000230294 which has two orthologous copies in the orangutan, but both of these sequences are disrupted, see Dataset S2 for sequence alignment). Pairwise divergences between the sequences were consistent with the accepted one-to-one orthologous relationships between human, chimpanzee, and orangutan. All of the de novo genes were found to be composed of a single exon, with the exception of ENSG00000204292, which has two. Only one of the genes is located on the X-chromosome; the remainders appear to be distributed randomly to the autosomes.

To determine whether these new genes are fixed in human population, we searched the human population polymorphism data in HapMap (Phases I, II, and III, http://hapmap.ncbi.nlm.nih.gov/). There was no evidence for deletion or insertion of any of the genes from the HapMap data. Only one of the genes, ENSG00000206028, was found to have a SNP causing a premature translation stop. This observation suggests that ENSG00000206028 has not been fixed in the human population.

Our finding of 60 de novo genes, 59 of which are fixed in the human population, suggests that the de novo origin of protein coding genes on the human lineage is not a rare event. Since the chimpanzees and humans shared a common ancestor ∼5–6 million years ago, this indicates that the rate of origin of de novo genes is ∼9.83–11.8 genes per million years, an estimate that is much higher than previously reported [5], [10], [22].

### Expression Analysis by RNA–Seq

To gain insight into the potential functions of these de novo originated genes we examined the expression of these genes using RNA-seq data. RNA-Seq is a recently developed approach for transcriptome profiling using high-throughput sequencing technologies, and is powerful for detecting the expression of genes [23]. Here, we examined the expression of the de novo originated genes using previously described RNA-seq align data [22], [23] from 11 human tissues: adipose, whole brain, cerebral cortex, breast, colon, heart, liver, lymph node, skeletal muscle, lung and testes. Since the exact transcripts for the de novo genes had not been defined, we defined the expression level of these genes as the numbers of unique RNA-seq reads that map to the coding region divided by the length of the coding region, instead of typically used number of reads mapping to a transcript divided by transcript length.

**Figure 2:**
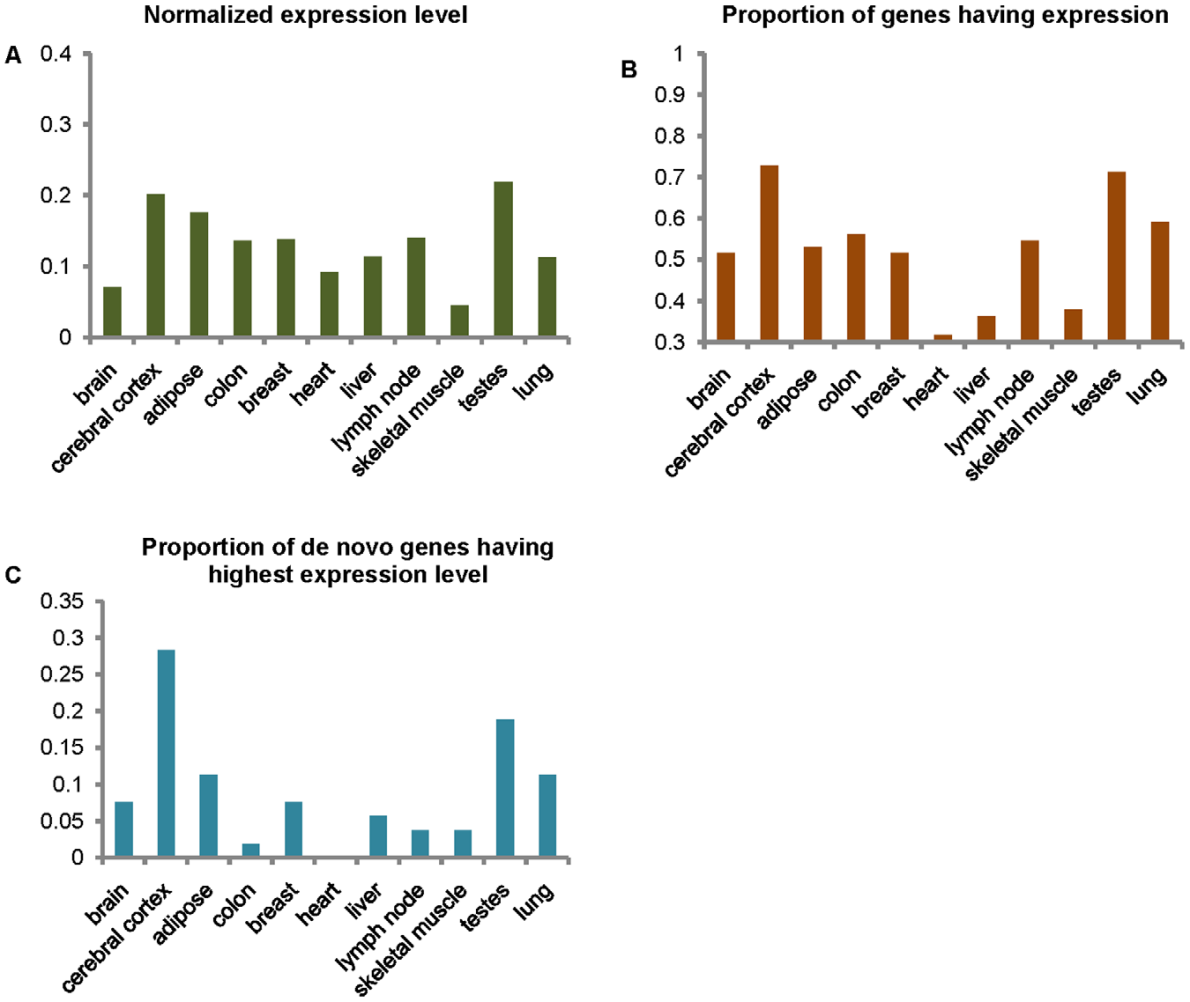
Levels of expression of de novo genes in 11 tissues. (A) Mean normalized expression levels of de novo originated genes in 11 tissues are defined by the mean level of expression as the numbers of unique reads mapping to coding regions divided by the total length of all the coding regions, divided by the total number of valid reads in the samples (×10^−8^). The vertical axis represents value of mean the normalized expression levels and abscissa axis represents the 11 tissues. (B) The proportion of the de novo originated genes that have expressed reads in the 11 tissues. The vertical axis represents the values of proportion, and abscissa axis represents the 11 tissues. (C) The proportion of the de novo originated genes having their highest normalized expression levels in each of the 11 tissues. The vertical axis represents the values of proportion, and abscissa axis represents the 11 tissues.

Evidence for expression, i.e., the mapping of reads, was found in the RNA-seq data for 53 of the 60 genes. Expression data for the 7 genes not represented in RNA-seq data had been found from other sources (e.g., EST data) in the NCBI database. Of these seven genes, three had evidence of expression in tissues other than the 11 tissues represented by the RNA-seq data, and four had evidence for expression in the brain, testis or lung. The failure to find evidence for expression of these four genes with RNA-seq data, despite evidence from the NCBI data, may suggest that these genes are expressed are a very low level in these tissues, or the site of expression of the NCBI data may be incorrect (e.g., due to contamination by other tissue). Typically, the expression levels of the de novo originated genes are very low. The mean level of gene expression, as defined by the number of reads mapping to these genes divided by the total length of their coding sequences, is highest in the testes, and second highest in the cerebral cortex (Figure S2). After normalizing for the numbers of valid reads, highest expression was still found in the testes, and the second in the cerebral cortex (Figure 2A). Interestingly, the tissue that had the largest proportion of the de novo genes expressed was the cerebral cortex, with the second being the testes (Figure 2B). Normalized expression levels of the 53 genes with RNA-seq expression data for the 11 tissues were sorted from highest to lowest. The proportion of genes having highest expression level in the tissue, which was defined as the numbers of genes having highest expression level in the tissue divided by total gene number (i.e. 53), was highest in cerebral cortex followed by the testes among these 11 tissues (Figure 2C); however, a similar pattern was not observed for the proportion of genes having second, third, or fourth highest levels of expression (Figure S3). In addition, we also obtain these patterns of the genome wide genes, and normalized these values of de novo genes by dividing the values of genome wide genes. In consistent, the level of gene expression, normalized expression level and the proportion of genes having expression evidences are still highest in the cerebral cortex and testes, except the proportion of genes having highest expression level (Figure S4).

Several genes were found to have intriguing expression patterns (Figure S5). For example, gene ENSG00000187488 is highly expressed in the testes and thus we speculate that this gene may have a role in reproduction. ENSG00000206028 is highly, and specifically, expressed in the cerebral cortex, suggesting that this gene may contribute to the development of the human brain and associated cognitive abilities.

### Evolutionary Rate of the New Genes on the Human Lineage

**Figure 3:**
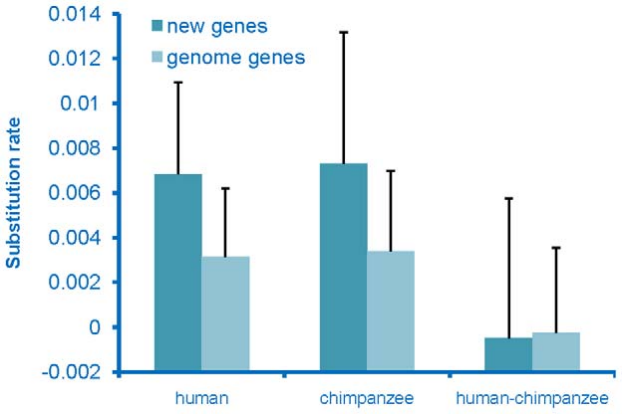
Rate of evolution of de novo originated genes. Rate of de novo originated genes on the human lineage and orthologous sequences on the chimpanzee lineage are compared to the average genome wide genes. Lineage specific substitution rates (+standard deviation) of the de novo genes and genome-wide genes are presented.

To determine whether the de novo genes had come under selective constraints, which would indicate that they had acquired a function, we examined the rate of sequence evolution of these genes. Substitution rates for these sequences were calculated for both the human and chimpanzee lineages and these rates were compared to the genome-wide average rate for genes. The substitution rate for de novo genes was found to be higher than the genome-wide average rate on both the human and chimpanzee lineages (Figure 3), with the chimpanzee sequences evolving at the highest rate. The chimpanzee sequences were expected to evolve at a high rate, as these sequences should act as non-coding sequences rather than genes. The human sequences also evolved at an elevated rate, but at a rate that was slightly lower than that seen on the chimpanzee lineage. This observation is not an unexpected result if these had become functional genes as these new genes originated very recently from non-coding regions on the human lineage, and thus should have been under selection for only part of the time since divergence from chimpanzee, and thus should have a rate higher than the genome-wide average, but lower than the chimpanzee lineage. In addition, young genes have been found to tend to be the subject of weaker purifying selection [24], thus should have higher substitution rates.

## Discussion

Here, we discovered 60 genes that originated de novo on the human lineage, with 59 of them being fixed in the human population. This number of genes implies a rate of de novo generation of ∼9.83–11.8 genes per million years, a rate much higher than previously proposed rates [5], [10], [22]. Despite this high rate, when the rate is expressed in terms of per gene, ∼0.00033–0.00039 per gene per million years, it is still a lower rate than the rate of new gene origin by gene duplication [25], [26]. Our estimated rate, though, for de novo origin may be underestimated due to the conservativeness of our pipeline. First, as described above, in our pipeline, translatable open reading frames must have been complete in the human genome and disrupted in both the chimpanzee and orangutan genomes to be candidates as a de novo gene. Genes that did not have a clear ortholog (i.e., a sequence with very high similarity) in either the chimpanzee or the orangutan genomes (both of which are less complete than the human genome, and thus could be a missing genes) were not used. It is also often difficult to determine whether a protein-coding gene originated specifically on the human lineage or if it originated in a primate ancestor but was then lost on both the chimpanzee and orangutan lineages. The conservativeness of our pipeline thus only allowed us to accept genes where we could clearly show human specific mutations generated complete protein-coding reading frames, and that these were conserved for disrupting state in both the chimpanzee and orangutan genomes. As both the chimpanzee and orangutan sequences should be non-functional sequences, and thus not under selection, there is a reasonable likelihood that a second mutation, in addition to the human open reading frame completing mutation, could have occurred in the chimpanzee or orangutan that would prevent us for identifying these genes as having a de novo origin on the human lineage. A total of 69 genes (20 shown in Figure 1 and 49 in Figure S1. were excluded from our analysis as the ancestral state of the human specific mutation was not conserved in chimpanzee and orangutan. Second, we used only two proteomics databases: PRIDE [20] and PeptideAtlas [21] to show that these genes were translated; however, proteomic data is still limited in terms of tissues and developmental stages sampled and evidence for the protein products of some genes is likely lacking from the current versions of these databases. Here, 56 genes having human specific mutations but no supported peptide evidence were excluded. More diverse proteomic datasets may demonstrate that additional de novo originate genes are indeed protein coding.

RNA-seq expression data suggest potential functions for some of the de novo originated protein-coding genes. De novo genes show higher expression in the cerebral cortex relative to other examined tissues. The brain is responsible for cognitive abilities that occur primarily in the cerebral cortex which is the furrowed gray matter covering the cerebral hemispheres [27]. The cerebral cortex plays key roles in learning, memory, language, thought, emotion, perceptual awareness, and consciousness [27]. Great efforts have been made to explore the origin and evolution of human cognitive ability [28], including examining the contributions of positive natural selection on brain development genes [29] and changes in the expression [30]–[32] and alternative splicing of genes expressed in the brain [33]. Our results provide new information for the field and suggest that de novo originated genes may also be responsible for some of these characters.

Many new genes, generated by diverse mechanisms including gene duplication, chimeric origin, retrotransposition, and de novo origin, are specifically expressed or function in the testes [6], [34]–[38] (reviewed in [2]). Henrik Kaessmann hypothesized that the testis is a catalyst and crucible for the birth of new genes in animals [2]. First, the testes is the most rapidly evolving organ due in part to its roles in sperm competition, sexual conflict, and reproductive isolation [2]. Second, Henrik Kaessmann speculated that the chromatin state in spermatocytes and spermatids should facilitate the initial transcription of newly arisen genes [2]. The reason for this is that there is widespread demethylation of CpG enriched promoter sequences and the presence of modified histones in spermatocytes and spermatids [39], causing an elevation of the levels of components of the transcriptional machinery, permitting promiscuous transcription of nonfunctional sequences, including de novo originated genes.

While this study has resulted in the identification of 60 novel human genes, and emphasized the underappreciated role of de novo origin of genes, there are several important caveats to our study. First, the protein evidence is based on only two proteomic databases: PRIDE [20], and PeptideAtlas [21], both of which have many limitations. For example, the sampling of proteomic databases are still limited to a small number of tissues and developmental stages, and problems with sample contamination still need to be resolved [40]. As larger and better proteomic databases become available the evidence in support of the translation of these novel genes will be strengthened. Second, many of these new genes are expressed at very low levels in the 11 tissues that had available RNA-seq data. These results indicate that many of these genes may play only weak biological roles, or that their functions are not well established.

## Materials and Methods

### Identification of De Novo-Originated Genes in the Human Lineage

Human protein sequences from Ensembl version 56 were used as queries for BLASTP [21] searches against the proteins of chimpanzee, orangutan, rhesus macaque, and marmoset with significant hits being those with an E-value lower than 10^−10^. The coding sequences of the human proteins that did not record a significant BLAST hit against any of the other primate genomes were used as queries in BLAT searches of the chimpanzee, orangutan genomes to identify orthologous sequences. Non-human primate sequences that contained a frame-shift or premature stop codon that prevented the translation of a protein of at least 80% of the size of the human predicted proteins were considered to be non-protein coding. BLASTN searches with the human coding sequences against the nr (non redundant) database in the NCBI were used to identify matching expressed mRNA sequences. EST database download from UCSC (http://genome.ucsc.edu/) was also searched by BLASTN for expression evidence. We searched two proteomics databases: PRIDE [20] and PeptideAtlas [21] (2010-05), to determine whether a candidate de novo-originated gene had known exact match peptide data. The peptides in these proteomic databases had been identified by a variety of methods from diverse healthy cells, tissues, and fluids.

### Characterization of Expression Patterns for New Genes by RNA–Seq

The recently developed RNA-seq technique has proven to be a powerful approach to detect the expression of genes [23]. RNA-seq data from 11 human tissues: adipose, whole brain, breast, colon, heart, liver, lymph node, skeletal muscle and testes were obtained [41] and downloaded from NCBI with accession code GSE12946, and from cerebral cortex and lung from [42] with NCBI accession code GSE13652. Only reads that mapped to a unique location in the genome were considered. Since the exact transcriptional units of these new genes has not been defined, the expression level of the genes was defined as the numbers of unique reads mapping to the coding region divided by the length of the coding region. Expression levels were normalized by dividing by the total number of valid reads in the samples. Expression levels of 19,800 human genes evaluated by RNA-seq data described above in the 11 tissues, which were obtained from study [43], were used to evaluate genome wide expression pattern. In the study the expression level of a gene in a tissue was defined by the number of valid hits to the gene divided by the effective length of the gene, then was normalized by dividing the total number of valid hits in the tissue [43].

### Calculation of Evolutionary Rate in the Human Lineage

To calculate the evolutionary rates of sequence we used an approach similar to that used in a previous study [44]. Human protein sequences were used to identify one-to-one orthologous genes with BLASTP searches against the chimpanzee and orangutan protein sequences. Reciprocal searches were performed using the chimpanzee and orangutan proteins to query the human proteins to confirm orthology. A total of 16,126 proteins with reciprocal best hits in both human/chimpanzee and human/orangutan searches were retained for further analysis. Orthologs with sequences containing “X” amino acid for “N” in the coding sequences were excluded. Sequences of orthologs were aligned by ClustalW [45]. To exclude incorrect alignments and nonorthologus regions from alignments, we used a sliding window of 5 amino acids, moved the sliding window by one codon for each step, to examine the quality of the alignments. If the aligned human and chimpanzee sequences within a window have a similarity ≤20%, then the orthologs were discarded. Finally, protein sequence with the longest amino acid alignments were retained for each gene, and alignments containing <100 amino acids were discarded. A total of 14,050 one-to-one orthologous genes among human, chimpanzee, and orangutan were identified. The baseml program, implemented in the PAML package, with the HKY85 substitution model was used to calculate the substitution rates in the human and chimpanzee lineage for each gene [46]. Genes that had a substitution rate on the human or chimpanzee lineage of greater than 0.1 were discarded.

## Supporting Information

Figure S1 Our pipeline to search for de novo originated protein-coding genes in human genomes based on protein sequences that were present in previous versions of the human genome (Ensembl versions 40–55) but no longer present in version 56. BLASTP searches of human protein sequences against proteins of other primates identified human 892 genes without protein orthologs. After excluding these genes having no start or stop codons, 741 human coding sequences were used in BLAT searches to find orthologous genes in chimpanzee and orangutan and these sequences were examined to confirm the presence of disrupting mutations. 139 genes with disrupted open reading frames in chimpanzee and orangutan were examined to identify those with human-specific mutations that generate intact open reading frames, resulting 90 candidates. These genes were used as queries of mRNA and proteomic databases to confirm transcription and translation. The pipeline yielded 33 additional de novo originated protein-coding genes that had not been identified in Figure 1.
(PDF)

Figure S2 Mean expression level of de novo originated genes in 11 tissues. The mean expression level is defined by the numbers of unique reads mapped to all the coding regions divided by the total length of the coding regions, in 11 tissues.
(PDF)

Figure S3 Proportion of genes having second (A), third (B), and fourth (C) highest expression levels in each tissue.
(PDF)

Figure S4 Levels of expression of de novo genes in 11 tissues normalized by the level of genome wide genes. (A) The values are the mean normalized expression levels of de novo originated genes divided by the mean normalized expression levels of genome wide genes in 11 tissues. (B) The values are the proportion of the de novo originated genes that have expressed reads divided by the proportion of the genome wide genes that have expressed reads in the 11 tissues. (C) The values are the proportion of the de novo originated genes having their highest normalized expression levels divided by the proportion of the genome wide genes having their highest normalized expression levels in the 11 tissues. (D) The values are the mean expression levels of de novo originated genes divided by the mean expression levels of genome wide genes in 11 tissues.
(PDF)

Figure S5 Two genes with special expression patterns. The mean normalized expression levels of de novo originated genes in 11 tissues are defined by the mean level of expression as the numbers of unique reads mapping to coding regions divided by the total length of all the coding regions, divided by the total number of valid reads in the samples (×10^−8^).
(PDF)

Table S1 27 de novo originated protein-coding genes based on human proteins in Ensembl version 56.
(DOC)

Table S2 33 de novo originated protein-coding genes identified based on human protein-coding genes listed in Ensembl versions 40–55 but deleted in version 56.
(DOC)

Dataset S1 The distribution of length of short proteins encoded by the chimpanzee and orangutan sequences orthologous to human de novo-originated protein coding genes. (A) The length of short proteins encoded by the chimpanzee and orangutan sequences. (B) The distribution of the values of length of short proteins encoded by the chimpanzee sequences divided by human protein length. (C) The distribution of the values of length of short proteins encoded by the orangutan sequences divided by human protein length.
(DOC)

Dataset S2 Alignments of 27 de novo genes from human, chimpanzee, and orangutan sequences.
(DOC)

Dataset S3 Protein evidence for the 27 de novo genes in Table S1.
(DOC)

Dataset S4 Alignments of 33 de novo genes for human, chimpanzee, and orangutan sequences.
(DOC)

Dataset S5 Protein evidence for the 33 de novo genes in Table S2.
(DOC)
