# Supplementary figures and images for: De Novo Origin of Human Protein-Coding Genes
Source: PLoS Genet. 2011 Nov 10;7(11):e1002379. doi: 10.1371/journal.pgen.1002379 (PMC3213175; doi:10.1371/journal.pgen.1002379)

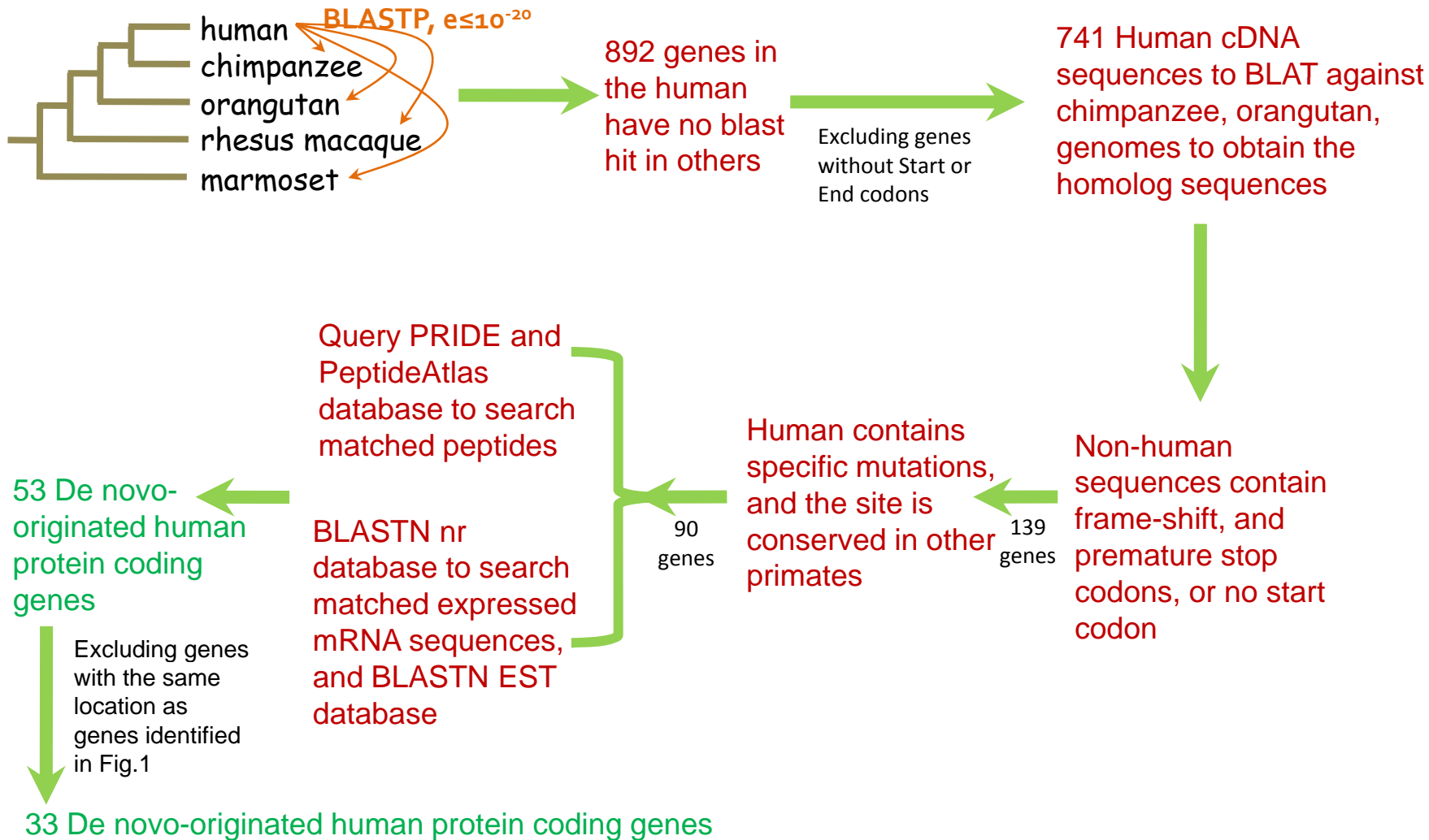

Supplement: Figure S1 — Our pipeline to search for de novo originated protein-coding genes in human genomes based on protein sequences that were present in previous versions of the human genome (Ensembl versions 40–55) but no longer present in version 56. BLASTP searches of human protein sequences against proteins of other primates identified human 892 genes without protein orthologs. After excluding these genes having no start or stop codons, 741 human coding sequences were used in BLAT searches to find orthologous genes in chimpanzee and orangutan and these sequences were examined to confirm the presence of disrupting mutations. 139 genes with disrupted open reading frames in chimpanzee and orangutan were examined to identify those with human-specific mutations that generate intact open reading frames, resulting 90 candidates. These genes were used as queries of mRNA and proteomic databases to confirm transcription and translation. The pipeline yielded 33 additional de novo originated protein-coding genes that had not been identified in Figure 1. (PDF) [file pgen.1002379.s001.pdf]

Expression level

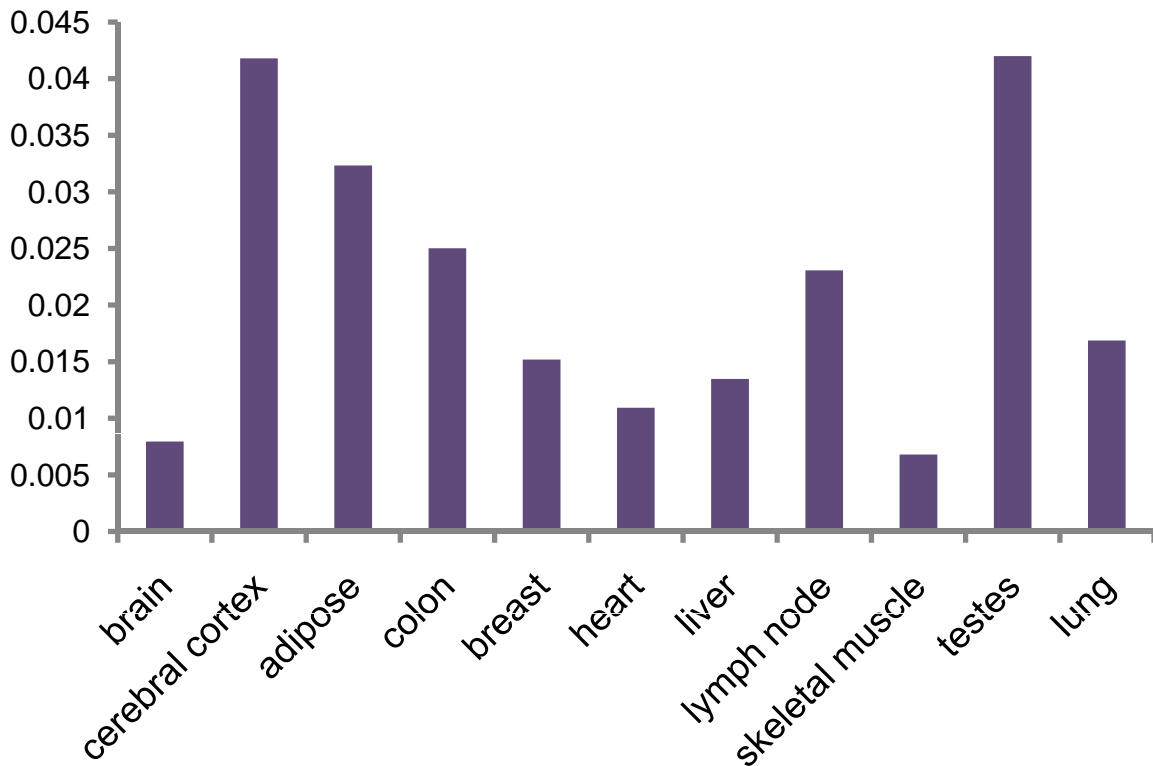

Supplement: Figure S2 — Mean expression level of de novo originated genes in 11 tissues. The mean expression level is defined by the numbers of unique reads mapped to all the coding regions divided by the total length of the coding regions, in 11 tissues. (PDF) [file pgen.1002379.s002.pdf]

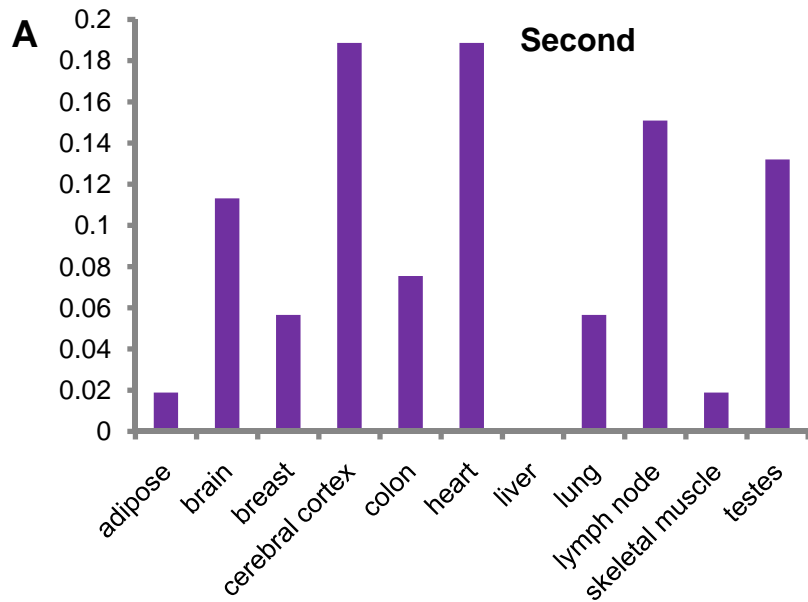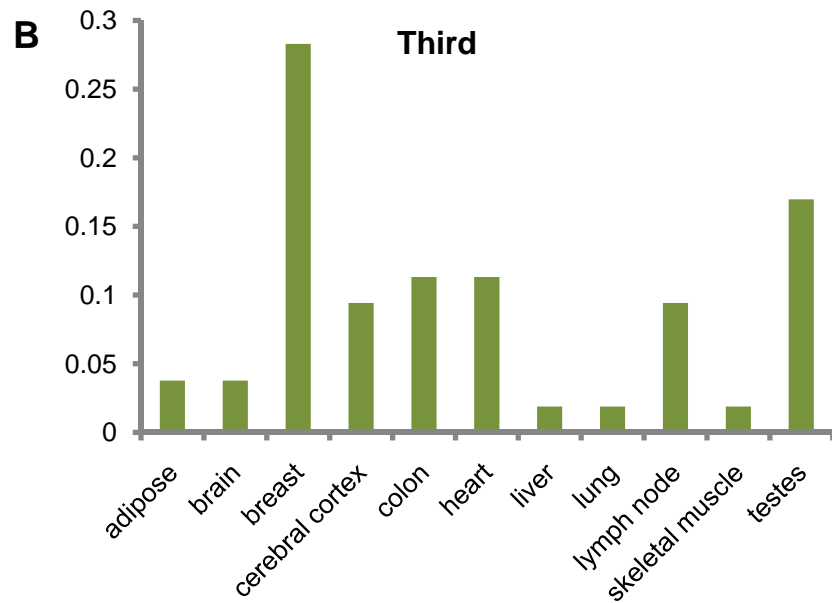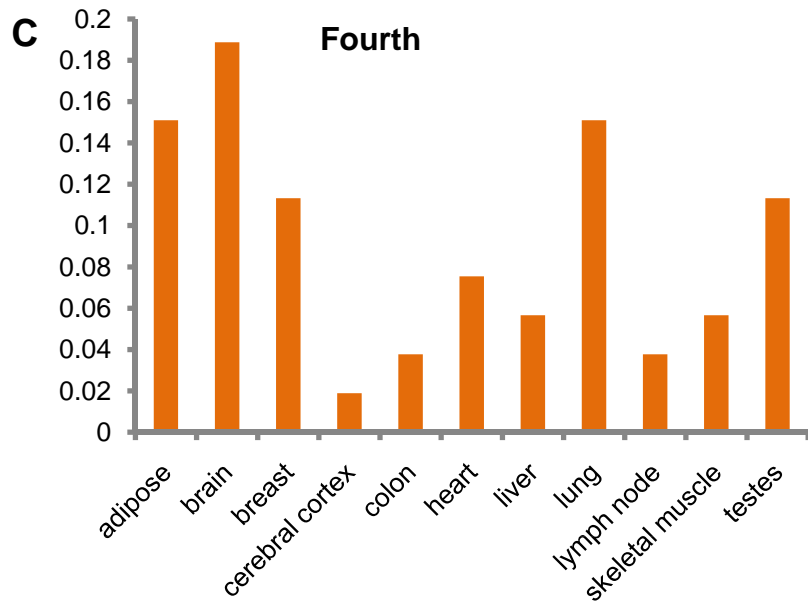

Supplement: Figure S3 — Proportion of genes having second (A), third (B), and fourth (C) highest expression levels in each tissue. (PDF) [file pgen.1002379.s003.pdf]

**ENSG00000206028**

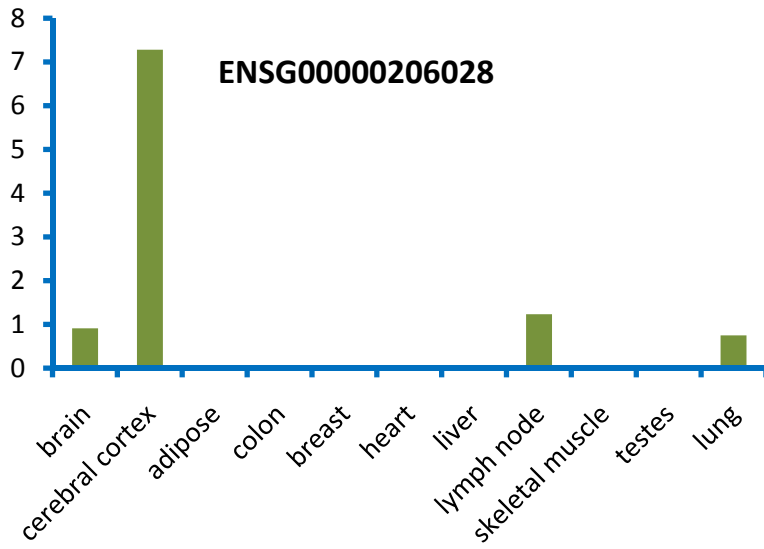

**ENSG00000187488**

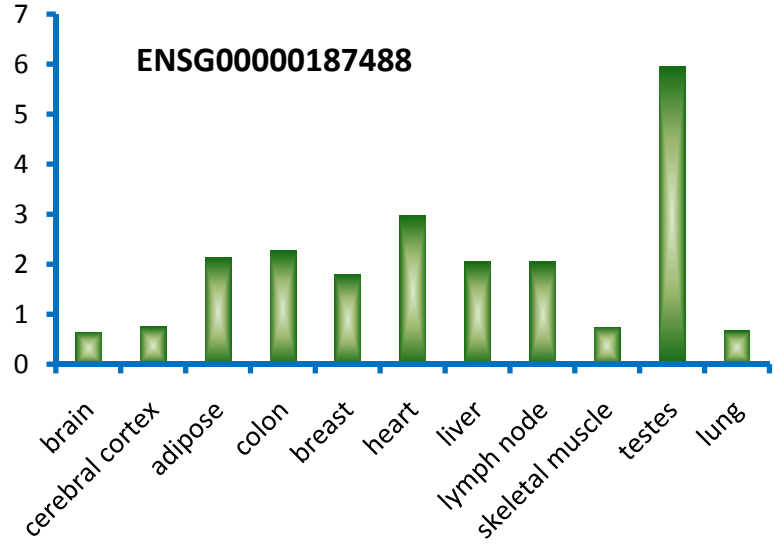

Supplement: Figure S5 — Two genes with special expression patterns. The mean normalized expression levels of de novo originated genes in 11 tissues are defined by the mean level of expression as the numbers of unique reads mapping to coding regions divided by the total length of all the coding regions, divided by the total number of valid reads in the samples (×10−8). (PDF) [file pgen.1002379.s005.pdf]
