# Supplementary material for: De Novo Origin of Human Protein-Coding Genes
Source: PLoS Genet. 2011 Nov 10;7(11):e1002379. doi: 10.1371/journal.pgen.1002379 (PMC3213175; doi:10.1371/journal.pgen.1002379)
Supplement: Figure S4 — Levels of expression of de novo genes in 11 tissues normalized by the level of genome wide genes. (A) The values are the mean normalized expression levels of de novo originated genes divided by the mean normalized expression levels of genome wide genes in 11 tissues. (B) The values are the proportion of the de novo originated genes that have expressed reads divided by the proportion of the genome wide genes that have expressed reads in the 11 tissues. (C) The values are the proportion of the de novo originated genes having their highest normalized expression levels divided by the proportion of the genome wide genes having their highest normalized expression levels in the 11 tissues. (D) The values are the mean expression levels of de novo originated genes divided by the mean expression levels of genome wide genes in 11 tissues. (PDF) [file pgen.1002379.s004.pdf]

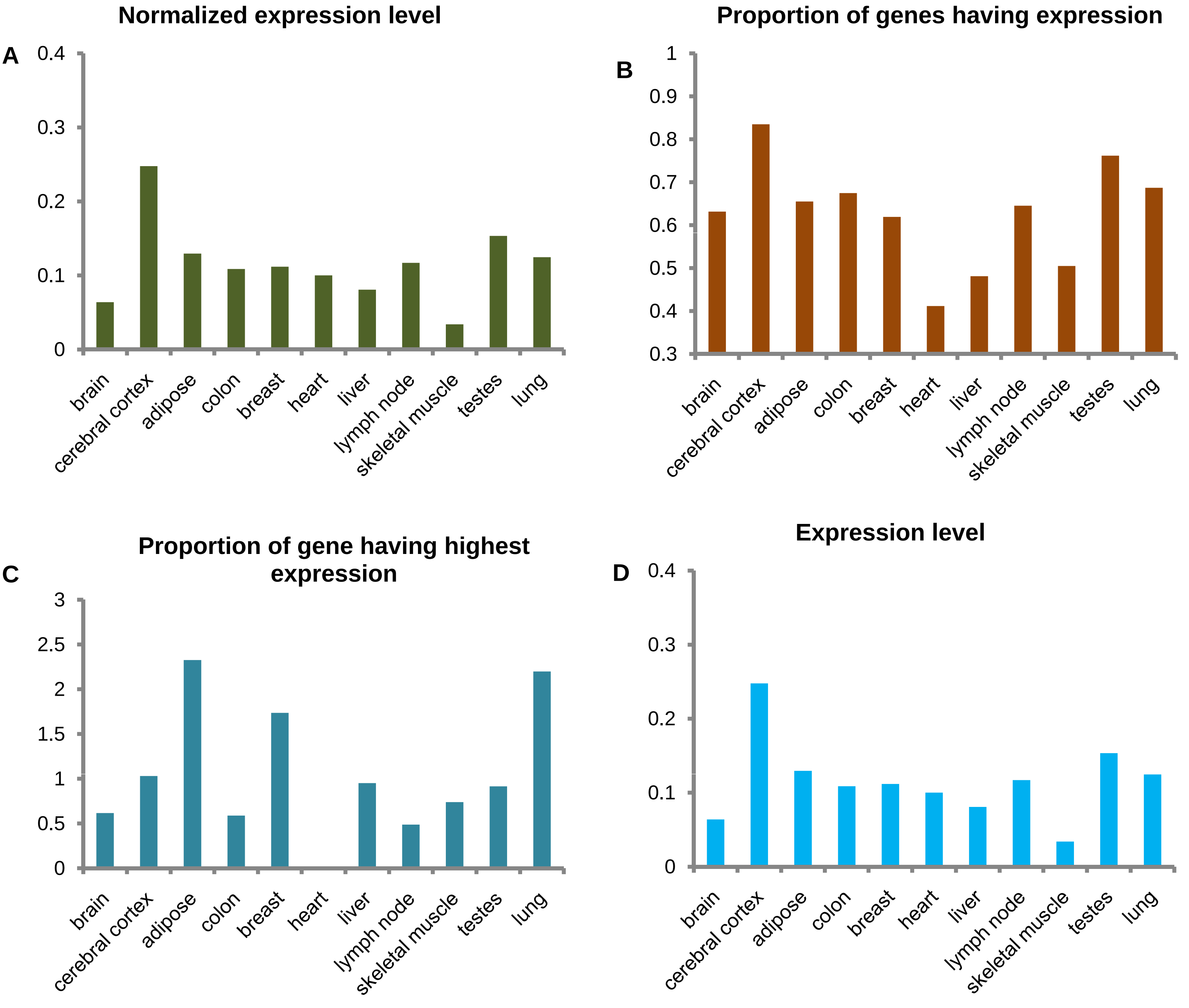
Normalized expression level
A
0.4
0.3
0.2
0.1
0
brain
cerebral cortex
adipose
colon
breast
heart
liver
lymph node
skeletal muscle
testes
lung
Proportion of genes having expression
B
1
0.9
0.8
0.7
0.6
0.5
0.4
0.3
brain
cerebral cortex
adipose
colon
breast
heart
liver
lymph node
skeletal muscle
testes
lung
Proportion of gene having highest expression
C
3
2.5
2
1.5
1
0.5
0
brain
cerebral cortex
adipose
colon
breast
heart
liver
lymph node
skeletal muscle
testes
lung
Expression level
D
0.4
0.3
0.2
0.1
0
brain
cerebral cortex
adipose
colon
breast
heart
liver
lymph node
skeletal muscle
testes
lung
