# Supplementary material for: De Novo Origin of Human Protein-Coding Genes
Source: PLoS Genet. 2011 Nov 10;7(11):e1002379. doi: 10.1371/journal.pgen.1002379 (PMC3213175; doi:10.1371/journal.pgen.1002379)
Supplement: Table S1 — 27 de novo originated protein-coding genes based on human proteins in Ensembl version 56. (DOC) [file pgen.1002379.s006.doc]

Table S1: 27 de novo originated protein coding genes based on the human proteins in Ensembl version 56.

| **Protein ID** | **Transcript ID** | **Gene ID** | **Chr** | **Protein Length** | **Num** | **Gene Expression Evidences** |
| --- | --- | --- | --- | --- | --- | --- |
|  |  |  |  |  | **Exons** |  |
| ENSP00000322899 | ENST00000315678 | ENSG00000176723 | 16 | 348 | 1 | AK291063.1 (/cell line="NT2", /cell type="teratocarcinoma") |
|  |  |  |  |  |  | BC036762.1 (/tissue type="Ovary, pooled from 3 adults") |
| ENSP00000393917 | ENST00000440429 | ENSG00000225917 | 4 | 269 | 1 | BC132927.1(/tissue type="Pooled, cerebellum, kidney, placenta, testis, lung,  colon, liver, heart, thyroid, bladder, uterus, PCR rescued clones") |
|  |  |  |  |  |  | AK096972.1(/tissue type="small intestine") |
| ENSP00000372300 | ENST00000382849 | ENSG00000206113 | 4 | 213 | 1 | AK126028.1 (tissue type="testis") |
| ENSP00000404966 | ENST00000416759 | ENSG00000232330 | 9 | 210 | 1 | AK124777.1 tissue type="brain" |
| ENSP00000381948 | ENST00000398976 | ENSG00000214780 | 11 | 195 | 1 | AK130906.1 /tissue type="uterus" |
| ENSP00000405518 | ENST00000413098 | ENSG00000203393 | 15 | 181 | 1 | AY358254.1/Genome Res (2003) 10:2265-2270 |
| ENSP00000415083 | ENST00000441895 | ENSG00000225860 | 17 | 175 | 1 | BC132910.1 /tissue type="Brain, cerebellum, PCR rescued clones" |
|  |  |  |  |  |  | AK124832.1 /tissue type="corpus callosum" |
| ENSP00000386219 | ENST00000408895 | ENSG00000221972 | 3 | 165 | 1 | BC082257.1 /tissue type="Spleen" |
|  |  |  |  |  |  | BC051274.1 /tissue type="Spleen" |
|  |  |  |  |  |  | AK225383.1 /tissue type="kidney" |
|  |  |  |  |  |  | AK025826.1 /cell type="primary human renal epithelial cells" |
| ENSP00000404239 | ENST00000412222 | ENSG00000224013 | 11 | 164 | 1 | BC132896.1 /tissue type="Lung, PCR rescued clones" |
|  |  |  |  |  |  | BC132894.1 /tissue type="Lung, PCR rescued clones" |
|  |  |  |  |  |  | AK093779.1 /tissue type="thymus" |
| ENSP00000397469 | ENST00000443383 | ENSG00000227520 | 14 | 146 | 1 | AK125200.1 /tissue type="brain" |
| ENSP00000358486 | ENST00000369474 | ENSG00000203863 | 6 | 144 | 1 | AK130540.1 /tissue type="salivary gland" |
| ENSP00000405667 | ENST00000440128 | ENSG00000235766 | 19 | 142 | 1 | AK027130.1 /tissue type="human small intestine" |
| ENSP00000406884 | ENST00000439540 | ENSG00000229811 | 7 | 132 | 1 | BC130590.1 /tissue type="Brain, cerebellum, PCR rescued clones" |
|  |  |  |  |  |  | BC130592.1/tissue_type="Brain, cerebellum, PCR rescued clones" |
|  |  |  |  |  |  | AK054626.1 /tissue type="adrenal gland" |
| ENSP00000387468 | ENST00000426719 | ENSG00000223857 | 16 | 131 | 1 | AK097891.1 /tissue type="thymus" |
|  |  |  |  |  |  | BC029609.1 /tissue type="Brain, hypothalamus" |
| ENSP00000389120 | ENST00000428272 | ENSG00000230294 | 12 | 119 | 1 | BC093897.1 /tissue type="Brain, heart and lung, PCR rescued clones" |
|  |  |  |  |  |  | BC093899.1 /tissue type="Brain, heart and lung, PCR rescued clones" |
|  |  |  |  |  |  | AK098523.1 /tissue type="testis" |
| ENSP00000368218 | ENST00000378936 | ENSG00000205148 | 15 | 126 | 1 | AK128633.1 /tissue type="trachea" |
| ENSP00000354151 | ENST00000360899 | ENSG00000196273 | 14 | 105 | 1 | BC132989.1/tissue type="Brain, cerebellum, PCR rescued clones" |
|  |  |  |  |  |  | BC132991.1/tissue type="Brain, cerebellum, PCR rescued clones" |
|  |  |  |  |  |  | AK094097.1/tissue type="adrenal gland" |
| ENSP00000381811 | ENST00000398830 | ENSG00000214707 | 5 | 244 | 1 | EST evidence |
| ENSP00000390297 | ENST00000429826 | ENSG00000224377 | X | 243 | 1 | BC067869.1/tissue_type="Placenta, pre-eclamptic" |
|  |  |  |  |  |  | BC112979.1/tissue_type="PCR rescued clones" |
|  |  |  |  |  |  | BC112980.1/tissue_type="PCR rescued clones" |
|  |  |  |  |  |  | BC092504.1/tissue_type="Placenta, pre-eclamptic" |
|  |  |  |  |  |  | BC104165.1/tissue_type="PCR rescued clones" |
| ENSP00000412543 | ENST00000445321 | ENSG00000237858 | 8 | 211 | 1 | BC046439.1/tissue_type="Testis" |
|  |  |  |  |  |  | AK092777.1/tissue_type="small intestine" |
| ENSP00000375471 | ENST00000391613 | ENSG00000212929 | 14 | 167 | 1 | AK124214.1/tissue_type="thymus" |
| ENSP00000403473 | ENST00000421885 | ENSG00000236314 | 14 | 156 | 1 | AK026211.1/tissue_type="human small intestine" |
| ENSP00000398567 | ENST00000451773 | ENSG00000237270 | 7 | 154 | 1 | EST evidence |
| ENSP00000316302 | ENST00000313693 | ENSG00000175913 | 11 | 147 | 1 | BC006134.1/tissue_type="Brain, neuroblastoma" |
|  |  |  |  |  |  | BC001183.1/tissue_type="Brain, neuroblastoma" |
| ENSP00000413472 | ENST00000425314 | ENSG00000225021 | 16 | 144 | 1 | AK055679.1/cell_type="neuroblastoma" |
| ENSP00000367798 | ENST00000378537 | ENSG00000205066 | 12 | 129 | 1 | AK094261.1/tissue_type="cerebellum" |
|  |  |  |  |  |  | AK057179.1/tissue_type="stomach" |
|  |  |  |  |  |  | BC072431.1/tissue_type="Brain, hippocampus" |
|  |  |  |  |  |  | BX648969.1/tissue_type="human endometrium" |
| ENSP00000399923 | ENST00000446051 | ENSG00000227316 | 22 | 128 | 1 | AK074445.1/tissue_type="human lung" |

Note: Greens in Expression Evidences are evidences in testis tissues, and reds are evidences in brain tissues.
