# Supplementary material for: De Novo Origin of Human Protein-Coding Genes
Source: PLoS Genet. 2011 Nov 10;7(11):e1002379. doi: 10.1371/journal.pgen.1002379 (PMC3213175; doi:10.1371/journal.pgen.1002379)
Supplement: Table S2 — 33 de novo originated protein-coding genes identified based on human protein-coding genes listed in Ensembl versions 40–55 but deleted in version 56. (DOC) [file pgen.1002379.s007.doc]

Table S2: 33 de novo originated protein coding genes identified based on the human protein coding genes listed in Ensembl version 40-55 but deleted in version 56.

| **Protein ID** | **Transcript ID** | **Gene Ensembl ID** | **Chr** | **Protein** | **Num** | **Expression Evidences** |
| --- | --- | --- | --- | --- | --- | --- |
|  |  |  |  | **Length** | **Exons** |  |
| ENSP00000353931 | ENST00000360706 | ENSG00000187488 | 1 | 221 | 1 | BC072454.1/tissue_type="Peripheral Nervous System, dorsal root ganglion" |
| ENSP00000355096 | ENST00000361187 | ENSG00000183853 | 1 | 208 | 1 | AK090554.1/cell_type="normal astrocytes (NHA5732)" |
| ENSP00000358480 | ENST00000369468 | ENSG00000203862 | 10 | 187 | 1 | AK125829.1/tissue_type="testis" |
| ENSP00000351197 | ENST00000358421 | ENSG00000198447 | 9 | 178 | 1 | AK096255.1/cell_type="teratocarcinoma" |
| ENSP00000371954 | ENST00000382514 | ENSG00000205965 | 4 | 175 | 1 | AK126123.1/tissue_type="thymus" |
| ENSP00000375163 | ENST00000328030 | ENSG00000184827 | 1 | 173 | 1 | AK092728.1/cell_type="neuroblastoma" |
| ENSP00000341745 | ENST00000343077 | ENSG00000188745 | 16 | 167 | 1 | AK092895.1/tissue_type="spleen" |
| ENSP00000372086 | ENST00000382641 | ENSG00000206028 | 22 | 164 | 1 | AK127256.1/tissue_type="hippocampus" |
|  |  |  |  |  |  | AK124820.1/tissue_type="caudate nucleus" |
| ENSP00000366008 | ENST00000376812 | ENSG00000204626 | 12 | 163 | 1 | AK127211.1/tissue_type="hippocampus" |
| ENSP00000348710 | ENST00000356354 | ENSG00000198411 | 14 | 160 | 1 | BC136666.1/tissue_type="Brain, cerebellum, PCR rescued clones" |
|  |  |  |  |  |  | AK127179.1/tissue_type="corpus callosum" |
|  |  |  |  |  |  | BX648898.1/tissue_type="human amygdala" |
| ENSP00000384404 | ENST00000403504 | ENSG00000218478 | 22 | 158 | 1 | AK022809.1/cell_type="teratocarcinoma" |
|  |  |  |  |  |  | BC042614.1/tissue_type="Colon,adenocarcinoma" |
| ENSP00000341266 | ENST00000342892 | ENSG00000204380 | 2 | 155 | 1 | AK126351.1/tissue_type="trachea" |
| ENSP00000300458 | ENST00000300458 | ENSG00000167117 | 17 | 154 | 1 | AK222717.1/tissue_type="colon" |
|  |  |  |  |  |  | AK000701.1/tissue_type="ileal mucosa" |
| ENSP00000295075 | ENST00000295075 | ENSG00000162968 | 2 | 151 | 1 | AK291368.1/tissue_type="brain" |
|  |  |  |  |  |  | AK290476.1/tissue_type="brain" |
|  |  |  |  |  |  | BC113374.1/tissue_type="Cerebellum, Uterus, Testis, PCR rescued clones" |
|  |  |  |  |  |  | BC101782.1/tissue_type="Cerebellum, Uterus, Testis, PCR rescued clones" |
|  |  |  |  |  |  | BC035902.1/tissue_type="Brain, fetal, whole pooled" |
|  |  |  |  |  |  | AK056625.1/tissue_type="brain" |
| ENSP00000364151 | ENST00000375012 | ENSG00000204292 | 17 | 150 | 2 | BC038218.1/tissue_type="Lymph, lymphoma" |
| ENSP00000370085 | ENST00000380709 | ENSG00000205557 | 14 | 149 | 1 | AK125389.1/tissue_type="brain" |
| ENSP00000365352 | ENST00000376181 | ENSG00000204506 | 2 | 147 | 1 | AK125870.1/tissue_type="testis" |
| ENSP00000361987 | ENST00000372896 | ENSG00000204079 | 9 | 141 | 1 | AK025326.1/tissue_type="colon" |
| ENSP00000368465 | ENST00000379167 | ENSG00000205201 | 15 | 140 | 1 | AK124891.1/tissue_type="hippocampus" |
| ENSP00000364923 | ENST00000375768 | ENSG00000204412 | 9 | 138 | 1 | AK126399.1/tissue_type="uterus" |
| ENSP00000321715 | ENST00000318084 | ENSG00000176911 | 10 | 134 | 1 | AK021432.1/tissue_type="whole embryo, mainly head" |
| ENSP00000326475 | ENST00000326801 | ENSG00000180838 | 8 | 131 | 1 | AK092432.1/tissue_type="placenta" |
| ENSP00000372293 | ENST00000382842 | ENSG00000206110 | 16 | 129 | 1 | AK127296.1/tissue_type="hippocampus" |
| ENSP00000381414 | ENST00000398378 | ENSG00000214467 | 12 | 129 | 1 | AK128146.1/tissue_type="testis" |
| ENSP00000258741 | ENST00000258741 | ENSG00000136242 | 7 | 128 | 1 | AK000313.1/cell_type="hepatoma" |
| ENSP00000372255 | ENST00000382805 | ENSG00000206096 | 21 | 127 | 1 | AK125043.1/tissue_type="thalamus" |
| ENSP00000365778 | ENST00000376593 | ENSG00000204581 | 2 | 127 | 1 | BC132790.1/tissue_type="Testis,PCR rescued clones" |
|  |  |  |  |  |  | BC132794.1/tissue_type="Testis,PCR rescued clones" |
|  |  |  |  |  |  | AK125994.1/tissue_type="testis" |
| ENSP00000369345 | ENST00000380008 | ENSG00000205424 | 21 | 126 | 1 | AK123852.1/cell_type="teratocarcinoma" |
| ENSP00000325683 | ENST00000315897 | ENSG00000176833 | 10 | 126 | 1 | AK097743.1/tissue_type="testis" |
| ENSP00000366385 | ENST00000377180 | ENSG00000204707 | 19 | 277 | 1 | AK130324.1/tissue_type="prostate" |
| ENSP00000375203 | ENST00000391387 | ENSG00000212693 | 2 | 131 | 1 | AK095653.1/tissue_type="brain" |
| ENSP00000353493 | ENST00000360340 | ENSG00000197916 | 16 | 129 | 1 | AK097851.1/tissue_type="testis" |
| ENSP00000369183 | ENST00000379854 | ENSG00000205373 | 9 | 219 | 1 | EST evidence |

Note: Greens in Expression Evidences are evidences in testis tissues, and reds are evidences in brain tissues.
