# Supplementary material for: De Novo Origin of Human Protein-Coding Genes
Source: PLoS Genet. 2011 Nov 10;7(11):e1002379. doi: 10.1371/journal.pgen.1002379 (PMC3213175; doi:10.1371/journal.pgen.1002379)
Supplement: Dataset S1 — The distribution of length of short proteins encoded by the chimpanzee and orangutan sequences orthologous to human de novo-originated protein coding genes. (A) The length of short proteins encoded by the chimpanzee and orangutan sequences. (B) The distribution of the values of length of short proteins encoded by the chimpanzee sequences divided by human protein length. (C) The distribution of the values of length of short proteins encoded by the orangutan sequences divided by human protein length. (DOC) [file pgen.1002379.s008.doc]

**Dataset S1:**

**The distribution of length of short proteins encoded by the chimpanzee and orangutan sequences orthologous to human de novo-originated protein coding genes.**

(A) The length of short proteins encoded by the chimpanzee and orangutan sequences.

| **human genes** | **chimpanzee** | **orangutan** |
| --- | --- | --- |
| ENSG00000176723 | 57 | 98 |
| ENSG00000225917 | 120 | 176 |
| ENSG00000206113 | no start codon | no start codon |
| ENSG00000232330 | 106 | 102 |
| ENSG00000214780 | 68 | 68 |
| ENSG00000203393 | 108 | 89 |
| ENSG00000225860 | 117 | 117 |
| ENSG00000221972 | 72 | 58 |
| ENSG00000224013 | no start codon | no start codon |
| ENSG00000227520 | 116 | 42 |
| ENSG00000203863 | 75 | 76 |
| ENSG00000235766 | 91 | 76 |
| ENSG00000229811 | no start codon | no start codon |
| ENSG00000223857 | 16 | 16 |
| ENSG00000230294 | 42 | 42 |
| ENSG00000205148 | 32 | 32 |
| ENSG00000196273 | 24 | 24 |
| ENSG00000214707 | 116 | 50 |
| ENSG00000224377 | 101 | 13 |
| ENSG00000237858 | 85 | 21 |
| ENSG00000212929 | 66 | 104 |
| ENSG00000236314 | 20 | 20 |
| ENSG00000237270 | 4 | 4 |
| ENSG00000175913 | no start codon | no start codon |
| ENSG00000225021 | 110 | 121 |
| ENSG00000205066 | 55 | 55 |
| ENSG00000227316 | no start codon | no start codon |
|  |  |  |
| ENSG00000187488 | 127 | no start codon |
| ENSG00000183853 | 20 | 156 |
| ENSG00000203862 | 12 | 12 |
| ENSG00000198447 | 7 | 7 |
| ENSG00000205965 | 129 | 129 |
| ENSG00000184827 | 90 | 90 |
| ENSG00000188745 | no start codon | no start codon |
| ENSG00000206028 | 107 | 108 |
| ENSG00000204626 | 89 | 90 |
| ENSG00000198411 | 33 | 24 |
| ENSG00000218478 | 87 | 65 |
| ENSG00000204380 | 64 | 64 |
| ENSG00000167117 | 11 | 18 |
| ENSG00000162968 | no start codon | no start codon |
| ENSG00000204292 | 67 | 74 |
| ENSG00000205557 | 97 | 99 |
| ENSG00000204506 | 69 | 69 |
| ENSG00000204079 | 84 | 70 |
| ENSG00000205201 | 69 | 69 |
| ENSG00000204412 | 13 | 13 |
| ENSG00000176911 | 44 | 12 |
| ENSG00000180838 | 64 | 64 |
| ENSG00000206110 | 18 | 53 |
| ENSG00000214467 | 66 | 66 |
| ENSG00000136242 | 84 | 82 |
| ENSG00000206096 | 61 | 61 |
| ENSG00000204581 | 64 | 64 |
| ENSG00000205424 | 3 | 3 |
| ENSG00000176833 | 59 | 59 |
| ENSG00000204707 | 170 | 170 |
| ENSG00000212693 | no start codon | no start codon |
| ENSG00000197916 | 3 | 3 |
| ENSG00000205373 | 58 | 58 |

(B) The distribution of the values of length of short proteins encoded by the chimpanzee sequences divided by human protein length.

(C) The distribution of the values of length of short proteins encoded by the orangutan sequences divided by human protein length.
