# Supplementary material for: De Novo Origin of Human Protein-Coding Genes
Source: PLoS Genet. 2011 Nov 10;7(11):e1002379. doi: 10.1371/journal.pgen.1002379 (PMC3213175; doi:10.1371/journal.pgen.1002379)
Supplement: Dataset S2 — Alignments of 27 de novo genes from human, chimpanzee, and orangutan sequences. (DOC) [file pgen.1002379.s009.doc]

Dataset S2: alignments of 27 new genes for human, chimpanzee, and orangutan sequences. The red bold are sites where mutations occur specifically in humans helping to generate a completed protein coding frame, but are conserved in chimpanzee and orangutan.

**ENSG00000176723**
human ATGAGAAGCCTCCCCTTTGCCCTGACTGTGGAAAGCGTTTCAGCCAGAGCTCCTACCTGCTGCAGCACCGGCAGATTCACCCAGGGCCGTCAGCCCTGCAAGTGCAAGGCCTGCGGGAGGGGTTTTACTCAGAGCGCATC
chimpanzee ATGAGAAGCCTCCCCTTTGCCCTGACTGTGGAAAGCGTTTCAGCCAGAGCTCCTTCCTGCTGCAGCACCGGCAGATTCACCCAGGGCCGTCAGCCCTGCAAGTGCAAGGCCTGCGGGAGGGGTTTTACTCAGAGCGCATT
orangutan GTGAGAAGCCTTCCCCCTGCCCTGACTGTGGAAAGCGTTTCAGCCAGAGCTCCGACCTGCTGCAGCACCGGCAGATTCACCCGGGGCCGTCAGCCCTGCAAGTGCAGGGCCTGCGGGAGGGGTTTTACTCAGAGCGCATC

human CCTCCTCCAGCACTGGCGGGTCCACAGCGACTGGCGAGAGACCTTGTCGCTGTCCCCAGTGCGGCAAGACCTGCTGTGGCCGCTCCAACCTCATCAAGCACCAGCGAGTCCACTCGGGAGAAGCCATTCGTCTGCGGG-A
chimpanzee CCTCCTCCAGCACTGGCGGGTCCACAGCGACTGACGAGAGACCTTGTCGCTGTCCCCAGTGCGGCAAGACCTGCTGTGGCCGCTCCAACCTCATCAAGCACCAGCGAGTCCACTCGGGAGAAGCCATTCGTCTGCGGG-A
orangutan CCTCCTCCAACACTGGCGGGTCCATAACGACTGGCGAGAGACCTTATCGCTGTCCCCAGTGCGGCAAGACCTGCTGTGGCCGCTCCAACCTCATCAAGCACCAGCGAGTCCACTCGGGAGAAGCCATTCGTCAGCGGGGA

human GTGCGGCAAGGGTTTAGCGGCCAGCTCTGCTGCTGGCTCACCAAGGAGCACACACTGGCCGAAGCCCTGCGTCTGTCCCCAGTACCAGCAGGTTTTTGGGGACCGGTGGAAGCTGATCGGCCACCAGCGAATTCACACAG
chimpanzee GTGCGGCAAGGGTTTAGCGGCCAGCTCTGCTGCTGGCTCACCAAGGAGCACACACTGGCCGAAGCCCTGCGTCTGTCCCCAGTACCAGCAGGTTTTTGGGGACCGGTGGAAGCTGATCGGCCACCAGCGAATTCACACAG
orangutan GTGCGGCAAGGGCTTAGCGGCCAGCTCTGCTGCTGGCTCACCAA---GCACACACTG------GCCCTGCGTCTGTCCCCAGTGCCAGCAGGTTTTTGGGGACCGGTGGAAGCTGATCTGCCACCAGCGAATTCACACAG

human GAGAGTGT**GT**CCCTTCTGCTGCTGCAGCTGCGGTGACAGTGTCAATGAGAAGACCTCCCTCTCCCAGCGCGTCCTTCCGCACCCAGGGGAGAAGACCTGCAGGGGTGGGAGCGTGGAGAGCGTCAGCCTCGCACCCAGCT
chimpanzee GAGAGTGT**--**CCCTTCTGCTGCTGCAGCTGCGGTGACAGTGTCAATGAGAAGACCTCCCTCTCCCAGCGCGTCCTTCCGCACCCAGGGGAGAAGACCTGCAGGGGTGGGAGCGTGGAGAGCGTCAGCCTCGCACCCAGCT
orangutan GAGAGCGT**--**CCGTTCTGCTGCTGCAGCTGCGGTGACAGCGTCAATGAGAAGACCTCCCTCTCCCAGCGCGTCCTCCCGCACCCAGGGAAGAAGACCTGCAGGCGTGGGAGCGTGGAGAGCGTCATCCTCGCACCCAGCT

human CAGTCGCCCCGGACAGCACCTCTGGGCTCCGGCCCTGTGGGAGCCCCGGGTCCTTCCTTCAACATCTGCCCCCATCCACACTCCTGCCCCGACCTCCCTTCCTTTATCCTGGCCCCCCACTCAGTCTCCAGCCTCTGGTT
chimpanzee CAGTCGCCCCGGACAGCACCTCCGGGCTCCGGCCCTGTGGGAGCCCCGGGTCCTTCCTTCAACATCTGCCCCCATCCACACTCCTGCCCCGACCTCCCTTCCTTTATCCTGGCCCCCCACTCAGTCTCCAGCCTCTGGTT
orangutan CAGTCGCCCCGGGCAGCACCTCCGGGCTCCGGCCCTTTGGGAGCCCCGGGTCCTTCCTTCAACGTCTGCCCCCATCCACACTCCTGCCCCGACCTCCCTTCCTTTATCCTGGCCCCCCACTCAGTCTCCAGCCTCTGGTT

human CCATCCGGCCTTCCCGCAGTGCCTGCAGTGCCTCTGGGAGGCCTGGAAGTTGCCCAGGTTCCGCCAGCCACCCAGCCGGCAGCTCAGCAGGAGGGGGCGATGGGGCCCAGAAGCTGTGCGAGCGCGGGACGGGACTCGCG
chimpanzee CCATCCGGCCTTCCTGCAGTGCCTGCAGTGCCTCCGGGAGGCCTGGAAGTTGCCCAGGTTCCGCCAGCCACCCAGCCGGCAGCTCAGCAGGAGGGGGCGATGGGGCCCAGAAGCTGTGCGAGCGCGGGACGGGACTCGCG
orangutan CCATCCGGCCTTCCCG-------------GCCTCCGGGAGGCCTGGAAGTTGCCCAGGTTCCGCCAGTCACCCAGCCGGCAGCTCAGCAGGAGGGGGCGATGGGGCCCAGAAGCTGTGCGAGCGCGGGACGGGACTTGCG


human GGAGGCGGTTCAGGCCCCAGGCTACCCTGAGCCAGCCCGGAAGGCCTCGCAGCACAGAGCGGCCGGACCGCTCGGCGAGGCCAGGGCCAGGCTTCAGCGACAGTGCCGAGCTCCTCCACCGCCCTCGAACCCACACTGGA
chimpanzee GGAGGCGGTTCAGGCCCCAGGCTACCCTGAGCCAGCCCGGAAGGCCTCGCAGCACAGAGCGGCCCGACCGCTCGGCGAGGCCAGGGCCAGGCTTCAGCGACAGTGCCGAGCTCCTCCGCCGCCCTCGAACCCACACTGGA
orangutan GGAGGCGGTTCAGGCCCCAGGCTACCCTGAGCCAGCCCGGGAGGCCTCGCAGCACAGAGCGGCCCGATCGCTCGGCGAGGCCAGGGCCAGGCTTCAGCGACAGTGCTGAGCTCCTCCGCCACCCTCGAACCCACACTGGA


human GAGGAGCCCTACCAGTGTTTCCGGTGTGGAAGGCCTCCAGCCGGAGGTCCAACCTGGCCCGACACTGA
chimpanzee GAGGAGCCCTACCAGTGTTTCCGGTGTGGAAGGCCTCCAGCCGGAGGTCCAACCTGGCCCGACACTGA
orangutan GAGGAGCCCTACCAGTGTTTCCGGTGTGGAAGGCCTCCAGCCGGAGGTCCAACCTGGCCCGACACTGA

**ENSG00000225917**
human ATGCAGCCGGGCTGCGCGGTCCCGCAGAGCGGGCGCCTCCGGGGCTCGAGTCGGGGACCCGGCAGGAGCGGGCCGGGGATGGCGGCGGCGGGCGGCGGGTCGGCCGTGGAGCCCCGGAGAGGCGGCCGCCACCACCGCTG
chimpanzee ATGCAGCTGGGCTGCGCGGTCCCGCAGAGCGGGCGCCTCCGGGGCTCGGGTCGGGGACCCCGCAGGAGCGGGCCGGGGATGGCGGCGGCGGG-------TCGGCCGTGGAGCCCCGGGGAGGCGGCCGCCACCACCGCTG
orangutan ATGCAGCCGGGCTGCGCGCTCGCGCAGAGCGGGCGCCTCCGGGGCTCGGGTCGGGGACCGGACAGGAGCGGGCCGGGGATGGCGGCGGCGGGCGGCGGGTCGGCCGTGGAGCCCCGGGGAGGCGGCCGCCACCACTGCTG

human CCCGCAGTCTGCGAGGTGTCCGGCGGTCCGCTCAGGGCCTCTTTTGCAGACTCGGTGCCCGGAGTGCGCCGGCGCCGCCCGCCAGGTCTTGGCACTGCTGGCGCGGCCGCGGCGGCGGGGGCCCGGCTCCAGATCACCGA
chimpanzee CCTGCAGTCTGCGAGGTGTCCGCTGGCCCGCTCAGGGCCTCTTTTGGAGACTCGGTGCCCGGAGTGCGCCGGCGCCGCCCGCCAGGTCTTGGCACTGCTGGCGCGGCCGCGGCGGCGGGGGCCCGGCTCCAGATCACCGA
orangutan CCCGCAGTCTGCGAGGTGTCCGGCGGCCCGCTCAGGGCCCCTTTTGCAGACTCGGTGCCCGGAGTGCGCCGGCGCCGCCCGCCAGGTCTTGGCACTGCTGGCGCGGCCGCGGCGGCGGGGGCCCGGCTCCAGATCCCAGA

human CCCCCACGAGCGCCGGCCCTGCCTGCCCGCTATTTTGGTTCCATCTCTTCTGCATATTTATGCTTGCCTGTTACATTCCCTGGCCTCTGAGTGTCCTTTGGCAGTTTCCAAAGCATTTGGGCAAGAAGGAGCTTGGGCAG
chimpanzee CCCCCACGAGCGCCGGCCCTGCATGCCCGCTATTTTGGTTCCATCTCTTCTGCCTATTTATGCTTGCCTGTTACATTCCCTGGCCTCTGAGTGTCCTTTGGCAGTTTCCAAAGCATTTGGGCAAGAAGGAGCTTGGGCAG
orangutan TCCCCGCGAGCGCCGGCCCTGCGTGCCCGCTATTTTGGTTCCATCTCTTCTGCATATTTATGCTTGCCTGTCACATTCCCTGGCCTCTGAGTGTCCTTTGGCAGTTTCCAA-GCATTTGGGCAAGAAGGAGCTTGGGGAG

human CTTCACAACAGGTGGGGACGCGCAGGGGAGGGGCCGCGGCCGGACCGGCAGA**--**GTTTCAAGGGCTGGCGCGCCGGCGGACTTTCCACACTGTCTTTCGGGACTGGGGTTAGGAGACAGCCCTTGGCCACCACTGTGTCC
chimpanzee CTTCACAACAGGTGGGGACGCGCAGGGGAGGGGCCGCGGCCGGACCGGCAGA**GA**GTTTCAAGGGCTGGCGCGCCGGCGGACTTTCCACACTGTCTTTCGGGACTGGGGTTAGGAGACAGCCCTTGGCCACCACTGTGTCC
orangutan CTTCACAACAGGTGGGGACGCGCAGGGGAGGGGCCGCGGCCGGACCAGCAGA**GA**GCTTCAAGGGCTGGCGCGCCGACGGGTTTTCCACACTGTCTTTCGGGACTGGGGTTAGAAAACAGCCCTTGGCTACCACTGTGTCC

human TTACTTTGTCGCTTTACTTGGGGCTGGACCTCCCCTTTCCAGCTCTGGCGTGTGACCCCGCAACGAGCGTGGTGTCGAGACCACCACCAGCTGGGGCGGTGGGAGCGAAGGTGCCCCAGGCTCGGGGAAGGTGGAAACCT
chimpanzee TTACTTTGTCGCTTTACTTGGGGCTGGACCTCCCCTTTCCAGCTCTGGCGTGTGACCCCGCAACGAGCGTGGTGTCGAGACCACCACCAGCTGGGGCGGTGGGAGCGAAGGTGCCCCAGGCTCGGGGAAGGTGGAGACCT
orangutan TTACTCTGTCGCTTTACTTGGGGCTGGACCTCCCCTTTCCAGCTCTGGCGTGTGACCCCGCACCGAGCGTGGTGTCGAGACCACCACCAGCTGGGGCGGTGGGAGCGAAGGTGCCCCAGGCTCGGGGAAGGTGGAGACCT

human TTTCCCCGCTCCCCAAGTGATTGTTGCGCGCTCCGGTGAGGGGACAGTGGTACCTCGATTTCCCGTGTGCCAGCTTCGGGACTCAGCTGTGCTTTTGTGTCTCTTTGATTAG
chimpanzee TTTCCCCGCTCCCCAAGTGATTGTTGCGCGCTCCGGTGAGGGGACAGTGGTACCTCGATTTCCCGTGTGCCAGCTTCGGGACTCAGCTGTGCTTTTGTGTCTCTTTGATTAG
orangutan TTTCCCCGCTCCCCAAGTGATTGTTGCGCGCTCCGGTGAGGGGACAGTGGTACCTCGATTTCCCGTGTGCCAGCCTCGGGACTCAGCTGTGCTTTTGTGTCTCTTTGATTAG

**ENSG00000206113**
human **ATG**GGGAGGTGCTCCTGGCACCCAGAATGTGTGTCAGGCCAGGCTCTGGTGAAAGAAGCCTTGGCCGGGACCCGGGATATGACATCCACACTGCGGTTCCATCCTCAGTCAACACAGATGAGAAGGGTCAGCCCTGGAGC
chimpanze **ATA**GGGAGGTGCTCCTGGCACCCAGAATGTGTGTCAGGCCAGGCTCTGGTGAAAGAAGCCTTGGCCGGGACCCGGGATATGACATCCACACTGCAGTTCCATCCTTAGTCAACACAGATGAGAAGGGTCAGCCCTGGAGC
orangutan **ATA**GGGAGGTGCTCCTGGCACCCAGAATGTGTGTCAGGCCAGGCTCTGGTGAAAGAAGTCTTGGCCAGAACCCGGGATATGACATCCACACTGCGGTTCCATCCTCAGTTAACACAGACAAGAAGGGTCAGCCCTGGAGC

human GCCCCCATGCCCCACCCCCACTTTGGGGGGCATCTTATCTCGGGAAATGGGTCCTCCATCCCCCAGGCGCCCCAGGGCTGTGGCAGTGAGGGTGCGCAAGGCCACAACTTGTGCTGTATTTGTGGTAACCGAGACTTGGG
chimpanze GCCCCCATGCCCCACCCCCACTTTGGGGGGCATCTTATCTCGGGAAATGGGTCCTCCATCCCCCAGGTGCCCCAGGGCTGTGGCAGTGAGGGTGCGCAAGGCCACAACTTGTGCTGTATTTGTGGTAACCGAGACTTGGG
orangutan GCCCCCATGCCCCACCCCCACTTTGGGGGGCATCTTATCTCGGGAAATGGGTCCTCCATCCCCCAGGCGCCCCAGGGCTGTGGCAGTGAGGGTGCACAAGGCCACAACTTGTGCTGTATTTGTGGTAACTGAGACTTGGG

human AATCACTGACTGGGTCTCCGACAGAGGCAGGGGCTGGCCTGGGCAGTGAGGCTCCCGGGGAGCCGAGGGCTGCAGGCTTCTGTACACACCTCCTTCATCTTCCTACCCACACCTGGAAGAAGTCACAGGCCCACCTGGAG
chimpanze AATCACTGACTGGGTCTCCGACAGAGGCAGGGGCTGGCCTGGGCAGTGAGGCCCCTGGGGAGCCGAGGGGTGCAGGCTTCTGTACACACCTCCTTCATCTTCCTACCCACACCTGGAAGAAGTCACAGGCCCACCTGGAG
orangutan AATCACTGACCGGGTCTCCGACAGAGGCAGGG-CTGGCCTGGGCAGTGAGGTGCCCGGGGAGCCGAGGGCTGCAGTTGTCCGTACACAC-TCCTTCATCTTCCTACC-ACAC-TGGAG--AGTCACAG------------

human GCTGCCCAACTCCTCAGCTCCTGTCCTCCCATCCCTGGGTTTTCCTCACAGCTCCATCCTGGTCCTGGCACCCCCTGCAACCTCGGCAGCCCTGCCCCACTCAGGGGCCTCCCTGTCACCTGGTCTCAGCTCCCACCACG
chimpanze GCTGCCCAACTCCTCAGCTCCTGTCCTCCCATCCCTAGGTTTTCCTCACAGCTCCATCCTGGTCCTGGCACCCCCTGCAACCTCGGCAGCCCTGCCCCACTCAGGGGCCTCCCAGTCACCTGGTCTCAGCTCCCGCCCCG
orangutan --------------------------------------------------------------------------------------------------------------------------------------------

human AGGTGCTGATCTCACATCCACCACCTGTCAGTGGCATGTGCCTGGGGAACAGGAGCTTGGATTGCAGGGCCCCTGCCTCTAG
chimpanze AGGGGCTGATCTCACATCCACCACCTGTCAGTGGCATGTGCCTGGGGAACAGGAGCTTGGATTGCAGGGCCCCTGCCTCTAG
orangutan ----------------------------------------------------------------------------------

**ENSG00000232330**
human ATGCCCAGGAGCCTGCGGCCCCCTTGTCCTGGATCTACTCTGCGCTGGCTTCCAGGAGGGAGGACCCCCTTCCCCCACCACGTCTCATGCCAGCCTCGGCGCAGCTCCGGAGAGCGGGAGGCGGAGGCTCAGAGCGGTGC
chimpanzee ATGCCCAGGAGCCTGCGGCCCCCTTGTCCTGGATCTACTCTGCGCTGGCTTCCAGGAAGGAGGACCCCCTTCCCCCACCACGTCTCATGCCAGCCTCGGCGCAGCTCCGGAGAGCGGGAGGGGGAGGCTCAGAGCGGTGC
orangutan ATGCCCAGGAGCCCGCGGCCACCTTGTCCTGGATCTACTCTGCGCTGGCTTCCAAGAAGGAGGAACCCCTTCCCCCACCACGTCTCGTGCCAGGCTCGGCGCAGCTCCGGAGAGCGGGAGGGGGAGGCTCAGAGCGGTGC

human AGCCCCACCGGGCCCCAGCCCGTTGCCTCCGCCCCCACCTCACCCC-ATCCCCAGCAGCACCACTTCCGCTCAGGCCTGGCTGCTGGCAAAATCTCGGCACAGAGGGAGGAGGGGGAGAGGAAAACGCATGATTCCTCCT
chimpanzee AGCCCCACCGGGCCCCAGCCCGTTGCCTCAGCCCCCCCCTCACCCC-ATCCCCAGCAGCACCACTTCCGCTCAGGCCTGGCTGCTGGCAAAATCTCGGCACAGAGGGAGGAGGGGGAGAGGAAAACGCGTGATTCCTCCT
orangutan AGCCCCGCCGGGCCCCAGCCCGTCGCCTCAGCCCCCACCTCACCCCCATCCCCAGCAGCACCACTTCCGCTCAGGCCTGGCTGCTGGCAAAATCTCGGCACAGAGGGAGGAGGGGGAGAGGAAAACGCATGATTCCTCCT

human CAAAATGGAGTCAGCCGAAAAAAGCGTGAATGCAGAGCC**CGA**AGAGACTCCTGGGGGAGGGGAGCCCCTGCA-GGGCCAGCCGAGGGCCGGCGCAATGG----CTTATCTGAGGGACAGGCAGAAGGACGGACCCCCACG
chimpanzee CAAAATGGAGTCAGCCGAAAAAAGCGTGAATGCAGAGCC**TGA**AGAGACTCCTGGGGGAGGGGAGCCCCTGCA-GGGCCAGCCGAGGGCCGGCGCAATGG----CTTATCTGAGGGACAGGCAGAAGGACGGACCCCCAAG
orangutan CAAAATGGAGTCAGCCGAAAAAAGCGTGAATGCAGAGCC**TGA**AGAGACTCCTGGGGGAGGGGAGCCCCTGCAAGGGCCAGCCGAGGGCCGGCGCAATGGGAGGCTCATCTGAGGGACAGGGAGAGGGACGGACCCCCACG

human GTGGACCCCAGCTACGCACCGTGTCGTGGT-GGGGCGGGAAGGCGAAGGTGTACTCGTCTGCCAGCAGCCTGCGGTAGGCGTAGTCCTCGTGCGCGCGGCTCCCGTAAGCCCCGTAGTAGTCGTACTCGAGGACCTGGGA
chimpanzee GTGGACCCCAGCTACGCACCGCGTCGTGGT-GGGGCGGGAAGGCGAAGGTGTACTCGTCTGCCAGCAGCCTGCGGTAGGCGTAGTCCTCGTGCGCGCGGCTCCCGTAAGCCCCGTAGTAGTCGTACTCGAGGACCTGGGA
orangutan GTGGACCCCAGCGACGCACCGTGTCGTGGTTGGGGCGGGAAGGCGAAGGTGTACTCGTCCGCCAGCAGCCTGCGGTAGGCGTAGTCCTCGTGCACGCGGCTCCCGTAAGCCCCGTAGTAGTCGTACTCGAGGACCTGGGA

human AGAAAAGACGTGGTCCTCAGCCTGCCTCTTTGGCCCCTCCCCGCTTCCCTCCCCAGAGCGGGGTCCCGCTGAGGCTGTGA--
chimpanzee AGAAAAGACGAGGTCCTCAGCCTGCCTCTTTGGCCCCTCCCCGCTTCCCTCCCCAGAGCGGGGTCCCGCTGAGGCTGTGA--
orangutan AGAAAAGACGTGGTCCTCAGCCTCCCTCTTTGGTCCCTCCCCGCTTCTCTCCCCAGAGCGGGGTCCCGCTGAGGCTTTGTGA

**ENSG00000214780**
human ATGAACTCTGTGTTCAGACAGCCTGGCTCCAACTTCAGCTTCCCCATTTATCAGCCGTCTGCCTCGGTAACCCATGGCCTCAGTTTCTTCTGTGAAGTGAGCCCTCAAATGCTTACCCCTCAAGATTATCGTGGGAAGCA
chimpanzee ATGAACTCTGTGTTCAGACAGCCTGGCTCCAACTTCAGCTTCCCCATTTATCAGCTGTCTGCCTTGGTAACCCATGGCCTCAGTTTCTTCTGTGAAGTGAGCCCTCAAATGCTTACCCCTCAAGATTATCGTGGGAAGCA
orangutan ATGAACTCTGTGTTCAGACAGCCTGGCTCCAACTTCAGCTTCCCCATTTATCAACCCTCTGCCTCGGTAACCCATGGCCTCAGTTTCTTCTGTGGAGTGAGCCCTCAAATGCTTACCCCTCAAGATTATCGTGGGAAGCA

human A**-**CAAGGGCACAGAGGATGCCCTGGGTGCATGGTGGCCATGGACACATGGCCGGCCGTGCTTCCTGAGCTCCCTGGCTTTTGGGGAGAAGGGAGTGGAGGCCAGGCTTGGGGTGCCAGGGGTGAACCTTTGCTGCAGAAG
chimpanzee A**G**CAAGGGCACAGAGGATGCCCTGGGTGCATGGTGGCCATGGACACATGGCCGGCCGTGCTTCCTGAGCTCCCTGGCTTTTGGGGAGAAGGGAGTGGAGGCCAGGCTTGGGGTGCCAGGGGTGAACCTTTGCTGCAGAAG
orangutan A**G**CAAGGGCACAGAGGACACCCTGGGTGCATGGTGGCCACGGACACATGGCTGACCGTGCTTCCTAAGCTCCCTGGCTTCTGGGGAGAAGGGAGTGGAGGCCAGGCTTGGGGTGCCAGGGGTGAACCTTTGCTGCAGAAG

human GCAAACTGTTTGCTTGAGGCTGTGAGGCTGTTCCTAAACTACAGGCCCCTGGGGGCAGAGGGAGGAGAACTAACATTTGGTAAGCGCCTGCTGGTGCCCACTGCTGTATCATGTGCCTATGATTGCGTGAGCTCATCAGC
chimpanzee GCAAACTGTTTGCTTGAGGCTGTGAGGCTGTTCCTAAACTACAGGCCCCTGAGGGCAGAGGGAGGAGAACTAACATTTGGTAAGCGCCTGCTGGTGCCCGCTGCTGTATCATGTGCCTATGATTGCGTGAGCTCATCAGC
orangutan GCAAACTGTTTGCTTGAGGCTGTGAGGCTGTTCCTAA-CTACAGGCCCCTGGGGGCAGAGGGAGGAGAACTAACATTTGGTAAGCGCCTGCTGGTGCCCGCTGCTGTATCATGTGCCTATGATTGCGTGAGCTCATCAGC

human CTTCCTGGGTATCCAGCTCCTGGGAGACCCCACAAGGAGCCCTGGCCACTGCTACCGTCAGGATTTTAATGGCACACTGAGGCTCAGAGAGGGAAAGGGGCTTGTCCAGAGCCACACAGGGACTCTGTGGGCAGGGCCAC
chimpanzee CTTCCTGGGTATCCAGCTCCTGGGAGACCCCACAAGGAGCCCTGGCCACTGCTCCCGTCAGGATTTTAATGGCACACTGAGGCTCAGAGAGGGAAAGGGGCTTGTCCAGAGCCACACAGGGACTCTGTGGGCAGGGCCAC
orangutan CTTCCTGGGTATCCAGCTCCTGGGAGACCCCACAAGGAGCCCTGGCCACTGCTCCCGTCAGGATTTTAATGGCACACTGAGGCTCAGAGAGGGAAAGGGGCTTGTCCAGAGCCACACAGGGACTCTGTGGCCAGGGCCAC

human TCCAGGAAGGGACCCAGGGCCAGACCTGA
chimpanzee TCCAGGAAGGGACCCAGGGCCAGACCTGA
orangutan TCCAGGGAGGGACCCAGGGCCAGGCCTGG

**ENSG00000225860**
human ATGCCTCAGTGGCTGGTGGCGTCCATCATTCCTGGGTGTGTCTGTGAGAGTGTTTCCAGAGGAGACTCACATGTGAGCCAGCGGGCTGAGAAGGAGACCCGTTCTCAGTGTGAGTGTGCACTGTCCAATCAGCTCAAGGC
chimpanzee ATGCCTCAGTGGCTGGTGGCGTCCACCATTCCTGGGTGTGTCTGTGAGAGTGTTTCCAGAGGAGACTCACATGTGAGCCAGCGGGCTGAGAGGGAGACCCGTTCTCAGTGTGAGTGTGCACTGTCCAATCAGCTCAAGGC
orangutan ATGCCTCAGTGGCTGGTGGAGTCCATTATTCCTGGGTGTGTCTGTGAGAGTGTTTCTAGAGGAGACTCACATGTGAGCCAGCGGGCTGAGAGGCAGACCCGTTCTCAGTGTGAGTGTGCACCATCCAATCAGCTCAAGGC

human CAGGCTGGG**G**ACAAACAGGCAGAAGAAGGAGGATTCTCTCTCCCACCTTTCTGGAGCAGGATGCCTTTTCTCCTTGGACATCAGACTACAGGGTCTTTGGCTTTTGGATTCTAGGACTTGTACCAATGGCCTCCCGGGGC
chimpanzee CAGGCTGGG**-**ACAAACAGGCAGAAGAAGGAGGATTCTCTCTCCCGCCTTTCTGGAGCAGGATGCCTTTTCTCCTTGGACATCAGACTACAGGGTCTTTGGCTTTTGGATTCTAGGACTTGTACCAACGGCCTCCCAGGGC
orangutan CAGGCTGGG**-**ACAAACAGGCAGAAGAAGGAGGATTCTCTCTCCCTGCTTTCTGGAGCAGGATGCCTTTTCTCCTTGGACATCAGACTACAGGGTCTTTGGCTTTTGGATTCTAGGACTTGTACCAATGGCCTCCCGGGGC

human CCTCAGGCCTTCAGCCTCCAACGAAGGTCTGTGCTGTTGGCCTCCCTGATTCTGAGGCTTCTGGACTTGGACTGAGGCATGCTACGGGCTTCTCTGATTCTCCAGCTTGTGGATGGCCTATCATGGGACTTCTCCACCTC
chimpanzee CCTCAGGCCTTCAGCCTCCAACGAAGGTCTGTGCTGTTGGCCTCCCTGATTCTGAGGCTTCCGGACTTGGACTGAGGCATGCTACGGGCTTCTCTGATTCTCCAGCTTGTGGATGGCTTATCATGGGACTTCTCCGCCTC
orangutan CCTCAGGCCTTCAGCCTCCAAGGAAGGTCTGTGCTGTTGGCCTCCCTGATTCTGAGGCTTCCAGACTTGGACTGAGGCATGCTATGGGCTTCTCTGATTCTCCAGCTTGTGGATGGCCTATCATGGGACTTCTCCGCCTC

human TGTAATCACAAGGGCCAATGCCCCCTAATACCTTTCTTTTCATATATCCTACTGGTTCTGTCTGCCTGGGGAACCTTGACTAATACAGATATGGAGCATTTGAAATGA
chimpanzee TGTAATCACAAGGGCAAATGCCCCCTAATACCTTTCTTTTCATATATCCTACTGGTTCTGTCTGCCTGGGGAACCTTGACTAATACAGATATGGAGCATTTGAAATGA
orangutan TGTAATCACATGGGCCAATGCCCCCTAATACATTTCTTTTCATATATCCTACTGGTTCTGTCTGCCTTGGGAACCTTGACTAATACAGATACGGAGCATTTGAAATGA

**ENSG00000203393**
human ATGATCTTTATGCAAATTCTAGAGCCCCAAGAGGTCCCTAGTTTCTTGATGATCTGCCAAAGGCGCTCACCTGCCATGCACAGGACATGCACGGATCATGCACCCCTAGCCATAGCTCAGGTCTGGCTTTGGGTGAGCCT
chimpanzee ATGATCTTTATGCAAATTCTAGAGCTCCAAGAGGTCCCTAGTTTCTTGATGATCTGCAAAAGGCGCTCACCTGCCATGCACAGGACATGCACGGATCATGCACTCCTAGCCATAGCTCAGGTCTGGCTTTGGGTGAGCCT
orangutan ATGATCTTTATGCAAATTCTAGAGCTCCAAGAGGTCCCTAGTT---TGATGATCTGCAAAAGGCGCTCACCTGCCATGCACAGGACATGCACGGATCATGCACCCCTAGCCATAGCTCAGGTCTGGCTTTGGGTGAGCCT

human GGCC**-**AAGGCTGGAAGCAACAGGAGAGGGCCTGGCAGAGCAGAGGGGACCTTTTTTTCCCTGCTCGCTGCCCTGCATGCTGCTCAGCATTTTCCAAACCTCCCCACTGCTCCT-GGTGGGGCTTCACAATCGAACATTGT
chimpanzee GGCC**C**AAGGCTGGAAGCAACAGGAGAGGGCCTGGCAGAGCAGAGGGGACCTTTTTTTCCCTGCTCGCTGCCCTGCAGGCTGCTCAGCATTTTCCAAACCTCCCCACTGCTCCT-GGGGGGGCTTCACAATCGAACATTGT
orangutan GGCC**C**AAGGCTGGAAGCAAGAGGAGAGGGCCTGGCAGAGCAGAGGGGACCTTTTTTTCCCTGCTAGCCGCCCTGCAGACTGCTCAGCATTTTCCAAACCTCCCCACTGCTCCTCAGGGGGGCTTCACAATTGAACATTGT

human CAGCCCAGAGCTGACACCCAAGCCGACCACAGCCCTAAAGCATGCTGAGTGCCTCTTGGACCTGAATTCCCATAGTCTGTACCGAAAGCCACGCCCCAAGGCTGCAGTTTACCTCAATCTCAGTTTGCCTTTGAAATCTG
chimpanzee CAGCCCAGAGCTGACACCCAAGGCGACCACAGCCCTAAAGCATGCTGAGTGCCTCTTGGACCTGAATTCCCATAGTCTGTACCGAAAGCCACGCCCCAAGGCTGCAGTTTACCTCAATCTCAGTTTGCCTTTGAAATCTG
orangutan CAGCCCAGAGCTGACACCCAAGCTGACCACAACCCTAAAGCATGCTGAGTGCCTCTTGGACCTGAATTCCCATAGTCTGTACCGAAATCCACGCCCCAAGGCTGCGGTTTACCTCAATCTCAGTTTGCCTTTGAAATCTG

human TTCACAGGCTATCTCTGAAAAAGTCCTTTGGGTTTGGAAAACGGGACTTTGAAAACAATTCCGTTTTTATTGTAGATTCAGGGGGCACGTGTGCAGGTTTGTTACCAGGGTATATCGGGTGGTGCTGA
chimpanzee TTCACAGGCTATCTCTGAAAAAGTCCTTTGGGTTTGGAAAACGGGACTTTGAAAACAATTCCGTTTTTATTGTAGATTCAGGGGGCACGTGTGCAGGTTTGTTACCAGGGTATATCAGGTGGTGCTGA
orangutan TTCACAGGCTATCTCTGAAAAAGTCGTTTGGGTTTGGAAAACGGGACTTTGAAAACAATTCCGTTTTTATTGTAGATTCAGGGGGCACGTGTGCAGGTTTGTTACCAGGGTATATTGGGTGGTGCTGA

**ENSG00000221972**
human ATGCAGGCTGAGACCATCCTGGAGGGTCTTGAGGCTGGCTTACCCCAGGCTGTGAGTAGTGGGCTCAGCCTAGTCCCGGCTCCTGGG-TTAGTGCTCACGTGTCTCTCTGCCCCCTCAGGGCCTGGAGGAATGGCCTTAG
chimpanzee ATGCAGGCTGAGACCATCCTGGAGGGTCTTGAGGCTGGCTTACCCCAGGCTGTGAGTAGTGGGCTCAGCCTAGTCCCGGCTCCTGGG-TTAGTGCTCACGTGTCTCTCTGCCCACTCAGGGCCTGGAGGAATGGCCTTAG
orangutan ATGCAGGCTGAGACCATCCTGGAGGGTCTTGAGGCTGGCTTACCCCAGGCTGTGAGTAGTGGGCTCAGCCTAGTCCCGGCTCCTGGGGTTAGTGATCACGTGCCGCTCTGCCCACTCAGGGCCTGGAGGAATGGCCTTAG

human AGCCCCC**-**ACCAACCACGCTCAGGAAGGCATTCCTAGCTCAGAGTACCCTCCTGGAGTCTACACTGGAAGGGGCTCCTGAGTGGGCCGCGCCACACCCCGAGGAGCAGAGGCGCAGTCCTCCCGCGTGCTCCCAGCACAC
chimpanzee AGCCCCC**C**ACCAACCAGGCTCAGGAAGGCATTCCTAGCTCAGAGTACCCTCCTGGAGTCTACACTGGAAGGGGCTCCTGAGTGGGCCGCGCCACACCCCGAGGAGCAGAGGCGCAGTCCTCCCGCGTGCTCCCAGCACAC
orangutan AGCCCCC**C**ACCAACCAGGCTCAGGAAGGCATTCCTAGCTCAGAGTACCCTCCTGGAGTCTACACTGGAAGGGGCTCCTGAGTGGGCCGCGCCACACCCCGAGGAGCAGAGGCGCAGTCCTCCCGCGTACTCCCAGCACAC

human TCCACCCCTACCCAGCACACCCACGGGGCCCCC--ACCTTGCTCACCTGGGGGGAACCACCCACTCTGCGCCCTCTCAG----GGAGAGGTGGTGGCCGCTGCTCTATCC-CCTCCCTCTCTTCCTCTTCCACCTTCTCT
chimpanzee TCCACCCCTACCCAGCACACCCATGGGGCCCCC--ACCTTGCTCACCTGGGGGGAACCACCCACTCTGCGCCCTCTCAG----GGAGAGGTGGTGGCCGCTGCTCTATCC-CCTCCCTCTCTTCCTCTTCCACCTTCTCT
orangutan TCCACCCCTGCCCAGCACACCCATGGGGCCCCCCGACCTTGCTCACCTGGGGGGAACCACCCACTCTGCGCCCTCTCAGCGATGGAGAGGTGGTGGCCGCTGCTCTATCCTCCTCCCTCTCTTCCTCTTCCACCTTCTCT

human CTCTTCTCATCTGGGTGCTGGAACCCAAGGGTGAAACTGAGAGTCAGAAAGTCTCAGTCTCAAGGCAGAGCGGGGCAACTAATTTAA
chimpanzee CTCTTCTCATCTGGGTGCTGGAACCCAAGCGTGAAACTGAGAGTCAGAAAGTCTCAGTCTCAAGGCAGAGCGGGGCAACTAATTTAA
orangutan CTCTTCTCATCTGGGTGCTGGAACCCAAGCGTGAAACTGAGAGTCAGAAAGTCTCAGTCTCAAGGCAGAGCGGGGCAACTAATTTAA

**ENSG00000224013**

human **ATG**ATTGGGAAGGTGGAAGGTGGAGACGCGGTCCTTAGGTGGTCGAGCGCATTTTCTCTTCCTCAGAGTCCAGGAAGGAGCGCGATGGAGAAGTGGGGCGCACGGCGGGTTTCGGGTCCGCCCCTGCCCTTCCGACTCCT
chimpanzee **CAG**ATTGGGAAGGTGGAAGGTGGAGACGCGGTCCTTAGGTGGTCGAGCGCATTTTCTCTTCCTCAGAGTCCAGGAAGGAGCGCGATGGAGAAGTGGGGCGCACGGCGGGTTTCGGGTCCGCCCCTGCCCTTCCGACTCCT
orangutan **CAG**TTTGGGAAGGTGGGAGGTGGAGACGCGGTCCTTAGGTGGTCGAGCGCACTTTCTCTTCCTCAGAGTCCAGGAAGGAGCGCGATGGAGAAGTGGGGCGCACGGCGGGTTTCGGGTCCGCCCCTGCCCTTCCGACTCCT

human CCGACCACGTCTGCCTTTGGCTGAAAGCAAATATGGCTCCGTGTGCGGTTTCTGCAACAAAGGCTGGGCGGGCCGGGTTTCCCTAGGGATGGGGACCGCCTCCCCGGGGAGTCGTGGGGGTGAGCCACCGGGGCCTCCGA
chimpanzee CCGACCACGTCTGCCTTTGGCTGAAAGCAAATATGGCTCCGTGTGCAGTTTCTGCAACAAAGGCTGGGCGGGCCGGGTTTCCCTAGGGATGGGGACCGCCTCCCCGGGGAGTCGTGGGGGTGAGCCACCGGGGCCTCCGA
orangutan CCGACCACGTCTGCCTTTGGCTGAAAGCAAATATGGCTCCGTGTGCAGTTTCTGCAACAAAGGGTGGGCGGGCCGG-TTTCCCTAGGGATGGGGACCGCCTCACCCGGGAGTCGTGGGGGTGAGCCACCGGGGCCTCCGA

human GAGAATCGCTGGTGTCGCTTCGCACCCAAGGGACACATCTCGGGTTAGAAAGGAGAAACGACGTGGATCTAAAGGCGAAGCCCATGCTGAGGACTTTTCAAAATGGCCTCTTATCGGCCCCACTCACAGGAGCTTCAGGC
chimpanzee GAGAATCGCTGGTGTCGCTTCGCACCCAAGGGACACATCTCGGGTTAGAAAGGAGAAACGACGTGGATCTAAAGGCGAAGCCCATGCTGAGGACTTTTCAAAATGGCCTCTTATCGGCCCCACTCACAGGAGCTTCAGGC
orangutan GAGAATCGCTGGTGTCGCTTCGCACCCAAGGGAGACATCTCGGGTTAGAAAGGAGAATCGACGTGGATCTAAAGGCGAAGCCCATGCTGAGGCCTTTTCAAAATGGCCTCTTATCGGCCCCACTCACAGGAGCTTCAGGC

human TTCGGTGGCCCTGTAGGACAACCCTGGGGTGTCATTGGAGAGAAGCTGACGGGGTTAATGGCGGGTGACAGGTGA
chimpanzee TTCGGTGGCCCTGTAGGACAACCCTGGGGTGTCATTGGAGAGAAGCTGACGGGGTTAATGGCGGGTGACAGGTGA
orangutan TTCGGTGGCCCTGTAGGACAACTCTGGGGAGTCATTGGAGAGAAGCTGACGGGGTTAATGGCGGGTGACAGGTGA

**ENSG00000227520**

human ATGCCACGGGGCCTCCGTCCAGGCTGTCCCGTCCGCACGGGTCGACTGGTCACCTTGGAATCCCCTTTGCAGGTCCCAGCGCCCCCCGGGAACCCGCAGCCTCCGCGGAGAGCGTGGGCCTCTCCCTACCGCTGGGGCGC
chimpanzee ATGCCACGGGGCCTCCGTCCAGGCTGTCCCGTCCGCACGGGTCGACTGGTCACCTTGGAATCCCCTTTGCAGGTCCCAGCGCCCCCCGGGAACCCGCAGCCTCCGCGGAGAGCGTGGGCCTCTCCCTGCCGCTGGGGCGC
orangutan ATGCCACGGGGCCTCCGTTCAGGCTGTCCCGTCCGCACGGGTCGACTGGTCACCTTGGAATCCCCTTTGCAGGTCCCAGCGCCCCCCGGGAACCCGCAGCCTCCGCGGAGAGCGTGGGCCTCTCCCTGACGCTGGGGCGC

human AGCGCAGTGCACGCCTGAGGGTGGTCGCCGGGGGCTGGGCAC**-**GCCCCCAGTCCTGCGCCGCCGGGGGCTGCGGCGGTGCTGCCCACCCCAGAGAGCCCTCGGCCTGGGGCTCCGGCGAAGCAAGTGCCTTCCCGGCGCC
chimpanzee AGCGCAGTGCACGCCTGAGGGTGGTCGCCGGGGGCTGGGCAC**C**GCCCCCAGTCCTGCGCCGCCGGGGGCTGCGGCGGTGCTGCCCACCCCAGAGAGCCCTCGGCCTGGGGCTCCGGCGAAGCAAGTGCCTTCCCGGCGCC
orangutan AGGGCAGTGCACGCCTGAGGGTGGTCGCCCGGGGCTGGGCAC**C**GCCCCCAGTCCTGCGCCGCCGGGGGCTGCGGCGGTGCTGCCCACCCCAGAGAGCCCTCGGCCTGGGGCTCCGGCGAAGCAAGTGCCTTCCCCGCGCC

human GGTCGCCAGGGGGGCGCGGGAGCAGCCAGATGCGCCGCAGCGCTGGGAAGGCGGCGAAGGACAGGGGCTAGGGGAGTGAGGGGCGCTCGGCAGGCAGCCTCAGCCCTGGCCCTGCGCGGGAGAAGGGACAGCAGAGACCG
chimpanzee GGTCGCCAGGGGGGCGCGGGAGCAGCCAGATGCGCCGCAGCGCTGGGAAGGCGGCGAAGGACAGGGGCTAGGGGAGTGAGGGGCGCTCGGCAGGCAGCCTCAGCCCTGGCCCTGCGCGGGAGAAGGGACAGCAGAGACCG
orangutan GGTCGCCAGGGGGGCGCGGGAGCAGCCAGATGCGCCGCAGCCCTGGGAAGGCGGCGAAGGACAGGGGCTAGGGGAGTGAGGGGCGCTCGGCAGGCAGCCTCAGCCCTGGCCCTGCGCGGGAGAAGGGACAGCAGAGACCG

human CCCGTGGGGCCCCGGGGTGTAG
chimpanzee CCCGTGGGGCCCCGGGGTGTAG
orangutan CCCGTGGGGCCCCGGGGCGTAG

**ENSG00000203863**
human ATGATCAGGGCAGCCTTACTGGAGCCTCAACCTCCCAAGCTCAAGCAATCCTCTCACCTCAGCCCCCAAGGAGCTGGGACTACAGGTGCAGGCCCCCACACCCCACTAACTTTTTTATACTTTTTTCGTCTGGAACTCCT
chimpanzee ATGATCAGGGCAGCCTTACTGGAGCCTCAACCTCCCAAGCTCAAGCAATCCTCTCACCTCAGCCCCCAAGGAGCTGGGACTACAGGTGCAGGCCACCACACCCCACTAACTTTTTTATACTTTTTTCGTCTCGAACTCCT
orangutan ATGATCAGGGCAGCCTTACTGCAGTCTCAACCTCCCAAGCTCAAGCAATCCTCTCACCTCAGCCCCCAAGGAGCTGGGACTACAGGTGCAGGCCACCACACCCCACTAACTTTTTTATACTTTTTTCATCTCGAACTCCT

human G**----**CAAGCAGCCCTCCCACCTCAGCCTCCCAAAGTGCTG---GTAAGACAGCCAGGTGGGAAGGATTCCTTGGCAGAATCTCCTTTTGACCTGAGCACTGGGAGGAATGCACCCTGTGGTGGAGCCTTGGGAAATTTG
chimpanzee G**GGCT**CAAGCAGCCCTCCCACCTCAGCCTCCCAAAGTGCTG---ATAAGACAGCCAGGTGGGAAGGATTCCCTGGCAGAATCTCCTTTTGACCTGAGCACTGGGAGGAATGCACACTGTGGTGGAGCCTTGGGAAATTTG
orangutan G**GGCT**CAAGCAGTCCTCCCACCTCGGCCTCCCAGAGTGCTGTTAGTAAGACAGCCAGGTGGGAAGGATTCCCTGGCAGAATCTCCTGCTGACCTGAGCACTGGGAGGAATGCGCACTGGGGTGGAGCCTTGGGAAATTTG

human CGCCATTTGCAGTGGGGAGGAGCCTGGCCCCTCCTTTTCCTAGGTGGAACCTGGGATTCAGTCTACCAGGCAGGAAGCACTCTGGCTTTGGGGAGAGTCTCTGTTTCCCTTTTTTTCTTTTTGCCCAATAAATTCCATTC
chimpanzee CGCCATTTGCAGTGGGGAGGAGCCTGGCCCCTCCTTTTCCTAGGTGGAACCTGGGATTCAGTCTACCAGGCAGGAAGCACTCTGGCTTTGGGGAGAGTCTCTGTTTCCCTTTTTTTCTTTTTGCCCGATAAATTCCATTC
orangutan AGCCATTTGCAGTGGGGAGGAGCCTGGCCCCTCCTTTTCCTAGGTGGAACCTGGGATTCAGTCTACCAGGCAGGAAGCACTCTGGCTTTGGGAAGAGTCTCTGTTTCCGTTTTTTTCTTTTTGCCCAATAAATTCCATTC

human TCACCCTTCAAGGCATCTGTGA
chimpanzee TCACCCTTCAAGGCATCTGTGA
orangutan TCACCCTTCAAGGCATCTGTGA

**ENSG00000235766**
human ATGCTCTCGTGCTGCTTGGAGCCAGCAGCGTGGGACTGTGGGGCCGAGGGCAGGGATGGGAGAGAAGAGATGGTTCTGGGCTGGAAGCGAGACAGGGGGACCACTCCCCGCACCCTCCCCGCCAGCCCCAGTGCGGGGAC
chimpanzee ATGCTCTCGTGCTGCTTGGAGCCAGCAGCGTGGGACTGTGGGGCCGAGGGCAGGGATGAGAGAGAAGAGATGGTTCCGGGCTGGAAGCGAGAC--GGGGACCACTCCCCGCACCCTCCCCGCCAGCCCCAGCGCGGGGAC
orangutan GTGCTCTCGTGCTGCTTGGAGCCAGCAGCGTGGGGCTGTGGGGCCGAGGGCAGGGATGGGAGAGAAGAGATGGTTCTGGGCTGGAAGCGAGACAGGGGGACCGCTCCCCGTACCCTCTCCGCCAGCCCCAGCGCTGGGAC

human GCCTCTCTGGGGTGCAGGGCACGTGCTTGGGGACGCTGGCGAGAGCCCCTTACCTTCACATCCGTGTCCGAATCGCTGGAGCTGCTGCTGGAGTCGGAAGAGCTGTGGTGTCCTTGCTGGATGGAGGTGCGGCAGTGAGG
chimpanzee GCCTCTCTGGGGTGCAGGGCACGTGCTTGGGGACGCTGGCGAGAGCCCCTTACCTTCACATCCGTGTCCGAATCGCTGGAGCTGCTGCTGGAGTCGGAAGAGCTCTGGTGTCCTTGCTGGATGGAGGTGCGGCAGTGAGG
orangutan GGCTGTCTGGGGTGCAGGGCACGTGCTTGGGGACGCTGGCGAGAGCCCCTTACCTTCACATCCGTGTCCGAGTCGCTGGAGCTGCTGCTAGAGTCGGAAGAGCTGTGGTGTCCTTGCTGGATGGAGGTGCGGCAGTGAGG

human CGGCGCCCCTTACCCAGCCCCC**T**GAAGTTGGAGGCCTAAGGCAGGACCCTGGGGTCAGGGGCAACCCCAGCCTTCCCGCCCCTCCGCAGCCGGTGATGAGGCGACTTACCTTTGGACCCGGACCTGCCCCTGCCTCCGAC
chimpanzee CGGCGCCCCTTACCCAGCCCCC**-**GAAGTTGGAGGCCCAAGGCAGGACCCTGGGGTCAGGGGCAACCCCAGCCTTCCCGCCCCTCCGCAGCCGGTGATGAGGCGACTTACCTTTGGACCCGGACCTGCCCCTGCCTCCGAC
orangutan CGGTGCCCCTTACCCAGCCCCC**-**GAAGTTGGAAGCCCAAGGCAGGACCCTGGGGTCAGGGGCAACCCCACCCTTCCCGCCCCTCCGCAGCCGGTGATGAGGCGACTTACCTTTGGACCCGGACCTGCCCCTGCCTCCGGC

human CGGCCCTGA
chimpanzee CGGCCCTGA
orangutan CGGCCCTGA

**ENSG00000229811**
human **ATG**CTCCTTCCACGGAAACTGACCCTGTCCTATTTCCTCGCTTTGATCATTGCCCAGAAGGAACAGGGACTGTTGAGAAACAGAGGCAGCGTGCAGCCTGAAGGCATGCACCGTGCAGAAGAGGATCCCTGCGGCTGCGG
chimpanzee **ACG**CTCCTTCCACGGAAACTGACCCTGTCCTATTTCCTCGCTTTGATCATTGCCCAGAAGGGACAGGGACTGTTGAGAAACAGAGGCAGCGTGCAGCCTGAAGGCATGCACCGTGCAGAAGAGGATCCCTGCGGCTGCGG
orangutan **ACG**CTCCTTCCACGGAAACTGACCCTGTCCTATTTCCTCGCTTTGATCATTGCCCAGAAGGGACAGAGACTGTTGAGAAACAGAGGCAGCATGCAGCCTGAAGGCATGCACCATGCAGAAGAGGATCCCTG------CGG

human GCTCTTTTCCAGGCCCAAGGACAAGGCGGTGTGTGCCGGGTTG-ATCTGCACT--GCTTTAGCCAGGATGGCTCTTCTTAGAGAGACAAACCGCTCCAACGCCAAACTGCCCTCGCAATTCCAATCGCAAAGGCAGGCTA
chimpanzee GCTCTTTTCCAGGCCCAAGGACAAGGCAGTGTGTGCCGGGCAGTGTGTGCACT--GCTTTAGCCAGGATGGCTCTTCTTAGAGAGGCAAACCGCTCCAACGCCAAACTGCCCTCGCAATTCCAATCGCAAAGGCAGGCTA
orangutan GCTCTTTTCCAGGCCCAAGGACAAGGCAGTGTGTGCCGGGTTG-ATCTGCACTCTGCTTTAGCCAGGATGGCTCTTCTTAGAGAGGCAAACCGCTCCAATGCCAAACTGCCCTCGCAATTCCAATCGCAAAGGCAGGCTA

human GGGCCACAGTTCAAACCCAACTGCCAATGTCCACAAAGAAAAGAAGAAAGCTGGAAATGCAGTTGGTCTTGCCATGCTGGCCGCTCAGCTTCTCACCCCCAGGCCTAAGAAGGACAAGGTGA
chimpanzee GGGCCACAGTTCAAACCCAACTGCCAATGTCCACAAAGAAAAGAAGAAAGCTGGAAATGCAGTCGGTCTTGCCATGCTGGCCGCTCAGCTTCTCACCCCCAGGCCTAAGAAGGACAAGGTGA
orangutan GGGCCACAGTTCAAACCCAATTGCCAATGTCCACAAAGAAAAGAAGAAAGCTGGAAATGCAGTCGGTCTTGCCATGCTGGCCGCTCAGCTTCTCACCCCCAGGCCTAAGAAGGACGAGGTGA

**ENSG00000223857**
human ATG**--**TCTGGCGGACACAGCTCCCCGGAACCTCCACGCCCATGGCCACTAGACAGAGGGAGTCTTCCTTCACCTCCTGCTTTTCCACCTGGAATTGCGACGCGGGCGACGAGGGCGTGGGCTGCACCTGCGAAGATGCTT
chimpanzee ATG**TC**TCTGGCGGACACAGCTCCCCGGAACCTCCACGCCCATGGCCACTAGACAGAGGGAGTCTTCCTTCACCTCCTGCTTTTCCACCTGGAGTTGCGACGCGGGCGACGAGGGCGTGGGCTGCACCTGCGAAGATGCTT
orangutan ATG**TC**CCTGGCGGACACAGCTCCCCGGAACCTCCACGCCCATGGCCACTAGACAGAGGGCGTCTTCCTTCACCTCCTGCTTTTCCACCTCGAGCTGCGACGCGGACGACGAGGGCGTGCGCGGAACCTGCGAAGATGCTT

human CCTTGTGCAAGAAGCGCCTGTCGGGCGCGGGATTCGGGGCTGGCATCTGGGACGCGGGCTGAGGTGGGAGGCGGGCCTGCATCTGAAGAATACGCTCGGAGGCTGGCAGGTTGCTGCCCCCGCCTCGCACGACCCTCGCT
chimpanzee CCTTGTGCAAGAAGCGCCTGTCGGGCGCGGGATTCGGGGCTGGCATCTGGGACGCGGGCTGAGGTGGGAGGCGGGCCTACATCTGAAGAATACGCTCGGAGGCTGGCAGGTTGCTGCCCCCGCCTCGCACGACCCTCGCT
orangutan CCTTGTGCAAGAAGCGCCTGTCGGGCGCGGGATTCGGGGCTGGCATCTGGGACGCGGGCTTAGTTGGGAGGCGGGCCTGCATCTGAAGAATACGCTCGGAGGCTGGCAGGTTGCTGCCCCCGCCTCGCACGACCTTCGCT

human TCCCACCTGTGAAATGCACAGAACAGGGCTTCATTTATTTAACGAATCGTTTCTGAGCTCCTGCTGTGAGCCAGGCTTGGAGCAAGCCTGGGTACT--GTGGATGGGAGCAGGCCCCTGA------
chimpanzee TCCCACCTGTGAAATGCACAGAACAGGGCTTCATTTATTTAACGAATCGTTTCTGAGCTCCTGCTGTGAGCCAGGCTTGGAGCAAGCCTGGGTACT--GTGGATGGGAGCAGGCCCCTGA------
orangutan TCTCACCTGTGAAATGCACAGAACAGGGCTTCATTTATTTAACGAATCGTTTCTGAGCTCCTGCTGTGAGCCAGGCTTGGAGCAAGCCCCTGAACCAGGCGCACGGGGTCTTGGCGTTCTCGGAGT

**ENSG00000230294**
human ATGACATCCA**A**CCTGGGCTCTCCTGGCCCCCAGAGAAGAGCTGCCCAGTGCATGTGCTGGCTGCACCGTGACGTCCCCTATGAACGAGGCCCACAGGCAGTGGCTGGCTGGGGGGATGCCCCTGCCCTGAGCTGGAGTCT
chimpanzee ATGACATCCA**-**CTTGGGCTCTCCTGGCCCCCAGAGAAGATCTGTCCAGTGCATGTGCTGGCTGCACCGTGACGTCCCCTATGAACAGGGCCCACAGGCAGTGGCTGGCTGGGGGGATGCCCTTGCCCTGAG-TGGAGTCT
orangutan1 ATGACATCCA**-**CCTGGGCTTTCCTGGCCCCCAGAGAAGAGCTGTCCAGTGCATGTGCTGGCTGCACCGCGACGTCTCCTATGAACAAGGCCCACAGGCAGTGGCTGGCTGAGTGGACACCCCTGCCCTGAGCTGGAGTCT
orangutan2 ATGACATCCA**-**CCTGGGCTTTCCTGGCCCCCAGAGAAGAGCTGTCCAGTGCATGTGCTGGCTGCACCGCGACGTCTCCTATGAACAAGGCCCACAGGCAGTGGCTGGCTGAGTGGACACCCCTGCCCTGAGCTGGAGTCT

human CCCCAGGTGGCTGCTTCTTCCCCACCCCGAGGGTCACAGCCCAGCCCAAGCTCCAAGGGAATGGAGCAGCTGCCTGCACCAAGCTTCCCTCCTCTGGAGAAAGCACCTCCTCTTCTCCCTCACGTGCTCCCTGATGACCG
chimpanzee CCCCAGATGGCTGCTTCTTCCCCACCCCGAGGGTCACAGCCCAGCCCAAGCTCCAAGGGAATGGAGCAGCTGCCTGCACCAAGCTTCCCTCCTCTGGAGAAAGTGCCTCCTCTTCTCCCTCACGTGCTCCCTGATGACCG
orangutan1 CCCCAGGTGGCTGCTTCTTCCCCACCCCGAGGGTCACAGCCCAGCCCAAGCTCCAAGGGAATCGAGCAACTGCCTGCACCAAGCTTCCCTCCTCTGGAGAAGGCACCTCCTCTTCTCCCTCATGTGCTCCCTGATGACCG
orangutan2 CCCCAGGTAACTGCTTCTTCCCCACCCCGAGGGTCACAGCCCAGCCCAAGCTCCAAGGGAATCGAGCAGCTGCCTGCACCAAGCTTCCCTCCTCTGGAGAAGGCACCTCCTCTTCTCCCTCATGTGCTCCCTGATGACCG

human CCAGGCTCCTTGACATGGTTCCCAGGACCAGCCTCTCCCAGGGTCCCTACTCCCAGTACCCCAGTCCTCACCATTTGTGA
chimpanzee CCAGGCTCCTTGACATGGTTCCCAAGACCAGCCTCTCCCAGGGTCCCTACTCCCAATACCCCAGTCCTCACCATTTATGA
orangutan1 CCATTCTCCTTGACATGGTTCCCAGGACCAGCCTCTCCCAGGGTCCTGAATCCCAGTACCCCAGTCCTCACCATTTGTGA
orangutan2 CCATTCTCCTTGACATGGTTCCCAGGACCAGCCTCTCCCGGGGTCCTGAATCCCAGTACCCCAGTCCTCACCATTTGTGA

**ENSG00000205148**
human ATGATTTCATTCCATGTAATATTTTTGTCATTAGGAAGAGGCAAGTTATTTTTACCTGTCAATTTCTGTTTTTTGAAATTGAAAAATTCTCAGGTA**CGA**ATCCCAAAAGACTTTACATGCAACCTCCATGTCCTATTCAG
chimpanzee ATAATTTCATTCCATGTAATATTTTTGTCATCAGGAAGAGGCAAGTTATTTTTACCTGTCAATTTCTGTTTTTTGAAATTGAAAAATTCTCAGGTA**TGA**ATCCCAAAAGACTTTACATGCAACCTCCATGTCCTATTCAG
orangutan ATGATTTCATTCCATGTAATATTTTCCTCTTTAGGAAGAGGGAAGTTATTTTTACCTGTCAATTTCTGTTTTCTGAAATTTAAAAATTCTCAGGTA**TGA**ATCCCAAAAGACTTTACACGCAACCTCCATCTCCTATTCAG

human GACTGTTCAAGGAGAAGACAGAAACACCATGTTTAGAGGTCATTGGTCCATCTACTCTAGAAACTTCCCCTTGGCAGTGGTCCCTGCCTATGTTACAGAAGACGGCAAAAATACGAGCCATCGTGCATCTGGCCAGTTCT
chimpanzee GACTGTTCAAGGAGAAGACAGAAACACCATGTTTAGAGGTCATTGGTCCATCTACTCTAGAAACTTCCCCTTGGCAGTGGTCCCTGCCTATGTTACAGAAGACGGCAAAAATACGAGCCATCGTGCATCTGGCCAGTTCT
orangutan GACTGTTCAAGGAGAAGACAGAAACACCATGTTTAGAGGTCATTGGTCCATCTACTCTAGAAACTTCCCCTTGGCAGTGGTCCCTGCCTATGTTACAGAAGACGGCAAAAATACGAGCCATCGTGCATCTGGCCAGTTCT

human GCAATGCTCTATCCCAGGGGGAAATTCCTTCCTCACTCCAGCTGGTGAACAGCTATGCCCTGGAGCCCAGGACGGACATGCCTTGTAACTTTTTAACTTAG
chimpanzee GCAATGCTCCATCCCAGGGGGAAATTCCTTCCTCACTCCAGCTGGTGAACAGCTATGCCCTGGAGCCCAGGACGGACATGCCTTGTAACTTTTTAACTTAG
orangutan GCAATGCTCCATCCCAGGGGGAAATTCCTTCCTCACTCCAGCTGGTGAACAGCTATGCCCTGGAGCCCAGGACGGACATGCCTTGTAACTTTTTAACTTAG

**ENSG00000196273**
human ATGGGCATAGGTACTGGGCACACCTCGATGAACAAGGGAGGTAAGGATGTGACTCTCCTAGAACTTTCTGTC**GAG**AAGAGGAGATGGAGAATAAACATGGAAACAAGCAAGATCATTCTGGAGAAAATGCAATCAGATGA
chimpanzee AGGGGCATAGGTACTGGGCATACCTCGATGAACAAGGGAGGTAAGGATGTGACTCTCCTAGACCTTTCTGTC**TAG**AAGAGGAGATGGAGAATAAACATGGAAACAAGCAAGATCATTCTGGAGAAAATGCAATCAGATGA
orangutan ATGGGCATAGGTACTGGGCACACCTTGATGAACAAGGGAGGTAAAGATGTGACTCTCCTAGACCTTTCTGTC**TAG**AAGAGGAGATGGAGAATAAACATGGAAACAAGCAAGATCATTCTGGAGAAAATGCAATCAGATGA

human TGTGCTAGATGGTAACAGAGAGAGGTCGAATGAGAGAGAGGGCCGTGACAGCCTCTCTGAGAAGCTGAAATCCAAGCAGAACCTGAAAGACGAGGAGAAACTCAGATACATAAAGACTGGGAAAAGCATTCAAGTAGAGG
chimpanzee TGTGCTAGATGGTAACAGAGAGAGGTCGAATGAGAGAGAGGGCCGTGACAGCCTCTCTGAGAAGCTGAAATCCAAGCAGAACCTGAAAGACGAGGAGAAACTCAGATACATAAAGACTGGGAAAAGCATTCAAGTAGAGG
orangutan TGTGCTAGATGGTAACAGAGAGAGGCCGCATGAGAGAGAGGGCCGTGACAGCCTCTCTGAGAAGCTGAAATCCAAGCAGAACCCGAAAGACGAGGAGAAACTCAGATACATAAAGACTGGGAAAAGCATTCAAGTAGAGG

human GAACAGTGCGTGCAAAGGCCCTGAGGTGGGTGCAGTAG
chimpanzee GAACAGTGCGTGCAAAGGCCCTGAGGTGGGTGCAGTAG
orangutan GAACAGTGTGTGCAAAGGCCCTGAGGTGGGTGCAATAG

ENSG00000214707

human ATGCATTACG GAGCAGCAAC CCACATACAG AACTCGAGAA GCCACGGCCT GGAGACTGTA CCTGGACACC AAAGACTGGA GAGAGGAGCT GGTGGGGAAA CCCCAGAGTT CCCAGGGTGC CACTCCCCAG CTCCACCAGA

chimpanzee ATGCATTACG GAGCAGCAAC CCACATACAG AACTCGAGAA GCCACGGCCT GGAGACTGTA CCTGGACACC AAAGACTGGA GAGAGGAGCT GGTGGGGAAA CCCCAGAGTC CCCAGGGTGC CACTCCCCAG CTCCACCAGA

orangutan ATGCATCACG GAGCAGCAAC CCACATACAG AACTCAAGAA GCCACGACAT GGAGACTGTA CCTGGACACC AAAGACTGGA GAGAGGAGCT GGTGGGGAAA CCC--GAGTC CCCAGGGTGC CACTCCCCAG CTCCACCAGA

human GAACTTTGGG AATGAGCTGC TGCCCCTGAG TGCCCCTCTC CAGGGCCTCA GTGAGGGTCT CTACCCTCCA GGGAGGAACA AAACCTTGCC AGCTGGGGTC CTGCGAGAGG GGGCAGTTCA ATTCCTCCAC AGGGGACTCT

chimpanzee GAACATTGGG AATGAGCTGC TGGCCCTGAG TGCCCCTCTC CAGGGCCTCA GTGAGGGTCT CTCCCCTCCA GGGAGGAACA AACCCTTGCC AGCTGGGGTC CTGCGAGAGG GGGCAGTTCA ATTCCTCCAC AGGGGACTCT

orangutan GAACTTTGGG AATGAGCTGC TGCCCCTGAG TGCCCCTCTC CAGGGCCTCA GTCAGGATCT CTCCCCTCCA GGGAGGAACA AACCCTTGCC AGCTGGGGTC CTGTGAGAGG GGGCAGTTCA ATTCCTCCAC AGGGAACTCT

human GCAACTCCAA TCTTTCGAGT GAAGCATCTG CGAGGCCCTC AGGGACCCAG GATGAACTGC ATAGCAGC**AG A**AGGAAGACA GGCCAGACCA GGCGGGAGGG AGCCCGGAAA CATCTGGTTT GTAGTTTCAG ACTCTACCCG

chimpanzee GCAACTCCGA TCTTTCGAGT GAAGCATCTG CGAGGCCCTC AGGGACCCAG GATGAACTGC ATAGCAGC**TG A**AGGAAGACA GGCCAGACCA GGCGGGAGGG AGCCAGGAAA CATCTGGTTT GTAGTTTCAG ACTCTACCCG

orangutan GCAACTCCAA TCTTTGGAGT GAAGCATCTG CGAGGCCCTC AGGGACTCAG GATGAACTGC ATAGCAGC**TG A**AGGAAGACA GGCCAGACCA GGCGGGAGGG AGCCAGGAAA CATCTGGTTT GTAGTTTCAG ACTCTACCCG

human TTCACAGTTC ACACAGTCTC ACCGGGAAAC TCACACCTTG CCCT------ -GTACCAAGT TTTTAAGGCA GTTAAGCTCT GCCCATCCGA GACTTCATTT TTCTTGAGTA GA---AAATC ACTGAAATCA TCAGATCCAT

chimpanzee TTCACAGTTC ACACAGTCTC CCCGGGAAAC TCACACCTTG CCCTTAAACT TGTACCAAGT TTTTAAGGCA GTTAAGCTCT GCCCATCCGA GACTTCATTT TTCTTGACTA GAAGAAAATC ACTGAAATCA TCAGATCCAT

orangutan TTCACAGTTC ACACAGTCTC CCCGGGAAAC TCACACCTTG CCCT------ -GTACCAAGT TTTTAAGGCA GTTAAGCTCT GCCCATCCGA GACTTCATTT TTCTTGAGAA GA---AAATC ACTGAAATCA TCAGATCCAT

human GGCACCCACC TTCACTTTCT CCTAACAGCT GGAACCGTCA GGCTGGCTTC AGGGCCTGGT CTTCGCACTT GATATCACTA TCCCTCACCT GCTCAGACAG CCAGAGCAGG CGGGTGAGTT CCTCCCAACA ACCTCCACTG

chimpanzee GGCACCCACC TTCACTTTCT CCTAACAGCT GGAACCGTCA GGCTGGCTTC AGGGCCTGGT CTTCGCACTT GATATCACTA TCCCTCACCT GCTCAGACAG CCAGAGCAGG CGGGTGAGTT CCTCCCAACA ACCTCCACTG

orangutan GGCACCCACC CTCACTTTCT CCTAACAGCT GGAACTGTCA GGCTGGCTTC AGGGCCTGGT CTTCGCACTT GATATCACTA TCCCTCACCT GCTCAGACAG CCGGAGCAGG CAGGTGAGTT CCTCCCAACA ACCTCCACTG

human CATTCTCTCA GCTCCCACCG CAGGGCAGCC CACGTGCCTG AGTGA

chimpanzee CATTCTCTCA GCTCCCACCA CAGGGCAGCC CACGTGCCTG AGTGA

orangutan CATTCTCTCA GCTCCCACCG CAGGGCAGCC CACGTGCCTG AGTGA

ENSG00000224377

human ATGGAGAAGT TACGGAAGTG GGTGCTTTGG GACGTTCGGT ACCCCTCGGC GGCGTGGTCA GGTGGGGAAC ATGGCCGCGC CCACGTCGCG CTTCCCCATG GGATACACCA TGTTGGAGGA GTGTCCATCC GTATTGAGGA

chimpanzee ATGGAGAAGT TACGGAAGTG GGTGCTTTGG GACGTTCGGT GCCCCTCGGC GGCGTGGTCA GGTGGGGAAC ATGGCCGCGC CTACGTCGCG CTTCCCCATG GGGTACACCA TGTTGGAGGA GTGTCCATCC GTATTGAGGA

orangutan ATGGAGAAGT TACGGAAGCG GGTGCTTTGG GACGTTCGGT GACCCTCGGC GGCGTGGTCA GGTGGGGAAC ATGGCCGCGC CCACGTCGCG CTTCCCCATG GGGTACACCA TGTTGGAGGA GTGTCCATCC GTATTGAGGA

human TGAGGACACC TCTGAACTCA CAGAGCAGGC TGTGAGTTTA GAGCTGTCTG CTCTAAACTC AGGTGGAGCT GTCCCGCTGG TGGCAGGGAG CACGGGTGGG GACGTTTCTC TGGGCGGGCT AACTGGAGGA GGGG**G**TCTTT

chimpanzee TGAGGACACC TCTGAACTCA CAGAGCAGGC TGTGAGTTTA GAGCTGTCTG CTCTAAACTC AGGTGGAGCT GTCCCGCTGG TGGCAGGGAG CACGGGTGGG GACGTTTCTC TGGGCGGGCT AACTGGAGGA GGGG**-**TCTTT

orangutan TGAGGACACC TCTAAACTCA CAGAGCAGGC TGTGAATTTG GAGCTGTCTT CTCTAAACTC AGGTGGAGCT GTCCCGCTGG TGGCGGGGAG CACGGGTAGG GACGTTTCTC TGGGCGGGCT AACTGGAGGA GGGG**-**TCTTT

human GGACCTGTAG TCTATGGATC TGTGTGATAC CGCTCCGAGA AACTGAAGGG CCTCTTGGAA AGAATCAGGG GCGGCCGCGG CTATCTCCTG GCAGATACCA CAGGATGAGA AGCGGGGAGA TGTCAGGCTG GTTACAGGCT

chimpanzee GGACCTGTAG TCTATGGATC TGTGTGATAC CGCTCCGAGA AACTGAAGGG CCTCTTGGAA AGAGTCAGGG GCGGCCGCGG CTATCTCCTG GCAGATACCA CAGGATGAGA AGCGGGGAGA TGTCAG-CTG GTTACAGGCT

orangutan GGACCTGTCG TCTATGGATC TGTGTGATAC CGCTCCGAGA AACTGAAGGG CCTCTTGGAA AGAATCAGGG GCGGCCGCGG CTATCTCCTG GCAGATACCA CAGGATGAGA AGCGGGGAGA TGTCAGGCTG GTTACAGGCT

human TCTCCTAGGA CATACCACAA GGG---GAGG AGGAGGAGGA GAAATCATGG TTATGCAAGC CCTCCTAGGA TATGCCATGA GGTGGGAAAA AGGGAGAACT CAGAGTGCTG GGTTCCCCTC CTGCTGTGCC TGTGGGGCTT

chimpanzee TCTCCTAGGA CATACCACAA GGGAAGGAGG AGGAGGAGGA GAAATCATGG TTATGCAAGC CCTCTTAGAA TATGCCATGA GGTGGGAAAA AGGGAGAACT CAGAGTGCTG GGTTCCCCTC CTGCTGTGCC TGTGGGGCTT

orangutan TCTCCTAGGA CATACCACAA GGG------A AGGAGGAGGA GAAATCAGGG TTATGCAAGT CCTCCTAGGA TATGCCATGA GGTATGAAAA AGGGAGAACT CAGAGTGCGG GGTTCCCCTC CTGCTGTGCC TGTGGGGTTT

human GGATGCGAGG -TTTGAATGC TGCAGCAATT TACCTTATCT CCCACTCCAT CTACCCCAGC TAAAAAGGGA CTACGTAGTA CCAATACTAA TAATTTTTAA TGTGTCCTTA GTCCAATGCA ATATGTCAAT AAAAATCTTT

chimpanzee GGATGCGGGG -TTTGAATGC TGCAGCAATT TACCTTATCT CCCACTCCAT CTACCCCAGC TAAAAAGGGA CTACGTAGTA CCAATACTAA TAATTTTTAA TGTGTCCTTA GTCCAATGCA ATATGTCAAT AAAAATCTTT

orangutan GGATGCGGGG GTTTGAATGC TCC---AATC TACCTTATCT CCCACTCCAT CTACCCCAGC TAAAAAGGGA CTATGTAGTA CCAATACTAA TAATTTTTAA TGTGTCCTTA GTCCAATGCA ATATGTCAAT TAAAATCTTT

human AGGAAACTTT ATTATTTTTA TTTCCTCATG AAGTAA

chimpanzee AGGAAACTTT ATTATTTTTA TTTCCTCATG AAGTAA

orangutan AGGAAACTTT ATTATTTTTA TTTCCTCATG AAGTAA

ENSG00000237858

human ATGCTCCGGC AGTCCTGCTC CTTCCCGGTT ACGTCCCTTC CAGCCCTTGG CGGAGTCTGC GGGCGAGAGG GCGCAGGTGC AGAGGTGCCG CCGGCGGCGT GCGGCTGCGA AGGAAGGGAC CCCGACACCG AGCGTTCCTG

chimpanzee ATGCTCCGGC AGTCCTGTTC CTTCCCGGTC ACGTCCCTAC AAGCCCTTGG CGGAATCTGC GGGCGAGAGG GCGCAGGTGC AGAGGTGCCG CCGGCGGCGT GCGGCTGCGA AGGAAGG-AC CCCAACACCG AGCGTTCCTG

orangutan ATGCTCCGGC AGTCCTGTTC CTTCCTGGTT ACGTCCGTAC AAGCCCTTGG CGGAATCTGC GGGTGAGAGG GCGCAGGTGC AGAGGTGCCG CCGGCGGCGT GCGGCTGCGA AGGAAGGGAC CTCAACACCC AGCGTTCCTG

human CGGACGCTCC TCAACCGGGG GTTGTTCTCC GTGCTCTGGA CCCGGCCCTT CC**TCA**CCTAG GACGTCTCGC GGTGCACTGT CCCCCTCTCT CGGGCGACTT TTCCCCCACC TCCAAGTAGT GATCAAGCTC AGAATCCAGC

chimpanzee CGGACGCTCC TCAACCGGGG GTTGTTCTCC GTGCTCTGGA CCCGGCCCTT CC**TGA**CCTAG GACGTCTCGC CGCGCACTGT CCCCCTCTCT CGGGCGACTT TTCCCCCATC TCCAAGTAGT GATCGAGCTC AGAATCCAGC

orangutan CGGACGCTCC TCAGCCGGGG GTTGTTCTAC GTGCTCTGGA CCCCGCCCTT CC**TGA**CCTAG GATGTCTCGC CGCGCACTGT CCCCCTCTCT CGGGCGACTT TTCCCCCACC TCTAAGTAGT GACCAAGCTC AGAATCCAGC

human TCGCGCCCGC TGTGCACCTA GCACTTCCCA CCTGTAGTCT TTTGACCTCA TCGCCCCCTC TAGAAGGCTG CTGCCATCGC TTAAATGAGG AAGCCGAGGT GCAGAGAGGT TTCAGACCTA TTGCGGTGGA GCTGGAGTTC

chimpanzee TCGCGCCCGC TGTGCACCTA GCACTTCCCA CCTGTAGTCT TTTGACCTCA TCGCCCCCTC TAGAAGGCTG CTGCCATCGC TTAAATGAGG AAGCCGAGGT GCAGAGAGGT TTCAGACCTA TTGCGGTGGA GCTGGAATTC

orangutan TCGCGCCTGC TGTGCACCTA GCACTTCCCA CCTGTAGTCT TTTGACCTCA TCGCCACCTC GAGAAGGCTG CTGGCATCGC TTAAATGAGG AAGCCGAGGC GCAGAGAGGT TTCAGACCTA TTCCGGTGGA GCTGGAATTC

human GAGAACCAAC CACTCGGTGC TGAGACGCGC TTGCGGAACG GGCGGCGGGC GGGGGTGAAG CGGAGTGAGG GGAGAGGCCA GGTGCGACCC GGGCAGGTGA GGAGCACGGG CCCGGAGGGA GGACTTACGA GAATGGAAAG

chimpanzee GAGAACCAAC CACTCGGTGC TGAGACGCGC TTGCGGAACG GGCGGCGGGC GGGGGTGAAG CGGAGTGAGG GGAGAGGCCA GGTGCGACCC GGGCAGGTGA GGAGCACGGG CCCGGAGGGA GGACTTACGA GAATGGAAAG

orangutan GAGAACAAAC CGCTCGGTGC TGAGACGCGC GTGCTGAACG GGCGGCGGGC GGGGGTGAAA CGGAGTGAGG GGAGAGGCCA GATGCGACCC GGGAAGGTGA GGAGCACGGG CCCGGAGGGA GGACTTACGA GAATGGAAAG

human GAAAGCAGCC CGTCTTCAGT GGGACAGTGG GAGCATTAAG ATGTCAGAGA ATGAGCAGTT GTGGGAAGAG CCATAG

chimpanzee GAAAGCAGCC CGTCTTCAGT GGGACAGTGG GAGCATTAAG ATGTCAGAGA ATGAGCAGTT GTGGGAAGAG CCATAG

orangutan GAAAGCAGTC TGTCTTCAGT GGGACAGTGG GAGCATTAAG ATGTCAGAGA ATGAGCAGTT GTGGGAAGAG CCATAG

ENSG00000212929

**human**  ATGGAGGCCC CCCAC----- ----CCATTC CACTCCGGGG TTGCGGCCAC GCACCATAAG AGCACCTTCA GGTCTGAGCT CTTTAGGGGT GGGAGTAGGC AGTTCGTGAG TCCGGGAAGG CCTGCGGGGT TTCCCGCCTG

**chimpanzee** ATGGAGGCCC CCCGCCCCCC CCCCCCATTC CGCTCCGGGG TTGCGGCCAC GCACCATAAG AGCACCTTCA GGTCTGAGCT TTTTAGGGGT GGGAATAGGC AGTTCGTGAG TCCGGGAAGG CCTGCGGGGT TTCCCGCCTG

**orangutan**  ATGGAGGCCC CC-------- ----CCATTC CGCTCCGGGG TTGCGGCCAC GCACCATAAG AGGACCTTCA GGTCTGAACT CTTTAGGGGT GGGAGTAGGC AGTTCGTGAG TCCGGGAAGG CCTGCGGGGT TTCCCGCCTG

**human**  CTGCGGACTT AGCGTGGGGC CGACCGGGGC TG**GCGAGGGC TG**GCGAGGAC TGGCGGGGAC CCGCGGGGCT GAGCCAGCTC TCGCGAAGCC CTCAAGTGAG GAACGGCGCT TGTGGCTGCG CGCTCTCCGC AGCCAAGTTG

**chimpanzee** CTGCGGACTT AGCGTGGGGC CGAC-GGGGC TG**-------- --**GCGAGGAC TGGCGGGGAC CCGCGGGGCT GAGCCAGCTC TCGCGAAGCC CTCAAGTGAG GAACGACGCT TGTGGCTGCG CGCTCTCCGC AGCCAAGTTC

**orangutan**  CTGCGGACTT AGCGTGGGGC CGACCGGGGC TG**-------- --**GCGAGGAC TGGCGGGGAC CCGCGGGGCT GAGCCAGCTC TCGCGAAGCC C---AGTGAG GAACGACGCT TGTGGCTGCG CGCTCTCCGC AGCCAAGTTG

**human**  CAGGGTCCAG CAGGGGCTCA GGTCCTGTTC CCTCCGCAGA TCCCGGATCT AGGGCTCTAG TGGTCTCGGC CGGAGGGAAG GTGACGCGCA GTGGGCGCAG ACGCAGAGTG CGGGGCGCCG AACGTGGGAA GGAGCGGGTT

**chimpanzee** CAGGGTCCAG CAGGGGCTCA GGTCCTGTTC CCTCCGCAGA TCCCGGATCT AGGGCTCTAG AGGTCTCGGC CGGAGGGAAG GTGACGCGCA GTGGGCGCAG ACGCAGAGTG CGGGGCGCCG AACGTGGGAA GGAGGGGGTT

**orangutan**  CAGGGTCCAG CAGGGGCTCA GGTCCTGTTC CCTCCGCATA TCCCTGATCT AGGGCTCTAG TGGTGTCGGC CGGAGAGAAG GTGACGCGCA GTGGGCGCAG ACGCAGAGTG CCGGGCGCCG AATGTGGGAA GCAGCGGGTT

**human**  CAGCGCGCTG GTGAGAGTTT CAGGAAATCC GGGAGAGGGC GGTATTTACC AGTCCCTTCC CCGAGAGCAA CCAGGCAAAT CGGGGAAGGT TAG

**chimpanzee** CAGCGCGCTG GTGAGAGTTT CAGGAAATCC GGGAGAGGGC GATATTTACC AGTCCCTTCC CCGAGAGCAA CCAGGCAAAT CGGGGAAGGT TAG

**orangutan**  CAGCGCGCTG GTGAGAGTTT CAGGAAATCC GGGAGAGGGC GGTATTTACC AGTCCCTTCC CCGAGAGCAA CCAGGCAAAT CGGGGAAGGT TAG

ENSG00000236314

human ATGGGGGCTC AGTCGGGG**-**C CCCTTCC--- ACAGACAGAG CCGTGGCCCC TCAAGGTGGA CAGTGAGGCC GCTGCTCCTC CAGCTGCCTC TGTCTCATCA CAGGCCAGAA GTGGAAAGGG TTTCAAATCC AAGCCCCCGG

chimpanzee ATGGGGGCTC AGTCGGGG**G**C CCCTTCC--- ACAGACAGAG CCGTGGCCCC TCAAGGTGGA CAGTGAGGCC GCTGCTCCTC CAGCTGCCTC TGTCTCATCA CAGGCCAGAA GTGGAAAGGG TTTCAAATCC AAGCCCC-GG

orangutan ATGGGGGCTC AGTCGGGG**A**C CCTTCCACAG ACAGACAGAG CCCTAGCCCC TCAAGGTGGA TAGTGAGGCC GCTGCTCCTC CAGCTGCCTC TGTCTCATCA CAGGCCAGAA GTGGAAAGGG TTTCAAACCC AAGCCCC-AT

human GTTCCTGTGT GCGTTCTACG CAGCAAGGCT TCAAAGTTTC AAACTGGTTT GGAACTCAAC CAGGTCCTCC AAAGGAAACC AAGGCAGGCA CAGGATGGGG GCAGGCTGGC TTGGCAGGCA GCTGTGCGGA TCCACCCAGA

chimpanzee GTTCCTGTGT GTGTTCTACG CAGCAAGGCT TCAAAGTTTC AAACTGGTTT GGAACTCAAC CAGGTCCTCC AAAGGAAACC AAGGCAGGCA CAGGATGGGG GCAGGCTGGC TTGGCAGGCA GCTGTGCGGA TCCACCCAGA

orangutan GTTCCTGTGT GTGTTCTATG CAGCAAGGCT TAAAAGTTTC AAACTGGTTT GGAACTCAAC CAGGTCCTCC AAAGGAAACC AAGGCAGGCA CAGGATGGGG GCAGGCTGGC TTGGCAGGCA GCTGTGCCGA TCCACCCAGA

human GTGCCTTGGG AGGCAGTGGG GTTGGCTGTG GCTGCTGTGA GCTGCAGCCA GGCCTCTCCC GCGTCCCCAG GGTCTGGAAG GAGGGCCTCT GCACATTTTA TAAGCCACAG ACTGCGAAGG CCTGGAGCCC CACCTTCCAC

chimpanzee GTGCCTTGGG AGGCAGTGGG GTTGGCTGTG GCTGCTGTGA GCTGCAGCCA GGCCTCTCCC GCGTCCCCAG GGTCTGGAAG GAGGGCCTCT GCACATCTTA TAAGCCACGG ACTGCGAAGG CCTGGAGCCC CACCTTCCAC

orangutan GTGCCTTGGG AGGCAGTGGG GTTGGCTCTG GCTGCTGTGA GCTGCAGCCA GGCCTCGCCC GCGTCCCCAG GGTCTGGAAG GAGGGCTTCT GCAAATTTTA TAAGCCAGGG ACTGCGAAGG CCTGGAGCCT CACCTTCCAC

human CCATAACAAA GAAAGGGATA GTTTCAGAAC TGCTTTTATT TGGATAACTG ACTGA

chimpanzee CCATAACAAA GAAAGGGATA GTTTCAGAAC TGCTTTTATT TGGATAACTG ACTGA

orangutan CCATAACAAA GAAAGGGATG GTTTCAGAAC TGCTTTTATT TGGATAACTG ACTGA

ENSG00000237270

human ATGCAGTTGT TA**CAG**GTCAG GACTACAAAC ACGTTGAGGA GGCTGCCCGG GACCCCACTC ATGCCGTCAG CAGGAGCCGT GAGAGTCGTG GCGTCTGTTC AGTCAGGTCA CTCTTGGTGG AGGATGTGGG ATGGACCCAC

chimpanzee ATGCGGTCGT CA**TAG**GTCAC GACTACAAAC ACGTTGAGGA GGCTGCCCGG GACCCCACTC ATGCCGTCAG CGGGAGCCGT GTGAGTCGTG GCGTCTGTTC AGTCAGGTCA CTCTTGGTGG AGGATGTGGG ATGGACCCAC

orangutan ATGCAGTTGT TC**TAG**GTCAG GACTACAAAC ACGTTGAGGA GGCTGCCCGG GACCCCACTC ACGCCGTCAG CGGGAGCCCT ATGAGTCGTG GTGGCTGTTC AGTCAGGTCA CTCTTGGTGG AGGACGTGGG ATGGACCCAC

human AGGTGCCGTG GAGGCTGGCA GCTGTGCTGA GATGCTAAGG ACCTCAGCTC CAGGCACCAG TTGTCCAAAC CTGAGCTTCT TGTTGGGACA GGTCATTCCC CTGGCTCACA CCGGCTCTGT GGAAACGCTG CCCTCAGAGG

chimpanzee AGGTGCCGCG GAGGCTGGCA GCTGTGCTGA GATGCTAAGG ACCTCAGCTC CAGGCACCAG TCATCCAAAC CTGAGCTTCT TGTTGGGACA GGTCATTCCC -TGGCTCACA CCGGCTCTGT GGAAACGCTG CCCTCAGAGG

orangutan AGGTGCCACG GAGGCTGGCA GCTGTGCTGA GATGCTAAGG ACCTCAGCTC CAGGCACCAG TCGTCCAAAC CTGAGCTTCT TGTTGGGACA CATCATGCCC -TGGCTCACA CCGGCTCTGT GGAAATGCTG CCCTCAGAGG

human AGAGCTGGGG GTGTAG---G CAGCGGGCGT CTCCCCTTCC ACCCTCCACA GCCCCCATGG CAGTGTCAGC CTCTCACAGG GGAGG

chimpanzee AGAGCTGGGG GTGTAG---G CAGCGGGCGT CTCCCCTTCC ACCCTC-ACA GCCCCCATGG CAGTGTCAGC CTCTCACAGG GGAGG

orangutan AGAGCTGGGG GTGAAGAAGG CAGCCGGCGT CTCCCCTTCC ACCCTCCACA GCCCCCATGG CAGTGTCAGC CTCTCACGGG GGGGG

ENSG00000175913

human **ATG**CAGCCTT CCTGGACTCC CGCACCGGTA CAGCGCACTG CATGTAATAT TACAGCCTGG GGCGGCGAAT TTGGGAAGGA GGGAGAGGGT CGTTGTGAGC AGGTTGCACT CAGCTCTGGC CCTCCGGAGG GTGCACTTCA

chimpanzee **ACG**CAGCCTT CCTGGACTCC CGCACCGGTA CAGCGCACTG CATGTAATAT TACAGCCTGG GGCGGCGAAT TTGGGAAGGA GGGAGAGGGT CGTTGTGAGC AGGTTGCACT CAGCTCTGGC CCTCCGGAGG GTGCACTTCA

orangutan **ACG**CCACCTT CCTGGACTCC CGCACCGGTA CAGCGCACTG CATGTAATAT TACAGCCTGG GGCGGCGAAT TTGGGAAGGA GGGAGAGGGT CGTTGTGAGC AGGTTGCACT CAGCTCTGGC CCTCCGGAGG GTGCACTGCA

human CGCCTCCAGA GAAGGTCCAC AGCCTCCTGG TGCTGAAAAC CTACGTCCCT CCACTGGCGA AACATTTGTG CAGAGCGGAC GATGGGATGG GGGCTGGAGA GGAGCAATGA AAGGGAGGAG ACACAGGCAA GCCTCAACTC

chimpanzee CGCCTCCAGA GAAGGTCCAC AGCCTCCTGG TGCTGAAAAC CTACGTCCCT CCACTGGCGA AACATTTGTG CAGAGCGGAC GATGGGATGG GGGCTGGAGA GGAGCAATGA AAGGGAGGAG ACACAGGCAA GCCTCAACTC

orangutan CGCCTCCAGA GAAGGTCCAC AGCCTCCTGG TGCTGAAAAC CTACGTCCCT CCACTGGCGA AACATTTGTG CAGAGCGGAC GATGGGATAG GGGCTGGAGA GGAGCAATGA AAGGGAGGAG ACACAGGCAA GCCTCAACTC

human CCCCAACTCG TCCCGAATCG ATCTTCGTTC CAACCGCGCA GGATGGGGCC CAGATGGTTT GCAAAGCGCA CACTCGCACA ACGCAATACA CAGAACAAGA TTCCGTTGTT ACAGCGCGTG GTTTACTAGA TGCTAAAAGG

chimpanzee CCCCAACTCG TCCCGAATCG ATCTTCGTTC CAACCGCGCA GGATGGGGCC CAGATGGTTT GCAAAGCGCA CACTCGCACA ACGCA-TACA CAGAACAAGA TTCCGTTGTT ACAGCGCGTG GTTTACTAGA TGCTAAAAGG

orangutan CCTCAACTCG TCCCGAATCG ATCTTCGTTC CAACCGCGCA GGATGGGGCC CAGATGGTTT GCAAAGCGCA CACTCGCACA GCGCA-TACA CAGAACAAGA TTCCGTTGTT ACAGCGCGTG GTTTACTAGA TGCTAAAAGG

human GTGGGAGTAG CAGGAGGGTC TTAA

chimpanzee GTGGGAGTAG CAGGAGGGTC TTAA

orangutan GTGGGAGTAG CAGGAGGGTC TTAA

ENSG00000225021

human ATGAACTCGT TGACCGTCAT GAGGGTCTGG GACCATTCCT CATCTGTGTA CCTCGAACCA ACTTCCACTG GGACAGTTCG GCAGCCAGCG ATCTCCTGGA TATACTCCAA ACTAGCAGAA AGACACACAT TGGCATCGTC

chimpanzee ATGAACTCGT TGACCGTCAT GAGGGTCTGG GACCATTCCT CATCTGTGTA CCTCGAACCA ACTTCCACTG GGACAGTTCG GCAGCCAGCG ATCTCTTGGA TATACTCCAA ACTAGCAGAA AGACACACAT TGGCATCGTC

orangutan ATGAACTCGT TGACCGTCAT GAGTGTCTGG GACCATTCCT CATCTGTGTA CCTTGAACCA ACTTCCACTG GGACAGTTCG GCAGCCAGCG ATCTCTTGGA TATACTCCAA ACTAGCAGAA AGACACACAT TGGCATCGTC

human ACTGCTAATA TCTGTTGGGC ATAGTACTAA GTGTGCTTTG CATGTGGTCC CCTTTCATCC TCGGAAAACC TCACGTGCAG AGACAAGGAC TGTCATCCCC AGCTTCACTG ATGAGGAA**-**C CGGCATGGAG AGGTGGGGCT

chimpanzee ACTGCTAATA TCTGTTGGGC ATAGTACTAA GTGTGCTTTG CATGTGGTCC CCTTTCATCC TTGGAAAACC TCATGTGCAG AGACAAGGAC TGTCATCCCC AGCTTCACTG ATGAGGAA**A**C CGGCATGGAG AGGTGGGGCT

orangutan ACTGCTAATA TCTGTTGGGC ATAGTACTAA GTGTGCTTTG CATGTGGTCC CCTTTCATCC TCGGAAAACC TCATGTGCAG AGACAAGGAC TGTCATCCCC AGCTTCACTG ATGAGGAA**A**C TGGCATGGAG AGGCGGGGCT

human CCCTGCTCAA GGCTGTGTGG CTCATACACA GTGCTGTGCA GCTTTGCAGA CAGGGCCGGT AAGTCTGGTT GGCTGGGCTG CTCTAGTTCC CTGCTTGCCA ACAGCCTCCT CACTTCTTTC TTTGCTCATT TTGTCACTAA

chimpanzee CCCTGCTCAA GGCTGTGTGG CTCATACACA GTGCTGTGCA GCTTTGCAGA TAGGGCCGGT AAGTCTGGTT GGCTGGGCTG CTCTAGTTCC CTGCTTGCCA ACGGCCTCCT CACTTCTTTC TTTGCTCATT TTGCCACTAA

orangutan CCCTGTTCAA GGCTGTGTGG CTCATACACA GTGCTGTGCA GCTTTGCAGA TTGGGCCGGC CAGTCTGGTT GGCTGGGCTG CTCTAGTTCC CTGCTTGCCA ACGGCCTCCT CACTTCTTTC TTGGCTCATT TTGTCCCTAA

human GCTGAGAAAC CTTTGA

chimpanzee GCTGAGAAAC CTTTAA

orangutan GCTGAGAAAC CTTTGA

ENSG00000205066

human ATGGGGAAGG GGGAGGAAGG GAAGTATGAA TATTTACCTC TGACCAATAA TAGAGGTGCC CTGGTCCCCA AAGAAATTGA CTCCCGAATT CCCTCCAAGC CTTACCCATA TAGCTTTTGT TTGTTTGTTT **GTTT**TCAGAC

chimpanzee ATGGGGAAGG GGGAGGAAGG GAAGTATGAA TATTTACCTC TGACCAATAA TAGAGGTGCC CTGGTCCCCA AAGAAATTGA CTCCCGAATT CCCTCCAAGC CTTACCCATA TAGCTTTTGT TTGTTTGTTT **----**TGAGAC

orangutan ATGGGGAAGG GGGAGGAAGG GAAGTATGAA TATTTACCTC TGACCAATAA TAGAGGTGCC CTGGTCCCCA AAGAAACTGA CTCCCGAATT CCCTCCAAGC CTTACCCATA TAGCTTTTTT TTTTTTTTTT **----**TGAGAC

human AAGGTCTTGT TCTGTCCAGT GCCGTGGCGT GACCACAGCT CATGACAACT TTGATTTCCC CGGCCCAAGC GAGCCTCTCA TTCAGCCTCC TGAGTGGCTG GGACCACAGA CGCCACACAC AGCTAATTTT TATGTATTCG

chimpanzee AAGGTCTTGT TCTGTCCAGT GCCGTGGCAT GACCACAGCT CATGACAACT TTGATTTCCC CGGCCCAAGC GAGCCTCTCA TTCAGCCTCC CGAGTGGCTG GGACCACAGA CGCCACACAC AGCTAATTTT TATTTATTCG

orangutan AAGGTCTTGT TCTGTCCAGT ACCGTGGCGT GATCACAGCT CATGACAACT TTGATTTCCC CGGCCCAAGC GAGCCTTTCA TTCAGCCTCC TGAGTGGCTG GGACCACAGA CGCCACACAC AGCTAATTTT TATTTGTTCG

human TTTTTATTTT TTT-GCAGAG ACAGGGTCTC CCTATGTTGC CCAAGCTGGT CTTGAACTCC TGGGCTCAAG TGATCCTCCT GCTTCAGCCT CCCAAAGTGC TAGGATTATA G

chimpanzee TTTTTATTTT TTT-GCAGAG ACAGGGTCTC CCAGTGTTGC CCAAGCTGGT CTTGAACTCC TGGGCTCAAG TGATCCTCCT GCTTCAGCCT CCCAAAGTGC TAGGATTATA G

orangutan TTTTTATTTT TTTTGCAGAG ACAGGGTCTC CCTGTGTTGC CCAAGCTGGT CTTGAACTCC TGGGCTCAAG TGATCCTCCT GCTTCAGCCT CCCAAAGTGC TAGGATTACA G

ENSG00000227316

human **ATG**CCCTCTA CTGAAGGCTT TGGGGGACTG GATAGGCTAG TCCTGCCCCC ACCTCCCCAA GTATTACCCC TCCTAAGTCC TGCTAGGGGA CGTATTCAGT TCAGTGGAAG AAAGAGCACA GGCTCCTGGG TCACCCAGAT

chimpanzee **GTG**CCCTCTA CTGAAGGCTT TGGGGGACTG GATAGGCTAG TCCTGCCCCC ACCTCCCCAA GTATTACCCC TCCTAAGTCC TGCTAGGGAA CGTATTCAGT TCAGTGGAAG AAAGAGCACA GGCTCCTGGG TCACCCAGAT

orangutan **GTG**CCGTCTA CTGAAGGCTT TGGGGGACTG GATAGGCTAG TCCTGCCCCC ACCTCCCCAA GTATTACCCC TCCCAAGTCC TGCTAGGGGA CATATTCAGT TCAGTGGAAG AAAGAGCACA GGCTCCTGGG TCACCCAGAT

human GGAAAATCTA CTCCTGGCCC AGCCGCTGCC TGATGATTGT GAGATTCTGT GTGGGTGGCG ATCTCTCTTG CACCTCACTT TCCTCATTGG TTTTGCACCT CACTTTCCTC ATTGGTTTTG TAACCATCAC TGTCATCAGC

chimpanzee GGAAAATCTA CTCCTGGCCC AGCCGCTGCC TGAT---TGT GAGATTCTGT GTGGGTGGCG ATCTCTCTTG CACCTCACTT TCCTCATTGG TTTTGCACCT CACTTTCCTC ATTGGTTTTG CAACCATCAC TGTCATCAGC

orangutan GGAAAATCTA CTCCTGGCCC AGCCGCTGCC TGAT---TGT GAG------- -----TGGTG ATCTCTCTTG CACCTCACTT TCCTCATTGG TTTTGCACCT CACTT----- ---------- -------CAC TGTCATCAGC

human ATCCGCCTTC ACACCTCACA GAGGTGTTGC AAAGCTCAGG CGAGCTGGCA CATCAACTAG CACCCAGTAG GTGCTCAATG CGTGCAGAAG GAACTCACAG AGGATGA

chimpanzee ATCCGCCTTC ACACCTCACA GAGGTGTTGC ATAGCTCAGG CGAGCTGGCA CATCAACTAG CACCCAGTAG GTGCTCA-TG CGTGCAGAAG GAACTCACAG AGGATGA

orangutan ATCCGCCTTC ACACCTCATA GAGGTGTTGC AAAGCTCAGG CAAGCTGGCA CATCAACTAG CACCCAGTAG GTGCTCAATG CTTGCAGAAG GAACTCATAG AGAATGA
