# Supplementary material for: De Novo Origin of Human Protein-Coding Genes
Source: PLoS Genet. 2011 Nov 10;7(11):e1002379. doi: 10.1371/journal.pgen.1002379 (PMC3213175; doi:10.1371/journal.pgen.1002379)
Supplement: Dataset S3 — Protein evidence for the 27 de novo genes in Table S1. (DOC) [file pgen.1002379.s010.doc]

**ENSG00000176723**

PRIDE

1903: GGSVESVSLAPSSVAPDSTSGLR

8667: VCPFCCCSCGDSVNEKTSLSQR

8669: TCRGGSVESVSLAPSSVAPDSTSGLR; VCPFCCCSCGDSVNEK

8671: RVCPFCCCSCGDSVNEK

8672: DSREAVQAPGYPEPAR

**ENSG00000225917**

PRIDE

8670: MQPGCAVPQSGR ; SGPGMAAAGGGSAVEPRR

PeptideAtlas

PAp00005606: MQPGCAVPQSGR

PAp00377577: QPLATTVSLLCR

**ENSG00000206113**

PRIDE

3268: MGRCSWHPECVSGQALV

8653: EMGPPSPR; GLPVTWSQLPPR; GLPVTWSQLPPR

**ENSG00000232330**

PRIDE

8543: SSTTSAQAWLLAKSR;

8660: MIPPQNGVSRK; GPQPASLAPPR

8661: MIPPQNGVSRK;

8664: MIPPQNGVSRK;

8665: MIPPQNGVSRK;

8670: MIPPQNGVSRK;

8671: MIPPQNGVSRK

8672: MIPPQNGVSRK

**ENSG00000214780**

PRIDE

8538：LRLREGKGLVQSHTGTLWAGPL

**ENSG00000203393**

PRIDE

616: RDFENNSVFIVDSGGTCAGLLPGYIGWC

**ENSG00000225860**

PRIDE

8665: LQGLWLLDSRTCTNGLPGPSGLQPPTK

**ENSG00000221972**

PRIDE

638: KAFLAQSTLLESTLEGAPEWAAPHPEEQR

8667: CSIPSLSSSSTFSLFSSGCWNPR

**ENSG00000224013**

PRIDE

2026: LTGLMAGDR

8653: TQGTHLGLERR

8660: GGEPPGPPRESLVSLR

8662: GWAGRVSLGMGTASPGSR

8670: VSLGMGTASPGSRGGEPPGPPR

**ENSG00000227520**

PRIDE

1986: QGGAGAARCAAALGR

8665: CLPGAGRQGGAGAAR

8666: SKCLPGAGR

8667: TGRLVTLESPLQVPAPPGNPQPPR

10051: CAAALGR

**ENSG00000203863**

PRIDE

8661: DSLAESPFDLSTGRNAPCGGALGNLR

8664: DSLAESPFDLSTGRNAPCGGALGNLR

**ENSG00000235766**

PRIDE

21: TLPASPSAGTPLWGAGHVLGDAGESPLPSHPCPNR

**ENSG00000229811**

PRIDE

96: QARATVQTQLPMSTKK

8543: IIAQKEQGLLRNRGSVQPEGMHRAEED

8669: KLEMQLVLPCWPLSFSPPGLR

**ENSG00000223857**

PRIDE

8653: MSGGHSSPEPPR

**ENSG00000230294**

PRIDE

8653: AAQCMCWLHRDVPYER; MTSNLGSPGPQR

**ENSG00000205148**

PRIDE

8653: IPKDFTCNLHVLFR

8658: IPKDFTCNLHVLFR ; NFPLAVVPAYVTEDGK

8668: ASGQFCNALSQGEIPSSLQLVNSYALEPR

8671: IPKDFTCNLHVLFR

**ENSG00000230891**

PRIDE

8671: WHCRWDLK

**ENSG00000196273**

PRIDE

96: IILEKMQSDDVLDGNR

97: IILEKMQSDDVLDGNR

3651: IILEK

8660: MGIGTGHTSMNK

9330: SNEREGRDSLSEK

**ENSG00000214707**

8670 MHYGAATHIQNSR; KHLVCSFR

9299 VSLYHPTLGPSAFANVPPR

9306 LCPSETSFFLSR

10163 GQDAHLQLQLPR

10211 GSALLAFCLK

10213 GQDAHLQLQLPR

10214 GQDAHLQLQLPR

10257 GSALLAFCLK

10265 PEAEETDEWEGISQGQPPPGSG

**ENSG00000224377**

8531 VSLELSALNSGGAVPLVAGSTGGDVSLGGLTGGGGLWTCSL

10534 LSPGRYHR

**ENSG00000237858**

244 LRIQLAPAVHLALPTCSLLTSSPPLEGCCHR

8662 PGQVRSTGPEGGLTR

8670 SSTGGCSPCSGPGPSSPR; LFPHLQVVIKLR

10252 STGPEGGLTRMER

10253 EGAGAEVPPAACGCEGR

**ENSG00000212929**

8409 ALVVSAGGKVTRSGR

8668 ALRSQVAGSSR

**ENSG00000236314**

23 RPGAPPSTHNK

338 AGTGWGQAGLAGSCADPPRVPWEAVGLAVAAVSCSQASPASPGSGR

8657 VDSEAAAPPAASVSSQARSGK

8662 VDSEAAAPPAASVSSQARSGK

**ENSG00000237270**

9779 DTSPLGGQSWGSPQPSR

**ENSG00000175913**

8670 PESIFVPTAQDGAQMVCK; TACNITAWGGEFGK

10281 HRQASTPPTR

**ENSG00000225021**

8669 HTLASSLLISVGHSTK

**ENSG00000205066**

3269 KMPGAGSIL
