# Supplementary material for: De Novo Origin of Human Protein-Coding Genes
Source: PLoS Genet. 2011 Nov 10;7(11):e1002379. doi: 10.1371/journal.pgen.1002379 (PMC3213175; doi:10.1371/journal.pgen.1002379)
Supplement: Dataset S4 — Alignments of 33 de novo genes for human, chimpanzee, and orangutan sequences. (DOC) [file pgen.1002379.s011.doc]

ENSG00000187488

**human**  ATGTGTCCTC ATCTCAGGCC CGTGCCTGGG ACCCCGTGTC TGCCCAGGTG GGCAGCCTTG AGCCCAGGGG ACTCAGTGCC CTCCATGCCC TGGCTGGCAG

**chimpanzee** ATGTGTCCTC ATCTCAGGCC CGTGCCTGGG ACCCCGTGTC TGCCCAGGTG GGCAGCCTTG AGCCCAGGGG ACTCAGTGCC CTCCATGCCC TGGCTGGCAG

**orangutan**  ACGTGCCCTC ATCTCAG-CC CGTGCCTGGG ACCCCGTGTC TGCCCAGGTG GGCAGCCTTG AGCCCAGGGG ACTCAGTTCC CTCCATGCCC TGGCTGGCAG

**human**  AAACCCTCAA CAGCAGTCTG GGCACTGTGG GGCTCTCCCC GCCTCTCCTG CCTTGTTTGC CCCTCAGCGT GCCAGGCAGA CTGGGGGCAG GACAGCCGGA

**chimpanzee** AAACCCTCAA CAGCAGTCTG GGCACTGTGG GGCTCTCCCC GCCTCTCCTG CCTTGTTTGC CCCTCAGCGT GCCAGGCAGA CTGGGGGCAG GACAGCCGGA

**orangutan**  AAACCCCCAA CAGCAGTCTG GGCACTGTGG GGCTCTGCCC GCCTCTCCCG C--TGTTTGC CCCTCAGCGT GCCAGGCAGA CTGGGG-CAG GACAGCCGGA

**human**  AGCTGAGACC AAGGCTCCTC ACAGAAGGGC CCAGGAAGTC CCCGCCCTTG GGACAGCCTC CTCCGTAGCC CCTGCACGGC ACCAGTTCCC CGAGGGACGC

**chimpanzee** AGCTGAGACC AAGGCTCCTC ACAGAAGGGC CCAGGAAGTC CCCGCCCTTG GGACAGCCTC CTCCGTAGCC CCTGCACGGC ACCAGTTCCC CGAGGGACGC

**orangutan**  AGCTGAGGCC AAGGCTTCTC ACAGAAGGGC CCCGGAAGTC CCCGCCCTTG GGACAGCCTC CTCCGTAGCC CCTGCACGGC ACCAGTTCCC CAATGGACGC

**human**  AGCAGGCCGC CTCCCGCAGC GGCCGTGGGT CTGCACAGCC CAGCCCA**GCC CA**AGGCCCCC AGGAGCTGGG ACTCTGCTAC ACCCAGTGAA ATGCTGTGTC

**chimpanzee** AGCAGGCCGC CTCCCGCAGC GGCCGTGGGT CTGCACAGCA CGGCCCG**--- --**AGGCCCCC AGGAGCTGGG ACTCTGCTAC ACCCAGTGAA ATGCTGTGTC

**orangutan**  AGCAGACCGC CTCCCGCAGC GGCTGTGGGT CTGCACAGCA CGGCCCA**--- --**AGGCCCCC AGGAGCCGGG ACTCTGCTAC ACCCAGTGAA ATGCTGTGTC

**human**  CCTTCTCCCC CGTGCCCCTT GATGCCCCCT CCCCACAGTG CTCAGGAGAC CCGTGGGGCA CGGAACAGGA GGGTCTGGAC CCTGTGGCCC AGCCAAAGGC

**chimpanzee** CCTTCTCCCC C-TGCCCCTT GATGCCCCCT CCCCACAGTG CCCAGGAGAC CCACGGGGCA TGGAACAGGA GGGTCTGGAC CCTGTGGCCC AGCCAAAGGC

**orangutan**  CCTTCTCCCC C-TGCCCCTT GCTGCCCCCT CCCCACAGTG CCCAGGAGAC CCGCGGGGCA TGGAACAGGA GGGTCTGGAC CCTGTGGCCC GGCCA-----

**human**  TACCAGACAG CCACAACCAG CCCAGCCACC ATCCAGTGCC TGGGGCCTGG CCACTGGCTC TTCACAGTGG ACCCCAGCAC CTCGGGGTGG CAGAGGGACG

**chimpanzee** TACCAGACAG CCACAACCAG CCCAGCCACC ATCCAGTGCC TGGGGCCTGG CCACTGCCTC TTCACAGTGG ACCCCAGCAC CTCGGGGTGG CAGAGGGACG

**orangutan**  ---------- ---------- --------CC ATCCAGTGCC TGGGGCCTGA CCACTGGCTC TTCACAGTGG ACCCCAGCAC CTCAGGGTGG CAGAGGGATG

**human**  GCCCCCACGG CCCAGCAGAC ATGCGAGCTT CCAGAGTGCA ATCTATGTGA TGTCTTCCAA CGTTAA

**chimpanzee** GCCCCCACGG CCCAGCAGAC ATGCGAGCTT CCAGAGTGCA ACCTATGTGG TGTCTTCCAA CGTTAA

**orangutan**  GCCCCTATGG CCCGGCAGAC GCGCGAGCTT CCGGAGTGCA ATCCATGTGA TGTCTTCCAA CGTTAA

ENSG00000183853

**human**  ATGGGTGAGG GGACCTGGAG AGGTAAGGGG CCTGGAAATG GCCCTGACAG AGAACTTGTG CTGACCGGGA GAAGGTGGTG CGGAAGGGCA CCGCAGGCAT

**chimpanzee** ATGGGTGAGG GGACCTGGAG AGGTAAGGGG C-TGGAAATG GCCATGACAG AGAACTTGTG CTGACCGGGA GAAGGTGGTG CGGAAGGGCA CCGCAGGCAT

**orangutan**  ATGGGTGAGG GGACCTGGAA AGGTAAGGGG CCTGGAAATG GCCCTGACAG AGAACATGTG CTGACCGGGA GAAGGTGGCG CGGAAGGGCA CCGCAGGCAT

**human**  TACACAGGCC ATTTCTCCTC CTCCATGTGC ACGCACATCC AACACACACC TTCCATGTGA CTGCTTCTCA GCCACCACTT TCTGACCAAA GAGAACAGGC

**chimpanzee** TCCACGGGCC ATTTCTCCTC CTCCATGTGC ACGCACATCC AACACACACC TTCCATGTGA CCGCTTCTCA GCCACCACCT TCTGACCAAA GAGAACAGGC

**orangutan**  TCCACGGGTC ATTTCTCCTC CTCCATGTGC ACGCACATCC AACACACACC TTCCATGTGA CCGTTTCTCA GCCACCACCT TCTGACCAAA GAGAACAGGC

**human**  GCTCCAAGGA GAACCTGTAC CCCTGCCCCA GCCTCGCCTT CCTAAAAAGC AGTGTCTGGA GTTGGCTGTT TCCCGCCTCC CCAGCTGCAT GTCCAGCCCA

**chimpanzee** GCTCCAAGGA GAACCTGTAC CCCTGCCCCA GCCTCGCCTT CCTAAGAAGC AGTGTCTGGA GTTGGCTGTT TCTGGCCTCC CCAGCTGCGT GTCCAGCCCA

**orangutan**  GCTCCAAGGA GAACCTGTAC CCCTGCCCCA GCCTCGCCTT CCTAAGAAGC AGTGTCTGGA GTTGGCTGTT TCCCGCCTCC CCAACTGCAT GTCCAGCCCA

**human**  GTGGGAGACA GGGCCTCTGG TGCAGTTCCC AAAATGTTCC TCGCCCTTTC TCCGTGGTCA CCATTCATAA TTCATGTTCG CTTTAATGAG TTACATGCTT

**chimpanzee** GTGGGAGACA GGGCCTCTGG TGCAGTTCCC AAAATGTTCC TCGCCCTTTC TCCGTGGTCA CCATTCATAA TTCATGTTCG CTTTAATGAG TTACATGCTT

**orangutan**  ATGGGAGACA GGGCCTCTGG TGCGGTTCCC AAAATGTTCC TCGCCCTTTC TCCGTGGTCA CCATTCATAA TTCATGTTCG CTTTAATGAG TTACATGCTC

**human**  ATCAGCCACA GGCCATTACC ACCCTTATGG ATGGGTCTGG GGGGCGACAT TCCTGGCCAA CCCCTTG**-**TA GGAAGGACCA GATAATACCC AGGAAGCAAG

**chimpanzee** ATCAGCCACA GGCCATTACC ACCCTTACGG ATGGGTCTGG GGGGCGATAT TCCTGGCCAA CCCCTTG**C**TA GGAAGGACCA GATAATACCC AGGAAGCAAG

**orangutan**  ATCAGCCACA GGCCATTACC ACCCTTACGG ATGGGTCTGG GGTGCGACAT TCCTGGCCAA CCCCTTG**C**TA GGAAAGACCA GATAATACCC AGGAAGCAAG

**human**  TAGCTCTAAT TTTAACTTCA CAGGAAGTCT GTGCATCCTC CTTCATTTCA GCAGGGAAAA CTCCTGTGGA GTGGGCCCTA TCTGGGGCAT TTACAGGCTT

**chimpanzee** TAGCTCTAAT TTTAACTTTG CAGGAAGTCT CTGCATCCTC CTTCATTTCA GCAGGGAAAA CTCCTGTGGA GTGGGCCCTA TCTGGGGCAT TTACAGGCTT

**orangutan**  TAGCTCTAAT TTTAACCTCG CAGGAAGTCT CTGCATCCTC CTTCATTTCA GCAGGGAAAA CTCCTGTGGA GTGGGCCCTA TCTGGGGCAT TTACACGCTT

**human**  CCAGCTGTAT TCCATCCCTG GAAGCTGA

**chimpanzee** CCAGCTATAT TCCATCCCTG GAAGCTGA

**orangutan**  CCAGCTGTAT TCCATCCCTG GAAGCTGA

ENSG00000203862

**human**  ATGAACACTC TT**--**ATTTTT ACACATTTGT GTTCTTTAGA ACCAATTACT ATGAGTCAAA ATTGTGCATA TTTAGTTTTA GTAGATACAG TACTGCAAAA

**chimpanzee** ATGAACACTC TT**TT**ATTTTT ACACATTTGT GTTCTTTAGA ACCAATTACT ATGAGTCAAA ATTGTGCATA TTTAGTTTTA GTAGATACAG TACTGCAAAA

**orangutan**  ATGAACACTC TT**TT**ATTTTT ACACATTTGT GTTCTTTAGA ACCAATTACT GTGAGTCAAA AT-GTGCATA TTTAGTTTTA GTAGATACAG TACTGCAAAA

**human**  TATTCCTCTA AAAAAGCTTA ACAACAGTGC TAATTTCCCT ATACCGTCCC TACCTGTGGA TACTATCAGA CTTTTAACTT TTTTGCAGTT TAAAAAGACA

**chimpanzee** TATCCCTCTA AAAAAGCTTA ACAACAGTGC TAATTTCCCT ATACCGTCCC TACCTGTGGA TACTATCAGA CTTTTAACTT TTTTGCAGTT TAAAAAGACA

**orangutan**  TATCCCTCTA AAAAAGCTTA ACAACAGTGC TAATTTCCCT GTACCGTCCC TACCTGTGGA TACTATCAGA CTTTTAACTT TTT-GCAGTT TAAAAACACG

**human**  TTACACATGC ACTCATACAC ACAAAAGCAG TCTTTGCTTA AATGTCACCT TCTTAATGAG GTCATCATGA TTTTCTTCTT AAAACTGAAA TACCACCCCC

**chimpanzee** TTACACATGC ACTCATACAC ACAAAAGCAG TCTTTGCTTA AATGTCACCT TCTTAATGAG GTCATCATGA TTTTCTTCTT AAAACTGAAA TACCACCCCC

**orangutan**  TTACACATGC ACTCATACAC ACAAAAGCAG TCTTTGCTTA AATGTCACCT TCTTAATGAG GTCATCATGA TTTTCCTCTT AAAATTGAAA TACCACCCCC

**human**  CTCAGCATTC CTTAATCCCC TTTACGTTAT TTTCTATAGT TCTTACCACC TTCTGGCATG CTGTTGTTTC TCTCCTTATT CTAGACTGTA GGCTCCAAAA

**chimpanzee** CTCAGCATTC CTTAATCCCC TTTACGTTAT TTTCTATAGT TCTTACCACC TTCTGGCATG CTGTTGTTTC TCTCCTTATT CTAGACTGTA GGCTCCAAAA

**orangutan**  CTCGGCATTC CTTAATCCCC TTTGCATTAT TTTCTATAGT TCTTACCACC TTCTGGCATG CTGTTGTTTC TCTCCTTATT CTAGACTGTA GGCTCTAAAA

**human**  AGGCAAAAGT TTGGGTTTTT GTCCTGTTAA TTATGAACAA TGCTTGGCAG ATGATAAAAA AA----CTCA CATATTTATT GAATTAAATG GCCATGGCAA

**chimpanzee** AGGCAAAAGT TTGGGTTTTT GTCCTGTTAA TTATGAACAA TGCTTGGCAT ATGATAAAAA AAAAAACTCA CATATTTATT GAATTAAATG GCCATGGCAG

**orangutan**  AGGCAAAGGT TTGGGTTTTT GTCCTGTTAA TTATGAACAA TGCTTGGCAT ATGATAAAAA A-----CTCG CATATTTACT GAATTAAATG GCCTTGGCAG

**human**  TTGTAGAATA CACATGAATT TCAGCCATAG CTGTTATGTT TCTCCCTTGG CCCATCTGTC CCTGGTTTAA

**chimpanzee** TTGTAGAATA CACATGAATT TCAGCCATAG CTGTTATGTT TCTCCCTTGG CCCATCTGTC CCTGGTTTAA

**orangutan**  TTGTAGAATA CACATGAATT TCAGCCATAG CTGTTAAGTT TCTCCCTTGG CCCATCTGTC CCTGGTTTAA

ENSG00000198447

**human**  ATGAAGAGCA TTTCTTTCTC C**TTA**GGGAGT GGTGGGGAGG CTGCACCAGG AAGACAGAGG GCAAATCCTA GTGTGTGTGA GAGAGAGAGA AAAAAAGAGA

**chimpanzee** ATGAAGAGCA TTTCATTCTC CTAAGGGAGT GGTGGGGAGG CTGCACCAGG AAGACAGAGG GCAAATCCTA GTGTGTGTGA GAGAGAGAGA AAAAAAGAGA

**orangutan**  ATGAAGAGCA TTTCATTCTC C**TAA**GGGAGT GGTGGGGAGG CTGCACCAGG AAGACAGAGG GCAAATCCTA GTGTGTGTGA GAGAGAGAGA AAAAAAGAGA

**human**  GAGAGAGACA GACAGAGACA GAGAGACAGA GATACAGAGA CAGAAAGAGA GAGACAGACA GAGACAGAAA GAGAGAGACA GAGACAGAGA GACAGGAACA

**chimpanzee** GAGAGAGACA GACAGAGACA GAGAGACAGA GATACAGAGA CAGAAAGAGA GAGACAGACA GAGACAGAAA GAGAGAGACA GAGACAGACA GACAGGAACA

**orangutan**  GAGGGAGACA GACAGAGACA GAGAGACAGA GATACAGAGA CAGAAAGAGA GAGACAGACA GAGACAGAGA GA-------- ---------- --CAGGAACA

**human**  GAGAGAGGTA TTCTGGATAA GATGGCTTAG AGGAGGCAGA ACTAGCTTGC AGCTCCAGCT CCGATGGACA GAGCAGCGTG TGGAGACTCA CATCGTAAAC

**chimpanzee** GAGAGAGGTA TTCTGGATAA GATGGCTTAG AGGAGGCAGA ACTAGCTTGC AGCTCCAGCT CCGATGGACA GAGCAGCATG TGGAGACTCA CATCGTAAAC

**orangutan**  GAGAGAGGTA TTCTGGATAA GATAGCGGAG AGGAGGCAGG ACTAGCTTGC AGCTCCAGCT CCGACAGACA GAGCAGCATG TGGAGACTCA CATCGCAAAC

**human**  TTTTGCTCCA AGACCTACCG CAAGAACATA CTGGGAAAGC CGAGGGAATC CACAGACCCT TTGAAGGAAC T-GATGACTG CTGCAGGCTC CCTGAGATGC

**chimpanzee** TTTTGCTCCA AGACCTACCG CAAGAACATA CTGGGAAAGC TGAGGGAATC CACAGACTCT TCGAAGGAAC T-GATCACTG CTGCAGGCTC CCTGAGATGC

**orangutan**  TTTTGCTCCA AGACCTACTG CAGGAACATA CTGGGAAAGC CGAGGGAATC CACAGACCCT TTGAAGGAAC TAGATCACTG CTGTAGGCTC CCTGAGATGC

**human**  CGAAAACCTG TGAGTTGGCT TGCTTTCTCA ACATGGAGGC TTGTGGTCTG GGGCAAGTTC TCGGCCCTGA TCACCACCTG CCTGGAAATA GACTCGGTGC

**chimpanzee** CAAAAACCTG TGAGTCGGCT TGCTTTCTCA ACATGGAGGC TTGTGGTCTG GGGCAAGTTC TCGGCCCTGA TCACCACCTG CCTGGAAATA GACTTGGTGC

**orangutan**  CGAAAACCTG TGAGTCGGCT TGCTTTCTCA ACATGGAGGC TTGTGGCCTG GGGCAAGTTC TCGGCCCTGA TCACCAGCTG CCTGGAAATA GACTTGGTGC

**human**  TGTTGGGAGG GCACAGTGGG AGTGAGACCA GCCTCTAG

**chimpanzee** TGTTGGGAGG GCACAGTGGG AGTGAGACCA GCCTCTAG

**orangutan**  TATTGGGAGG GCACAGTGGG AGTGAGACCA GCCTCTAG

ENSG00000205965

**human**  ATGTGGTGGC ATTTGTGCAC TCACAGTGCT GTGCACCCAC CCACACCGTC TAGTTTCAAA AGGCATTCAT CTCCCCAGAA GAAACCTCCC GTCCTCATTA

**chimpanzee** ATGTGGTGGC ATTTGTGCAT TCACAGTGCT GTGCACCCAC CCACGCCGTC TGGTTTCAAA AGGCATTCAT CTCCCCAGAA GAAATCTCCC GTCCTCATTA

**orangutan**  ATGTGGTGGC ATTTGTGCAT TCACAGTGCT GTGCAACCAC CCACGCCATC TAGTTTCAAA ATGCATTCGT CTCCCCGGAA GAAACCTCCC GTCCTCATTA

**human**  AGCAGTTACC CCTCCTTGGT ATCCCCCAAG CCCCTCTCCT GGGGTCCGAA GAGGGACTTG CCAGTGAGCG GAGCTCTGAT AATAAGGAAT CAGGCACCCA

**chimpanzee** AGCAGTTACC CCTCCTTGGT ATCCCCCAAG CCCCTCTCCT GGGGTCCGAA GAGGGACTTG CCAGTGAGCG GAGCTCTGAT AATAAGGAAT CAGGCACCCA

**orangutan**  AGCAGTTACC CCTCCTTGGT ATCCCCCAAG CCCCTCTCCT GCGGTCCGGA GAGACACTTG CCAGTGGGCG GAGCTCTGAT AATAAGGAAT CAGGCACCCA

**human**  CTGCTGGTCC AGGCCTGGGT TGGTTTTCCA CCCAGCAGAG GTGGCAGAGC CAGGAGGGTC TGGGAGCGCT ACAGGGGAGC CCCATGCTTG CCGCCGGAGC

**chimpanzee** CTGCTGGTCC AGGCCTGGGT TGGTTTTCCA CCCAGCAGAG GTGGCAGAGC CAGGAGGGTC TGGGAGCGCT ACAGGGGAGC CCCATGCTTG CCGCCGGAGC

**orangutan**  CTGCTGGTCC AGGCCTGGGT TGGTTTTCCA CCCAGCAGAG GTGGCAGAGC CAGGAGGGTC TGGGAGCGCT ACAGGGGAGC CCCATGCTTG CCGCCGGAGC

**human**  CCTGCCCC**GC CCC**GAGCTTC CCCACCAGGG GGCAGCAGAG AGCTTTCCAG AACCCGCCGC GGGGCTGGAG GGAAGCAGTG GCTCAGAGCT GCTGACAAAC

**chimpanzee** CCTGCCCC**-- ---**GAGCTTC CCCACCAGGG GGCAGCAGAG AGCTTTCCAG AACCCGCCGT GGGGCTGGAG GGAAGCCGCG GCTCAGAGCT GCTGACAAAC

**orangutan**  CCTGCCCC**-- ---**GAGCCTC CCCACCAGGG GGCAGCAGAG AGCTTTCCAG AACCGGCCGC GGGGCTGGCG GGCAGCAGAG GCTCAGAGCT GCTGACAAAC

**human**  CTCATGTTGA CCCCAGACCG CTGTCTCTGT GGGTTGGGCT TGGGAATTGG AGAGGAGGCC GCATGATTGG AAACATGAAG ACGGCACGGC CTGGCTGGAG

**chimpanzee** CTCATGTTGA CCCCAGACCG CTGTCTCTGT GGGTTGGGCT TGGGAATTGG AGAGGAGGCC GCATGATTGG AAACATGAAG ACGGCATGGC CTGGCTGGAG

**orangutan**  CTCATGTTGA CCCCAGACCG CTGTCTCTGT GGGTTGGGCT TGGGAATTGG AGAGGAGGCC GCGTGATTGG AAACATGAAG ACGGCACGGC CTGGCTGGAG

**human**  CAGCGGGAAG CGTCGTCACG GTCACTGA

**chimpanzee** CAGCGGGAAG CGCCGTCACG GTCACTGA

**orangutan**  CAGCGGGAAG CGCCGTCACG GTTACTGA

ENSG00000184827

**human**  ATGGGAACCG GATTGGTTGC AGTGTGGCAC AGACCTGGAA CCTTCCTGAA AGAGGTTGGG GCAGGCAGTG ACTGTTCAGA CGTCCAATCT CTTTGGGACG

**chimpanzee** ATGGGAACCG GATTGGTTGC AGTGTGGCAC AGACCTGGAA CCTTCCTGAA AGAGGTTGGG GCAGGCAGTG ACTGTTCAGA CGTCCAATCT CTTTGGGACA

**orangutan**  ATGGGAATCG GATTGGTTGC AGTGTGGCAC AGACCTGGAA CCTTCCTGAA AGAGGTTGGG GCAGGCAGTG ACTGTTCAGA CGTCCAATCT CTTTGGGACA

**human**  CCTCTTCAGC GCTGTCTTCC CTGCCTCTGC CTTTAGGACG AGTCTCAAAC ACCAACAAAC GCAAGGCACG TCCCCCCTCT CAGGTCAGCT GCGAAGGCGT

**chimpanzee** CCTCTTCAGC GCTGTCTTCC CTGCCTCTGC CTTTAGGACG AGTCTCAAAC ACCAACAAAC GCAAGGCACG TCCCCCCTCT CAGGTCAGCT GCGAAGGCAT

**orangutan**  CCTCTTCAGC TCTGTCTTCC CTGCCTCTGC CTTTAGGACG AGTCTCAAAC ACCAACAAAC TCAAGGCACG TTCCCCCTCT CAGGTCAGCT GCGAAGGGAT

**human**  GGGAGCAAGA GAGAGCCTCT CCCTCCTTCT ACTTAGAGTT TTTGTTTGTT TGTTTGTTTG T**TTGT**TTCAA ATATTAATGG ACGATAAAAC AACACGGATT

**chimpanzee** GGGAGCAAGA GAGAGCCTCT CCCTCCTTCT ACTTAGAGTT TTTGTTTGTT TGTTTGTTTG T**----**TTCAA ATATTAATGG AAGATAAAAC AACACGGATT

**orangutan**  GGGAGCAAGA GAGAGCCTCT CCCTCCTTCT ACTTAGAGTT TTTGTTTGTT TGTTTGTTTG T**----**TTCAA ATATTAATGG ACGATAAAAC AACACGGATT

**human**  TGTCACCTCC TAGCGAAGAG GAAGCCACCG CCACCACACC CCTCAAGCAT GCCTGGGCAC TCCTGGGGAC AATCGGACGT GGCAGGAGTT GGCATGCCCA

**chimpanzee** TGTCACCTCC TAGCGAAGGG GAAGCCACCG CCACCACACC CCTCAAGCAT GCCCGGGCAC TCCTGGGGAC AATCGGACGT GGCAGGAGTT GGCATGCCCA

**orangutan**  CGTCACCTCC TAGCGAAGAG GAAGCCACCG CCACCACACC CCTCAAGCAT GCCCGGGCAC TCCTGGGGAC ATTCGGACGT GGCAGGAGTT GGCATGCCCA

**human**  AGATGAAGCT GGGAAGGATG ACCCAGAGTG GAAGGCGTGG GCTGGGGCTC CAGGTCTCAG CTCTCGCCAG CGTCGGACCA CACAGCTTTG CCAGCTTTGC

**chimpanzee** AGATGAAGCT GGGAAGGATG ACCCAGAGTG GAAGGCGTGG GCTGGGGCTC CAGGTCTCAG CTCTCGCCAG CGTCGGACCA CACAGCTTTG CCAGCTTTGC

**orangutan**  AGATGGAGCT GGGAAGGATG ACCCAGAGTG GAAGGCGTGG GCTGAGGCTC CAGGTCTCAG CTCTTGCCAG CGTCGGACCA CACAGCTTTA CCAGCTTTGC

**human**  ACCTTGCCTT TCCTTCTTGT GA

**chimpanzee** ACCTTGCCTT TCCTTCTTGT GA

**orangutan**  ACCTTGCCTT TCCTTCTTGT GA

ENSG00000188745

**human**  **ATG**AAAACAG GGCCCCTTCA GTGTGGAACC GTGGGAGACG CGGAACACAA CACAGGGTGG TGGATTCCTG GGCGGAGCTC TGCCTGCCCC ACGGGCCTGT

**chimpanzee** **ATT**AAAACAG GGCGCCTTCA GTGTGGAACC GTGGGAGACG CGGAACACAA CACAGGGTGG TGGATTCCTG GGCGGAGCTC TGCCTGCCCC ACGGGCCTGT

**orangutan**  **ATT**GAAACAG GGCCCCTTCA GTGTGGAACC GTGGGAGATG CAGAACACAA CGGAGGGTTG TGGATTCCTG TGCAGAGCTC TGCCTGCCCC ATGGGCCTGT

**human**  GGTCTGCTCT GTCTGCCGGG GCCCAGTTTA CCGAACTACT TGGTGGCCAG ATAGCCACCG TAGGGCGTGA GCGGCCGGCA CGAAAGCCCA GGGCCTCTGT

**chimpanzee** GGTCTGCTCT GTCTGCCGGG GCCCAGTTTA CCGAACTACT TGGTGGCCAG ACAGCCGCCG TAGGGCGTGA GCGGCCGGCA GGAAAGCCCA GGGCCTCTGT

**orangutan**  GGTCTGCTCT GTCTGCCGGG GCCCAGTTTA CTGAACTAGT TGGTGGCCAG ACAGCCACCG CAGGGTGTGA GCGGCCGGCA CAAAAGCCCG GGGCCTCTGT

**human**  CTCCCGGGAG CTGCTGGCGC GGCGCGGCTG GGACACGCCG TACTCGGTGC CACGTTCTTG CCCAGTTCCT CGCTGTGGTG AGCTGCTGGC TGTTAGGAAG

**chimpanzee** CTCCCGGGAG CTGCTGGCGC GGCGCGGCTG GGACACGCCG TACTCGGTGC CACGTTCTTG CCCAGTTCCT CACTGTGGTG AGCTGCTGGC TGTTAGGAGG

**orangutan**  CTCCCAGGAG CTGCTGGCGT GGTGCGGCTG GGACACGCCA TACTCGGTGC CACCTTCTTG CCCAGTTCCT CGCTGTGGTG AGCTGCTGGC TGTTAGGAAG

**human**  CCTTTCCCTC GGCTGCGGCC CTCCCACACG GCCCCCAGAC CCCTGACTGC AGGGTGGGCG GGCGTACCAG GGGAGGCACT CTGGCCGGCC CGCTTGGCAG

**chimpanzee** CCTTTCCCTC GGCTGCGGCC CTCCCACACG GCCCCCAGAC CCCTGACTGC AGGGTGGGCG GGCATACCAG GGGAGGCACT CTGGCCGGCC CACTTGGCAG

**orangutan**  CCTTTCCCTC AGCTGCGGCC CTCCCACGCG GCCCCCGGAC CCCTGACGGC AGGGTGGGCG GGCGTACCAG GTGAGGCACT CTGGCCGGTC CACTTGGCAG

**human**  CAGGGCCACA GCTCCTCCTC GCAGGGCTTT GTGGGATTTT AAAGGATGAA TCGTCTTGTT CTCCATGCTC CCCCTGCCGC CCATGCGCGC CATGCTCTGT

**chimpanzee** CAGGGCCACA GCTCCTCCTC GCAGGGCTTT GTGGGATTTT AAAGGATGAA TCATCTTGTT CTCCCTGCTC CCCCTGCCGC CCATGCACGC CATGCTCTGT

**orangutan**  CAGGGCCACG GCTCCTCCTC GCAGGGCTTT GTGGAATTTT AAAGGATGAA TCATCTTGTT CTCCATTCTC CCCCTGCCGC CCATGCGCGC CATGCTCTGT

**human**  GTGA

**chimpanzee** GTGA

**orangutan**  GTGA

ENSG00000206028

**human**  ATGGAGAGGA TTCTTCCCCA GCTTCCTTCC AGAG---GAC ACAAAAGCTC AGAGCTCCAC AGTCTAGAGT CTAGACCAAC AGGCCTCCAC ACTCACGTCC

**chimpanzee** ATGGAGAGGA TTCTTCCCCA GCTTCCTTCC AGAG---GAC ACAAAAGCTC AGAGCTCCAC AGTCTAGAGT CTAGACCAAC AGGCCTCCAC ACTCACGTCC

**orangutan**  ATGGAGAAGA TTCTTCCCCA GCTTCCTTCC AGAGAAGGAC ACAAAAGCTC AGAGCTCCAC AGTCTAGAGT CTAGACTCAC AGGCCTCCAC ACTCACGTCC

**human**  CAGAGATTTC CCTGGTCCCA CCTACTCCCA GTGGCAACCA GACTTCTGCA CATAGGAGAG ATGTCATACT CAGAGTCCAG CCTCCCACAT CCACAGGACC

**chimpanzee** CAGAGATTTC CCTGGTCCCA CCTACTCCCA GTGGCAACCA GACTTCTGCA CATAGGAGAG ATGTCATACT CAGAGTCCAG CCTCCCACAT CCACAGGACC

**orangutan**  CAGAGGTTTC CCTGGTCCCA CCTACTCCCA GTGGCAACCA GACTTCTGCA GATAGGAGAG ATGTCAAACT CAGAGTCCAG CCTCCCACAT CCACAGGACC

**human**  ACCTCTTCCT CCTCTGAGTC TTGTTAATAG GGCCATCCCC TGCCTTAGAC CTGGCCCAGT GGACTCTGAT CTTAC**----**A GCCAATATGG GGCAGCAAAG

**chimpanzee** ACCTCTTCCT CCTCTGAGTC TTGTTAATAG GGCCATCCCT TGCCTTAGAC CTGGCCCAGT GGACTCTGAT CTTAC**AAAC**A GCCAATATGG GGCAGCAAAG

**orangutan**  ACCTCTTCCT CCTCTGAGTC TTGTTAATAG GGCCATCCCC TGCCTTAGAC CTGGCCCAGT GGACTCTGAT CTTAC**AAAC**A GCCAATATGG GGCAGCAAAG

**human**  TGGGACATCT GTCTACAGGG CCAGTAGCCC CAGGTCATCT GCTTGCCAAA AGGAGGGGGA CCAGCCCCCG GGGGGAGCCC AGAGCTCGGC AGGGCTGGGG

**chimpanzee** TGGGACATCT GTCTACAGGG CCAGTAGCCC CAAGTCATCT GCTTGCCAAA AGGAGGGGGA CCAGCCCCCG GGGGGAGCCC AGAGCTCGGC AGGGCTGGGG

**orangutan**  TGGGACATCT GTCTACAGGG CCAGTAGCCC CAAGTCATCT GCTTGCCAAA AGGAGGGAGA CCAGCCCCCG GGGGGAGCCC AGAGCTCGGC AGGGCTGGGG

**human**  TTAGTAAGAA GAGAAAACAG GGTTAGTAGG GGCTGGGTTA GTAAGTCAGA GCACAGCACC AGCGGACAGG GCACCTCAGC AGACACACAC AGGAGTCGCT

**chimpanzee** TTAGTAAGAA GAGAAAACAG GGTTAGTAGG GGCTGGGTTA GTAAGTCAGA GCACAGCACC AGCGGACAGG GCACCTCAGC AGACACACAC AGGAGTCGCT

**orangutan**  TTAGTAAGAA GAGAAAACAG GGTTAGTAGG GGCTGGGTTA GTAAGTCAGA GCACAGCACC AGCGGACAGG GCACCTCAGC AGACACACAC AGGAGTCGCT

**human**  AA

**chimpanzee** AA

**orangutan**  AA

ENSG00000204626

**human**  ATGCACAGCC TGCCACGGAG TGGCTCTATC AGGCGCACAC ACAGCGACAC ACAGGCCACT GGCTGGCCTC CTCCCCAGCG CATTGGGGAC AGCCCAGGCC

**chimpanzee** ATGCACAGCC TGCCACGGAG TGGCTCTATC AGGCGCACAC ACAGCGACAC ACAGGCCACT GGCTGGCCTC CTCCCCAGCG CATTGGGGAC AGCCCAGGCC

**orangutan**  ATGCACAGCC TGCCATGGAG TGGCTCTATC AGGCGCGCAC ACAGCGACAC ACAGGCCACT GGCTGGCCTC CTCCCCAGCG TATTGGGGAC AGCCCAGGCC

**human**  CTTCTCCAGC ATTTCTGTCC TGCCCACCTT CCCTCTGTGG AGGAGCAGCC CAGACAGGAG ACCCTGTGGC CCTGCCCCAT GGCCCAGAGA AATGGGTGTG

**chimpanzee** CTTCTCCAGC ATTTCTGTCC TGCCCACCTT CCCTCTGTGG AGGAGCAGCC CAGACAGGA- -CCCTGTGGC CCT-CGCCAT GGCCCAGAGA AATGGGTGTG

**orangutan**  CTTCTCCAGC ACTTCTGTCC CGCCCACCTT CCCTCTGTGG AGGAGCAGCC CCGACAGGAG ACCCCGTGGC CCTCCTCCAT GACCCAGAGA AGTGGGTGCA

**human**  GGGCGGTGGC CTCTCCCCCA GGAAT**CCTCA TTCCT**GGGGC ATCAAGGCCC ACGGACTTAG ACCACCCTGG GCCCCCAGGC TAGAAAGATG CATGGTCCCA

**chimpanzee** GGGCGGTGGC CTCTCCCCCA GGAAT**----- -----**GAGGC ATCAAGGCCC ACGGACTTAG ACCACCCTGG GCCCCCAGGC TAGAAAGATG CATGGTCCCA

**orangutan**  GGGTGCTGGC CTCTCCCCCA GGAAT**----- -----**GAGGC AGCAAGGCCC ACGGACTTAG ACCACCCTGG GCCCCCAGGC TAGAAAGATG CATGGCCCCA

**human**  GAGTCAGAAT GGGCACCATG GCAACCCCAG CTACCCTGTG AGCCGAAGTG GCTGGGGAGC AGGAAGTCGA AGCCTCACAG AGAAAGTGGT CTCCGGGGAG

**chimpanzee** GAGTCAGAAT GGGCACCATG GCAACCCCAG CTACCCTGTG AGCCGAAGTG GCTGGGGAGC AGGAAGTCGA AGCCTCACAG AGAAAGTGGT CTCCGGGGAG

**orangutan**  GAGTCAGAAT GGGCACCGTG GCAACCCCAG CTACCCTGTG AGCCAAAGTG GCTGGGGAGC AGGAAGTCGA AGCCTCACAG AGAAAGTGGT CTCCGGGGAG

**human**  GAGGACCCAG CAGATGTGCA AAGAGA---- -GGAACACAC TCCTGTGGCC CCAGAGAGAG TGGTGGCCCG GACACCTGCC ACCTCCCCTG CCACTGA

**chimpanzee** GAGGACCCAG CAGACGTGCA AAGAGA---- -GGAACACAC TCCTGCGGCC CCAGAGAGAG TGGTGGCCCG GACACCTGCC ACCTCCCCTG CCACTGA

**orangutan**  GAGGACCCAG CAGACGTGCA AAGAGAAGAG AGGAGCACAC TCCTGTGGCC CCAGAGAGAG TGGTGGCCTG GACACCTGCC ACCTCCCCTG CCACTGA

ENSG00000198411

**human**  ATGAGAAGTT T----TTCTG TGCCTGGATT ATTTCACATA ATATCCTTCA GTTCCATCCA TGTTGTTGCA AATGATAGGA TCTCATTCTT TTTTATGGCA

**chimpanzee** ATGAGAAGTT T----TTCTG TGCCTGGATT ATTTCACATA ATATCCTTCA GTTCCATCCA TGTTGATGCA AATGATAGGA TCTCATTCTT TTTTATGGCA

**orangutan**  ATGAGAAGTT TGTTTTTCTG TGCCTGGATT ATTTCACATA ATGTCCTTCA GTTCCATCCA TGTTGTTGCA AATGATAGGA TCTCATTCTT TTTTATGGCA

**human**  GAATGGTTTT TCATTGTGTA TATGTACCAC ATTTCCTTTA TCCATTCATT TGTTGATGGA CACTTAGGTT GCCTCCAAAT CTTGGCTGTT GTGAACAGTG

**chimpanzee** GAATAGTTTT TCATTGTGTA TATGTACCAC ATTTCCTTTA TCCATTCATT TGTTGATGGA CACTTAGGTT GCCTCCAAAT CTTGGCTGTT GTGAACAGTG

**orangutan**  GAATAGTTTT TCATTGTGTG TATGTACCAC ATTTCCTTTA TCCATTCATC TGTTGATGGA CACTTAGGTT GCCTCCAAAT CTTGGCTGTT GTGAACAGTG

**human**  CTGTAATAAA CATGGGAGTG CAGAGATGTC TTCAATATAC TGATTTCCAT TCTTTTAGGT ATATACTTGG TAGTAGGATT GCTGGATCAT ATGGTAGCTC

**chimpanzee** CTGTAATAAA CATGAGAGTG CAGAGATGTC TTCAATATAC TGATTTCCAT TCTTTTAGGT ATATACTTGG TAGTAGGATT GCTGGATCAT ATGGTAGCTC

**orangutan**  CTGTAATAAA CATGGGAGTG CAGAGATGTC TTCAATATAC TGATTTCCAT TCTTTTAGGT ATATACTTGG TAGTAGGATT GCTGGCTCAT ATGGTAGCTC

**human**  TATTGTTAGG TTTATTTTTT ATTTAAAAAA ACATTTTTTT GAGACAG--T CTCACTCTGT TGCCTAGGCT GGAGTGCAAT GGCGCAATCT TGGCTCACTG

**chimpanzee** TATTGTTAGG TTTATTTTTT ATTTAAAAAA AT**-**TTTTTTT GAGACAG--T CTCACTCTGT TGCCTAGGCT GGAGTGCAAT GGCGCAATCT TGGCTCACTG

**orangutan**  TATTGTTAGG TTTATTTTTT ATTTAAAAAA AT**-**TTTTTTT GAGACAGAGT CTCACTCTGT TGCCTAGGCT GGAGTGCAAT GGCGCAATCT TGGCTCACTG

**human**  TAACCTCTGC CTCCCAGGTT CAAGTGATTC TCCTACCTCA GCCTCCTGAG TGCCTGGGAT TACAGGTGTG TGCCACCACA CTTGGCTAA

**chimpanzee** TAACCTCCAC CTCCCAGGTT CAAGTGATTC TCCTACCTCA GCCTCCTGAG TGCCTGGGAT TACAGGTGTG TGCTGCCACA CTCGGCTAA

**orangutan**  TAACCTCTAC CTCCCAGGTT CAAGTGATTC TCCTACCTCA GCCTCCCGAG TGGCTGGGAT TACAGGTGTG TGCCACCACA CTCAGCTAA

ENSG00000218478

**human**  ATGTACTTTT GTGGCCTTGG TGTCCGATGG GGCTGGGGGA GAGTGCTCTC CACTGACCCA GCAGCACACC CATGTGCAGT GCGCCTGCAT CTGTG-----

**chimpanzee** ATGTACTTTT GCGGCCTTGG TGTCCGATGG GGCTGGGGGA GAGTGCTCTC CACTGACCCA GCAGCACACC CATGTGCAGT GCGCCTGCAT CTGTG-----

**orangutan**  ATGTACTTTT GTGGCCTTGG TGTCCGATGG GGCTGGGGGA GAGTGCTCTC CACTGACCCA GCAGCACACC GATGTGCAGC GCGCATGCAT CTGTGCATCT

**human**  ---TGGGGGC AGCCACACCC CTTGGCTGCT GCTTCCTTGG GCTGCCTTTC TGGGGGCATG TGACTGGACC TACGAGGTCT GCACTGAGCT CCATTTGAAT

**chimpanzee** ---TGGGGGC AGCCACACCC CTTGGCTGCT GCTTCCTTGG GCTGCCTTTC TGGGGTCATG TGACTGGACC TACGAGGTCT GCACTGAGCT CCATTTGAAT

**orangutan**  GTGTAGGGAC AGCCACACCC CTTGGCTGCT GCTTCCTTGG GCTGCCTTTC TGGGGGCGTG TGCCTGGACC TACGAGGTCT GCACTGAGCT CCATTTGAAT

**human**  GATACCTTTC CTATCCCATT TCCCC**C**ACGG AAGCACCGCT TCAGGGTTAT TCAGTCCTCT GCCTCATGGC TGAAATTGCT CATCTCGTCT GCAGATGTCT

**chimpanzee** GATACCTTTC CTATCCTGTT TCCCC**-**ATGG AAGCACCGCT TCAGGGTTAT TCAGTCCTCT GCCTCATGGC TGAAATTGCT CATCTCGTCT GCAGATGTCT

**orangutan**  GATACCTTTC CTATCCCATT TCCCC**-**ACGG AAGCACCGCT TCAGGGTTAT TCAGTCCTCT GCCTCATGGC TGAAATTGCT CATCTCGTCT GCAGATGTCT

**human**  ACTATCCTGT CTACCTAATG CACTATTATG TATTGATTCT CCATGAGACA GAGAGAGAGA GAGACTATCA GATAGTTTAC ACCCAAAGGG TAGGTTTTTG

**chimpanzee** ACTATCCTGT CTACCTAATG CACTATTATG TATTGATTCT CCATGAGACA GAGAGAGAGA GAGACTATCA GATAGTTTAC ACCCAAAGGG TAGGTTTTTG

**orangutan**  ACTATCCTGT CTACCTAATG CACTATTATG TATTGATTCT CCATGAGACA GAGAGAGAGA GAGACTATCA GATAGTTTAC ACCCAAAGGG TAGGTTTTTG

**human**  TATATTTTTC CAGCCTTTTT TATTAAGGGG AAGGGGAGAG TTTAAAAACC CAAACCGTTG TGGTTTTAAG GTGTTTCATT TTTAA

**chimpanzee** TATATTTTTC CAGCCTTTTT TATTAAGGGG AAGGGGAGAG TTTAAAAACC CAAACCGTTG TGGTTTTAAG GTGTTTCATT TTTAA

**orangutan**  TATATTTTTC CAGCCTTTTT TATTAAGGGG AAGGGGAGAG TTTAAAAACC CAAACCGTTG TGGTTTTAAG GTGTTTCATT TTTAA

ENSG00000204380

**human**  ATGGCGGCTC TCATCTGCCC TGCCTCTCCG CAGCACTTTC CTTTTCTCCA C**-**AGCTTCTG GGACCCCACC TGGCTTCTCT CTCACCTTGC TACTTCTCAG

**chimpanzee** ATGGCGGCTC TCATCTGCCC TGCCTCTCCG CAGCACTTTC CTTTTCTCCA C**C**AGCTTCTG GGACCCCACC TGGCTTCTCT CTCACCTTGC TACTTCTCAG

**orangutan**  ATGGCGGCTC TCATCTGCCC TGCCTCTCCG CAGCACTTTC CTTTTCTCCA C**C**AGCTTCTG GGACCCCACC TGGCTTCTCT CTCACCTCGC TGCTTCTCAG

**human**  ACTCATCTGC CCATGGGCAC CTCCAGGAGT GCCCCAGGTC CTGTCTTGTC TTCATCTTTG CACTCTCCAA GGTGCCTTCT GCTCCTTGTC TTTAATACAA

**chimpanzee** ACTCATCTGC CCATGGGCAC CTCCAGGAGT GCCCCAGGTC CTGTCTTGTC TTCATCTTTG CACTCTCCAA GGTGCCTTCT GCTCCTTGTC TTTAATGCAA

**orangutan**  ACTCATCTGC CCACGGGCAC CTCCAGGAGT GCCCCAGGTC CTGTCTTGTC TTCATCTTTG CACTCTCCGA GGTGCCTTCT GCTCCTTGTC TTTAATACAA

**human**  CCTATGGACA CAGGGCCATA GGTTGGCACA CATCTGCCTT TAGCCCTGAC TGCTCTCTAG AATTGCGGAT TCTTTTCTCC AATGCTTTCT TGACACTGGC

**chimpanzee** CCTATGGACA CATGGCCATA GGTTGGCACA CATCTGCCTT TAGCCCTGAC TGCTCTCTAG AATTGCGGAT TCCTTTCTCC AATGCTTTCT TGACACTGGC

**orangutan**  CCTATGGACC CATGGCCATA GGTTGGCACA CATCTGCCTT TAGCCCTGAC TGCTCTCTAG AATTGCGGAT TCTTTTCTCC ATTGCTTTCT TGACACTGGC

**human**  ACATAGACAG CTAATTAGAC TTCTCAAACT GGACATTGTC AAAACTCTGA GCTGCTCACC CTTCCAAGCA TTCCTGTCCC TTCCCCCCAT CAACAGCACT

**chimpanzee** ACATAGACTG CTAATTAGAC TTCTCAAACT GGACATTGTC AAAACTCTGA GCTGCTCACC CTTCCAAGCA TTCTTGTCCC TTCACCCCAT CAACAGCACT

**orangutan**  ACGTAGACAG CTAATTAGAC TTCTCAAACT GGACATTGTC AAAACTCTGA GCTGCTCACC CTTCCAAGCA TTCTTCTCCC TTTCCCCCAT CAACAGCACT

**human**  TCTGTGCTTG CAGCTGATCC AGCCAAAGAT CTAGGTGTAT CCTTATTTCC CCCCTTTCCT CGCTCTTAA

**chimpanzee** TCTGTGCTTG CAGCTGATCC AGCCAAAGAT CTAGGTGTAT CCTTATTTCC CCCCTTTCCT CGCTCTTAA

**orangutan**  TCTGTGCTTG CAGCTGATCC AGCCAAAGGT CTAGGTGTAT CCTTATTTCC CCCGTTTCCT CGCTCTTAA

ENSG00000167117

**human**  ATGGGGAGGA GGAGTCCATT CAAACCGAGA AACAAAGTGT TTGGTTTTTC TTACCCC**TGG** TGTAGAAGCT ACCAACCTTT TCCAAGAAAG AGGGCCTGGC

**chimpanzee** ATGGGGAGGA GGAGTCCATT CAAACCGAGA AACTAAGTGT TTGGTTTTTC TTACCCC**TGA** TGTAGAAGCT ACCAACCTTT TCCAAGAAAG AGGGCCTGGC

**orangutan**  ATGGGGAGGA GGAGTCCGTT CAAACTGAGA AACAAAGTGT TTGGTTTTTC TTACGCC**TGA** TATAGAAGCT ACCAACTTTT TCCAAGAAAG AGGGCCTGGC

**human**  CCCCTTCTCG GGTCTGGCTG GGTGCCTGCT GTGCCTCTCT GGCCTCCCCT CCGAAGGGCA CCATTCCCTC GGGTGAGTAC TACCGGCCTG CACCGTCTTC

**chimpanzee** CCCCTTCTCG GGTCTGGCTG GGTGCCTGCT GTGCCTCTCT GGCCTCCCCT CCGAAGGGCA CCATTCCCTC AGGTGAGTAC TACTGGCCTG CACCGTCTTC

**orangutan**  CCACTTCTCG GGTCTGGCTG GGTGCCTGCT GTGCCTCTCA GGCCTCCCCT CCGAAGGGCA CCATTCTCTT GGGTGAGTAC TAGTGGCCTG CACTGTCTTC

**human**  CAGTGGGGAC AGCCTGAGAA GAGAGTCTGG AGCCTT--AC TTCAGTACCT TCCTTCACTG GCCTCACCCT GTGCAAATCA TGCCACACGC TGCAGCCTCC

**chimpanzee** CAGTGGAGAC AGCCTGAGAA GAGAGTCTGG AGCCTT--AC TTCAGCACCT TCCTTCACTG GCCTCACCCT GTGCAAATCA TGCCACACAC TGGAGCCTCC

**orangutan**  CAGTGGAGAC AGCCTGAGAA GGGAGTCTGG AGCCTTGAAC TTCAGTACCT TCCTTCACTG GCCTCACCCT GTGCAAATCA TGCCACACAC TGGAGCCTCC

**human**  TTTTCCCTAT CTATAAAATA AAAATGACCC TGCTCTATCT CACTGGGCTG GCAAGAACAC ACTGTTGTTA CCTTGCAGAC AGATGTGCTG AGGCTGTAGA

**chimpanzee** TTTTCCCTAT CTATAAAATA AAAATGACCC TGCTCTATCT CACTGGGCTG GCAAGAACAC ACTGTTGTTG CCTTGCAGAC AGACGTGCTG AGGCTGTAGA

**orangutan**  TTTTCCCTAT CTATAAAATA AAAATGACCC TGCTCTATCT CACTGGGCTG GCAAGAACAC ACTGTTGTTG CCTTGCAGAC AGATGTGCTG AGGCTGTAGA

**human**  AAGTGCTTTT TATTTGGTTG GGAGCTTGTG CATAAATGCG AGAGGGGCTG CACATCTGAC GGACTAG

**chimpanzee** AAGCGCTTTT TATTTGGTTG GGAGCTTGCG CATAAATGCG AGAGGGGCTG CACATCTGAC GGACTAG

**orangutan**  AAGTGCTTTT TATTTGGTTG GGAGCTTGTG CATAAATGTG AGAGGGGCTG CACATCTGAC GGACTAG

ENSG00000162968

**human**  **ATG**TATGGCT GCTATACACC AACAGCTTAT TCTACAAGAA GTGCCCCCGA GGAGGATTGG GTTAAGCTTT GCAAATTTGG CTTCCCAGGT AATGCGCTTC

**chimpanzee** **GTG**TATGGCT GCTATACACC AACAGCTTAT TCTACAAGAA GTGCCCCCGA GGAGGATTGG GTTAAGCTTT GCAAATTTGG CTTCCCAGGT AATGCGCTTC

**orangutan**  **GTG**TATGGCT GCTGTACACC AACAGCTTAT TCTACG-GAA GTGCCCCCGA GGAGGATTGG GTTAAGCTTT GCAAATTTGG CTTCCCAAGT AATGCGCTTC

**human**  ATTATTCTGC TCCCGACTTA CCCACCACAC CAGTGGGTAC CCGGAGCAGC ACCCACTTGG CAGAACTGAT GACTGCTTGG GCCCAGCGGA GTGCGCATTG

**chimpanzee** AGTATTCTGC TCCCGACTTA CCCACCACAC CAGTGGGTAC CCGGAGCAGC ACCCACTTGG CAGAGCTGAT GACTGCTTGG GCCCAGCGGA GTGCGCATTG

**orangutan**  ATTATTCTGC TCCCGACTTA CCCACCACAC CAGTGGGTAC CCGGAGCAGC ACCCACTTGG CAGAATTGAT GACTGCTTGG GCCCAGCGGA GTGCGCATTG

**human**  CGCTAACACG CGCACGGGAA TTGCACCCTT GCCGGAGCCT CCGCACCGTG CGCCCTTCAA AGAGCTGGCG ACCCCGCTCA CGTGTAAGCA ACCTCCCACT

**chimpanzee** CGCTAACACG CGCACGGGAA TTGCACCCTT GCCGGAGCCT CCGCACCGTG CGCCCTTCAA AGAGCTGGCG ACCCCGCTCA CGTGTAAGCA ACCTCCCACT

**orangutan**  CGCTAACACG CGCACGG-AA TTGCACCCTT GCCGGAGTCT CCGCACCGTG CGCCCTTCGA AGAGCTGGCG ACCCCGCTCA CGTGTAAGCA ACCTCCCACT

**human**  TTGAAACTAA TTCGCACCCG GGTCTTTCAC CCCAAAGGAC TTTGCTGCGG ACGCTGCTCT GACCCAAGAC GCGGGAGAGA AGTCCCAAAG GCTACAGCCA

**chimpanzee** TTGAAACTAA TTCGCACCCG GGTCTTTCAC CCCAAAGGAC TTTGCTGCGG ACGCTGCTCT GACCCAAGAC GCGGGAGAGA AGTCCCAAAG GCTACAGCCA

**orangutan**  TTGAAACTAA TTCGCACCCG CGTCTTTCAC CCCAAAGGAC TTTGCTGCGG ACGCTGCTCT GACCCAAGAC GCGGGAGAGA AGTCCCAAAG GCTACAGCCA

**human**  GGGGCTGGGG GACTCCTCTG CTCACCTTAG TCCTTGATTT CGAAGGCCCC AATTAA

**chimpanzee** GGGGCTGGGG GACTCCTCTG CTCACCTTAG TCCTTGATTT CGAAGGCCCC AATTAA

**orangutan**  GGGGCTGGGG GACTCCTCTG CTCACCTTAG TCCTTGATTT CGAAGGCCCC AATTAA

ENSG00000204292

**human**  ATGACCTTTT TCTCAACCAT GCGCAAGTGG TATTTCTGCG TGCTTCAAAT ATCACAGGCC GGATTGTCAG GAAACCAAGG AGGGGCCTTT TGGAAGAGAA

**chimpanzee** ATGACCTTTT TCTCAACCAT GCGCAAGTGG TATTTCTGCG TGCTTCAAAT ATCACAGGCC GGATTGTCAG GAAACCAAGG AGGGGCCTTT TGGAAGAGAA

**orangutan**  ATGACCTTTT TCTCAACCAT GTGCAGGTGG TATTTCTGCG TGCTTCAAAT ATCACAGGTC GGATTGTCAG GAAGCCAAGG AGGGGACTTT TGGAAGAGAA

**human**  TGATGCTGAG CATCCGGGTC CCTGGACAGC CAGCAGCCCT GAGGCAGACA GAAGTTTGGA CAACTCAGGA CAGGGAGCTT GGAGGGCAGC CCCTACATGT

**chimpanzee** TGATGCTGAG CATCCGGGTC CCTGGACAGC CAGCAGCCCT GAGGCAGACA GAAGTTTGGA CAACTCAGGA CAGGGAGCTT GGAGGGCAGC CCCTACATGT

**orangutan**  TGCTGCTGAG CATCCGGGTC CCTGGACAGC CAGCGGCCCT GAGACAGACA GAAGTTTGGA CAACTCAGGA CAGGGAGCTT GGAGGGCAGC CCCTACATGT

**human**  C**GGA**GTGGTC CCTACCTGTG TTGCCTCAAC TCTTCCTTCA AGGCAAGAGC CGCTAAGGAC AGTCAGGACA TCCTACCGCC CAAGGCTGTT GAGCTCGGCC

**chimpanzee** C**TGA**GTGGTC CCTACCTGTG TTGACTCAAC TCTTCCTTCA AGGCAAGAGC CGCTAAGGAC AGTCAGGACA TCCTACCGCC CAAGGCTGTT GAGCTCGGCC

**orangutan**  C**TGA**GTTGTC CCTACCTGTG TTGACTCAAC TCTTCCTTCA AGGCAAGAGC CGCTAAGGAT AGTCAGGACA TCCTACCGCC CAAGGC---- ----------

**human**  GGCCAAGGAC TACAGGAAGG ATGGCTGGGG AGGCCTCAGG AAACTTACAG TCACAGCGGA AGGCAAAGAG GAAGCAGGCA GCGGCTTCGT GGCAGGAGCA

**chimpanzee** GGCCAAGG-- TACAGGAAGG ATGGCTGGGG AGGCCTCAGG AAACTTACAA TCACAGCGGA AGGCAAAGAG GAAGCAGGCA GCGGCTTCGT GGCAGGAGCA

**orangutan**  ---------- ---------- ---------- ---------- ---------- ---------- ---------- ---------- ---------- ----------

**human**  GGAGGAAGGC GGTGGGGAGG TGCTACATAC TTTTAAACAA CTGGATCTTG TGA

**chimpanzee** GGAGGAAGGC GGTGGGGAGG TGCTACATAC TTTTAAACAA CTGGATCTTG TGA

**orangutan**  ---------- ---------- ---------- ---------- ---------- ---

ENSG00000205557

**human**  ATGTTTCGAT TTCACACAAA GAAAGAGCAC ACGTCCACCA TCTTCAGTGG GGGCTGTCTT TTGCTTCACT GGCAAGCAGG CACTGAATTT TTCTTGCATG

**chimpanzee** ATGTTTCGAT TTCGCACAAA GAAAGAGCAC ACGTCCACCA TCTTCAGTGG GGGCTGTCTT TTGCTTCACT GGCAAGCGGG CACTGAATTT TTCTTGCATG

**orangutan**  ATGTTTCGAG TTCGCACACA GAAAGAGCAC ACGTCCACCA TCTTCAGTGG GGGCTGTCTT TTGCTTCACT GGCAGTCGGG CACTGAATTT TTCTTGCATG

**human**  ACAAATCTGG AGGTTTACTG GTGAGAGAGC CAATGGGCAT TTTTTCCTGG AAAGAGTACA GCTCCATACC CAGTCCTAAC CCAACAGTGA T**-------**AT

**chimpanzee** ACAAATCTGG AGGTTTACTG GTGAGAGAGC CAATGGGCAT TTTTTCCTGG AAAGAGTACA GCTCCATACC CAGTCCTAAC CCAACAGTGA T**TCCAGGA**AT

**orangutan**  ACAAATCTGG AGGTTTACTG GTGAGAGAGC CAATGGGCTT TTTTTCCTGG AAAGAGTACA GCTCCATACC CAGTCCTAAC CCAACAGTGA T**TCCAGGA**AT

**human**  TTATCACTTT GGGGCAGGGC TGTATAGAGT GT**GTGTGTGT GTGTGTGTGT** GTGTGTGTGT GTGTGTGTGT GTGTGTGCGG GTTGGGGTGG TGTTGGGCCA

**chimpanzee** TTATCACTTT GGGGCAGGGC TGTATA---- --**-------- ----------** GTGTGTGTGT GTGTGTGTGT GTGTGTGCGT GTTGGGGTGG TGTTGGGCCA

**orangutan**  TAATCACTTT GGGGCAGGGC TGTACAGAGT GT**-------- ----------** GTATGTGTGT GTGTGTGTGT GTATGTGCGG GTTGGGGTGG TGTTGGGCCA

**human**  TCTCTGGCCT GTTACTAAGG TAACTAGGAC TATTTGTGTT CCAGCAGTCA TAGCCTGTGA TTGTGGGTGC ATCAGTTCTC TGCCTAGATC TCTTGTTACC

**chimpanzee** TCTCTGGCCT GTTACTAAGG TAACTGGGAC TATTTGTGTT CCAGCAGTCA TAGCCTGTGA TTGTGGGTGC ATCAGTTCTC TGCCTAGATC TCT-GTTACC

**orangutan**  TCTCTGGCCT GTTACTAAGG TAACTGGGAC TATTTGTGTT CCACCAGTCA TAGCCTGTGA TTGTAGGCAC ATCAGTTCTC TGCCTAGATC TCT-GTTACC

**human**  TTGTCTGCAC ATCAAGGGAG GGAGTTGAGC ACAGATACTT GTCAAGGGCC ATTGTAG

**chimpanzee** TTGTCTGCAC ATCAAGGGAG GGAGTTGAGC ACAGATACTT GTCAAGGTCC ATTGTAG

ENSG00000204506

**human**  ATGGGCCAGG TGTGGAATCT TGGAGCATGT CAGAAAGGAG AGAGGCTTTG TGGCCAGACA CACCTGGCTT GCAACCCCCC TCTCAATGCC ATGCATGTGA

**chimpanzee** ATGGGCCAGG TGTGGAATCT TGGAGCATGT CAGAAAGGAG AGAGGCTTTG TGGCCAGACA CACCTGGCTT GCAACCCCCC TCTCAATGCC ACGTATGTGA

**orangutan**  ATGGGCCAGG TGTGGAATCT TGGAGCATGT CAGAAAGGAG AGAGGCTTTG TGGCCAGACA CACCTGGCTT GCAACCCCCC TCTCAATGCC ATGCTTGTGA

**human**  CCTTGGGGGC ATCACTTAAC TTCTGTGGGC ATCAGTGTCC TCATCTTCAA AAATGCAAAT GGCAACATCT ATTTAGGATA TTTCTTATTA GGATTGATGA

**chimpanzee** CCTTGGGGGC ATCACTTAAC TTCTGTGGGC ATCAGTGTCC TCATCTTCAA AAATGCAAAT GGCAACGTCT ATTTAGGATA TTTCTTATTA GGATTGATGA

**orangutan**  CCTTGGGGGC ATCACTTAAC TTCTATGGGC ATCAGTGTCC TCATCTTCAA AAATGCAAAT GGCAACATCT ATTTAGGATA TTTCTTATTA GGATTGATCA

**human**  GATTTTA**TTA** AAAATTTCTA GCACATGGGG GGCTCCCAGA AAAGCTAGCT CCTATCATTT TTT-CAATGC GAGTAGTTCC TGGGTGAGCA AGACTTCCAT

**chimpanzee** GATTTTA**TAA** AAAATTTCTA GCACATGGGG GGCTCCCAGA AAAGCTAGCT CCTATCATTT TTT-CAACGC GTGTAGTTCC TGGGTGAGCA AGACTTCCAT

**orangutan**  GATTTTA**TAA** AAAATTTCTA GCACATGGTG GGCTCCCAGA AAAGCTAGAT CCTATCATTT TTTTCAATGC AAGTAGTTCC TGGATGAGCA AGACTTCCAT

**human**  TTCCCAGCCT TTCCCATGTT **T**GCAGAATAC ACAGAACATG GCACTGTTTG TCTGGCACAG GGGTATGTGG AGGCAGGAGG TGTCAGGAGG CTATGTTGGC

**chimpanzee** TTCCCAGCCT TTCCCATGTT **-**GCAGAACAC ACAGAACATG GCACTGTTTG TCTGGCACAG GGGTATGTGG AGGCAGGAGG TGTCAGGAGG CTATGTTGGC

**orangutan**  TTCCCAGCCT TTCCCATGTT **-**GCAGAACAC ACAGAACGTG GCACTGTTTG TCTGGCACAG GGGTATGTGG AGGCAGGAGG TGTCAGGAGG CTAGGTTGGC

**human**  TGCAGATGCC CCTCCTCTTG CTCACTGGGA GCCATTCCTG GCTGA

**chimpanzee** TGCAGATGCC CCTCCTCTTG CCCACTGGGA GCCATTCCTG GCTGA

**orangutan**  TGCAGATGCC CCTCCTCTTG CCCACTGGGA GCCATTCCTG GCCAA

ENSG00000204079

**human**  ATGATCTCGG TTCACTGCAA CCTCTGCCTC CCGGGTTCAA GCGATCCTCC TGCCTCAGCC TCCCAAGTAG CTGGAATTAC AGGCGTGAGG CACTGCATGG

**chimpanzee** ATGATCTCGG CTCACTGCAA CCTCTGCCTC CCGGGTTCAA GCGATCCTCC TGCCTCAGCC TCCCAAGTAG CTGGAATTAC AGGCGTGAGG CACTGCATGG

**orangutan**  ATGATCTCGG CTCACTGCAA CCTCTGCCTC CCGGGTTCAA GCGATCCTCC TGCCTCAGCC TCTCAAGTAG CTGAGATTAC AGGCGTGAGG CACTGCATGG

**human**  CCTCTGGGGC AGTTTTAAAT AAAGTGAGAA GGCATCAGTG CAGTGGAGAC TTAGAGGTGA GAG**--**GGAGC CATGGCTCCC TGGGGGAAGC CCCCTGGGGG

**chimpanzee** CCTCTGGGGC AGTTTTAAAT AAAGTGAGAA GGCGTCAGTG CAGTGGAGAC TTAGAGGTGA GAG**AG**GGAGC CGTGGCTCCC TGGGGGAAGT CCCCTGGGGG

**orangutan**  CCTCTGGAGC AGTTTTAAAT AAAGTGAGAA GGCATCAGTG CAGTGGAGAC TTGGAGGTGA GAG**AG**GGAGC CGTGGCTCCC TGGGGGAAGC CCCCTGGGGG

**human**  AAGAGTGTTC CAGGCAGGGG AACAGCAAGC AGAAAAGGTC CTGGGGCAGG TGTGATCGGG AACAGCAAAG AAGCCAGCAC GGGCCGGGCA CAGTGGAGCG

**chimpanzee** AAGAGTGTTC CAGGCAGGGG AACAGCAAGC AGAAAAGGTC CTGGGGCAGG TGTGATCGGG AACAGCGAAG AAGCCAGCAC GGGCCGGGCA CAGTGGAGCG

**orangutan**  AAGAGTGTTC TAGGCAGGGG AACAGCAAGC AGAAAAGGTC CTGGGGCAGG TGTGATCGGG AACAGCAAAG AAGCCAGCAC GGGCCGGGCA CAGTGGAGCG

**human**  CACCTGTAAT CCCAGCTACT CAGGAGGCCA AGGCAGGAGG ATTGCTTGAG CCTAGGAGTT TGATATCAGC CTGGGCAACA TATCAAGACC TCATCTCTAT

**chimpanzee** CACCTGTAAT CCCAGCTACT CAGGAGGCCA AGGCGGGAGG ATTGCTTGAG CCTAGGAGTT TGATATCAGC CTGGGCAACA TATCAAAACC TCATCTCTAT

**orangutan**  CGCCTGTAAT CCCAGCTACT AAGGAGGCCG AGGCGGGAGG ATTGCTTGAG CCTAGGAGTT TGATATCAGC CTGGGCAACA TATCAAGACC TAATCTCTAT

**human**  TAACAAATTA AAAGAAAAAA GAGGCTAG

**chimpanzee** TAACAAATTA AAAGAAAAAA GAGGCTAG

**orangutan**  TAACAAA--- --AGAAAAAA GAGACTGG

ENSG00000205201

**human**  ATGACACCTG GCCCAAATCA TCACCCAGGG AGGAGGGCAC AGCTGAGCAG AACTTCTCCC TATATCTTTC TGCCCCATCA TGAGTCCATT TATCAGCAAG

**chimpanzee** ATGACACCTG GCCCAAATCA TCACCCAGAG AGGAGGGCAC AGCTGAGCAG AACTTCTCCC TATATCTTTC TGCCCCATCA TGAGTCCATT TATCAGCAAG

**orangutan**  ATGACACCTG GCCCAAATCA TCACCCAGGG AGGAGGGCAC AGCTGAGCAG AACTTGTCCC TGTATCTTTC TGCCCCATCA TGAGTCCATT TATCAGCAAG

**human**  CATACAGACA TCCCTTGAGG GCAGCTCCTG AGGAGGTTGC AGGATGCGGG ATCCTGAGAT CTTTGCATTC AAGCAAGTCA GGCCT**----**A GCATGGGGCA

**chimpanzee** CATACAGACA TCCCTTGAGG GCAGCTCCTG AGGAGGTTGC AGGATGCGGG ATACTGAGAT CTTTGCATTC AAGCAAGTCA GGCCT**GCCT**A GCATGGGGCA

**orangutan**  CATACAGACA TCCCTTGAGG GCAGCTCCTG AGGAGGTTGC AGGATGCGGG ATCCTGAGAT CTTTGCATTC AAGCAAGTCA GGCCT**GCCT**A GCATGGGGCA

**human**  CCCTGCCTGA CCTGGAAGAG GACCCGGAAG CAGAGGGCAG TGAGCTGAGG GCCTTCCCAG CTCCTGCCCC AAGCTGGCAG CAGACCTGCC ACCAGGCTCT

**chimpanzee** CCCTGCCTGA CCTGGAAGAG GACCCGGAAG CAGAGGGCAG TGAGCTGAGG GCCTTCCCAG CTCCTGCCCC AAGCTGGCAG CAGACCTGCC ACCAGGCTCT

**orangutan**  CCCTGCCTGA CCTGGAAGAG GACCCAGAAG CAGAGGGCAG TGAGCTGAGG GCCTTCCCAG CTCCTGCCTC AAGCTGGCAG CAGACCTGCC GCCAGGCTCT

**human**  GGGGAAGAGC TGCTTCTGTG GGCTTTCGCC ATCCTCACGT CCCCTAGAGC TGCCCCCTCC TTCCTGTCCC TTCTTCTCAA AAGCACCATG GGTCAGGATT

**chimpanzee** GGGGAAGAGC TGCTTCTGTG GGCTTTCGCC ATCCTCACGT CCCCCAGAGC TGCCCC-TCC TTCCTGTCCC TTCTTCTCAA AAGCACCATG GGTCAGGATT

**orangutan**  GGGGAAGAGC TGCTTCTGTG GGCTTTCGCC GTCCTCACGT CCCCTAGAGC TGCCCC-TCC TTCCTGTCCT TTCTTCTCAA AAGCACCATG GGTCAGGATT

**human**  AGAGGGTCTG TTTGTTCTCT GATCTAA

**chimpanzee** AGAGGGTCTG TTTGTTCTCT GATCTAA

**orangutan**  AGAGGGTCTG TTTGTTCTCT GATCTAA

ENSG00000204412

**human**  ATGAGGGTGG GCTGGAGAGG AGCTGTTTGT CCCGCCTCC**C GA**CCCCGAGG AGGGCATAGT CCACAGGCTA TTTTAGGGAG CAAGAACTGG CCAGTCAGAA

**chimpanzee** ATGAGGGTGG GCTGGAGAGG AGCTGTTTGT CCCGCCTCC**T GA**CCCCGAGG AGGGCATAGT CCACAGGCTA TTTTAGGGAG CAAGAACTGG CCAGTCAGAA

**orangutan**  ATGAGGGTGG GCTGTCGAGG AGCTGTTTGT CCTGCCTCC**T GA**CTCCGAGG AGGGCATAGT CCACAGGCTA TTTTAGGGAG CAAGAACTGG CCAGTCAGAA

**human**  TGTGCCTGCG CCTCTCCCCA AGACAACAGC ACCATCAAAG GGGAACATCT TTGTCTTGGG GGAGCCATGT GGAATTGTAC CTAGAACAGA TTGTGAACAG

**chimpanzee** TGTGCCTGCG CCTCTCCCCA AGACAACAGC ACCATCAAAG GGGAACATCT TTGTCTTGGG GGAGCCATGT GGAATTATAC CTAGAACAGA TTGTGAACAG

**orangutan**  TGTGCCAGTG CCTCTCCCCA AGACAACAGC ACCATCAAAG GGGAACATCT TTGTCTTGGG GGAGCCACGT GGAATTGTAC CTAGAACAGA TTGTGAACAG

**human**  GGGTGCCTGT CAATTTACAT TTATCAGGAC TCGTTTCTTT TCCCTCCCAG ACTTGCCCTG CAAATCTCAT GGTGGGGTGG GGATCAAGGA GAAGAGGGCT

**chimpanzee** GGGTGCCTGT CAATTTACAT TTATCAGGAC TCGTTTCTTT TCCCTCCCAG ACTTGCCCTG CAAATCTCAT GGTGGGGTGG GGATCAAGGA GAAGAGGGCT

**orangutan**  GGGTGCCTGT CAATTTACAT TTATCAAGAC TTGTTTCTTT TCCCTCCCAG ACTTGCCCTG CAAATCTCCT GGTGGGGTGG GGATCAAAGA GAAGAGGGCT

**human**  TATCTTGACT TTCATGATCT TAGTGTTAAT GACAGTTACC CAGGATGGAG GTTTTTAGCC CCTTTCTTGG CCCTAGACCC AATGACCCCT TCCATGATAT

**chimpanzee** TATCTTGACT TTCATGATCT TAGTGTTAAT GACAGTTACC CAGGATGGAG GTTTTTAGCC CCTTTCTTGG CCCTAGACCC AATGGCCCCT TCCATGATAT

**orangutan**  TATCTTGACT TTCACGATCT TAGTGTTAAT GACAATTACC CAGGCTGGAG GTTTTTAGCC CCTTTCTTGG CCCTAGACCC AATGGCCCCT TCCATGATAT

**human**  TTTTCAAAGT CCAGTGA

**chimpanzee** TTTTCAAAGT CCAGTGA

**orangutan**  TTTTCAAAGT CCAGTGA

ENSG00000176911

**human**  ATGACGCCCC TTCCTTATCG TTCTAGAGTC AAGCCTAGAT CCCCTACAAA TACCCATGCA CACTTAGAGC TCACTCTGGT TAAATGTTCC AAGAAGGATG

**chimpanzee** ATGACGCTGC TTCCTTATCA TTCTAGAGTC AAGCCTAGAT CCCCTACAAA TACCCATGCA CACTTAGAGC TCACTCTGGT TAAATGTTCC AAGAAGGATG

**orangutan**  GTAATGCTGC C-CCTTATCA TTCTAGAGTC AAGCCTAGAT CCCCTACAAA TACCCATGCA CACTTAGAGC TCACTCTGGT TAAATGTTCC AAGAAGGATG

**human**  TTGGATGGTC ACTTGGTATC ATCCCATGGA GT**TCA**GGATA CCCCCGCCCT CTGAGCAGCC ATTCCTCTCC CGAGCACATT CCCCTAGAGA AGCTCTGCAC

**chimpanzee** TTGGATGGTC ACTTGGTATC ATCCCATGGA GT**TGA**GGATA TGCCCGCCCT CTGAGCAGCC ATTCCTCTCC CGAGCACATT CCCCTAGAGA AGCTCTGCAT

**orangutan**  TTGGATGGTC ACTTGGTATC ATCCCATGGA GT**TGA**GGATA CGCCCGCCCT CTGAGCAGCC ATTCCTCTCC CGAGCACGTT CCCCTAGAGA AGCTCTGCAC

**human**  GTGTGCACAG GCACCAGCTG CAGGAGTGCC ATCCACAGCA CAGCTGTTTA TGAAAAACCG TAATCAACCC AAACATCTGT ATGAAGAAAA ACGAAGAAAT

**chimpanzee** GTGTGCACAG GCACCAGCTG CAGGAGTGCC ATCCACAGCA CAGCTGATTA TGAAAAACTG TAATCAACCC AAACATCTGT ATGAAGAAAA ACGAAGAAAT

**orangutan**  GTGTACACAG GCACCAGCTG CAGGAGTACC ATCCACAGCA CAGCTGTTTA TGAAAAACCG TAATCAACCC AAACATCTGT ATGGAGAAAA ATGAAGAAAT

**human**  GCAGAGTGGC ATGTTCACAT GATGGAATAC TATGCAGCTG AGAACATGAA CAATTGGAGC CATGGCATGA ATGAAGCTGA GAGATTTGAA GCGTCAACCA

**chimpanzee** GCAGAGTGGC ATGTTCACAT GATGGAATAC TATGCAGCTG AGAACATGAA CAATTGGAGC CATGGCATGA ATGAAGCTGA GAGATTTGAA GCGTCAACCA

**orangutan**  GCAGAGTGGC ATGTTCACAT GATGAAATAC TATGCAGCTG AGAACATGAA CAATTGGAGC CATTGCATGA ATGAAGCTGA GAGATTTGAA GTGTCAACCA

**human**  AGTAA

**chimpanzee** AGTAA

**orangutan**  AGTAA

ENSG00000180838

**human**  ATGGGGAGGG ACAGATGGGT GGAGGAGTGG AAGATGGAGG ATGGGGAGGG ACAGATGAGT GGAGGAGTGG CAGATGGAGG AGGATGGCTG GAGGGACAGA

**chimpanzee** ATGGGGAGGG ACAGACGGGT GGAGGAGTGG AAGATGGAGG ATGGAGAGGG ACAGACGGGT GGAGGAGCGG CAGATGGAGG AGGATGGCTG GAAGGACAGA

**orangutan**  ATGGGGAGGG ACAGACGGGT GGAGGAGTGG AAGATGGAGG ATGGGGAGGG ACAGACGGGT GGAGGAGTGG CAGATGGAGG AGGATGGCTG GAGGGACAGA

**human**  TGCTGGGTGA TTGTTGGGGG TGCAGGTGCG GGACGGGTCG GGGACAGGTG GAAGGAAAAC AGCACACAGA TGCGGACAGA TGGCCAGAAA GA**TTA**ATAGT

**chimpanzee** TGCTGGGTGA TTGTTGGGGG TGCAGGTGCG GGACGGGTCG GGGACAGGTG GAAGGAAAAC AGCACATAGA TGCAGACAGA TGGCCAGAAA GA**TGA**ATAGT

**orangutan**  TGCTGGGTGA TTGTTGGGGG GGCAGGTGCG GGACAGGTGG GGGACAGGTG GAAGGAAAAC AGCACATAGG TGTGGGCAGA TGGCCAGAAA GA**TGA**ATAGT

**human**  TCAGTGGCAG AGACCTAGGC CTGGCGGCTG TCGTCCCCCC ACTCACGGAT GCGCGCGGTC CAGCACTGCT CCCCCAGCTG GGTGCAGTTC TCCACCTGCA

**chimpanzee** TCAGTGGCAG AGACCTAGGC CTGGCGGCTG TCGTCCCCCC ACTCACGGAT GCGCGCGGTC CAGCACTGCT CCCCCAGCTG GGTGCAGTTC TCCACCTGCA

**orangutan**  TCAGTGGCAG AGACCTAGGC CTGGCGGCTT TCGTCCCCCT ACTCACGGAT GCGCTCGGTC CAGCACTGCT CCTCCGGCTG GGTGCAGTTC TCTACCTGCA

**human**  GGCAGTCCTC GTTGCTCACC TGGGCTTTGC AGGAGTAGCA CAGCAGGGCA GTGCCTGTGG GGTGGAGTGG GAGGGGCCTC AGAGGCTGCC CCATAG

**chimpanzee** GGCAGTCCTC GTTGCTCACC TGGGCTTTGC AGGAGTAGCA CAGCAGGGCA GTGCCTGTGG GGTGGAGCGG GAGGGGCCTC AGAGGCTGCC TCATAG

**orangutan**  GGCAGTCCTC GTTGCTCACC TGGGCCTTGC AGGAGTAGCA CAGCAGGGCA GTGCCTGTGG GGTGGAGTGG GAGGGGCCTC AGAGGCTGCC CCATAG

ENSG00000206110

**human**  ATGTTCTTCT CCATTCAGCT TCATGGAGAG ACTTTCCCAG GTTTCAGCCC ACGC**TAC**GAA GCTAGACAGG AGAGTGGCCC CCACCCGCCC TGTTTGGAAC

**chimpanzee** ATGTTCTTCT CCATTCAGCT TCATGGAGAG ACTTTCCCAG GTTCCAGCCC ACGC**TAG**GAA GCTAGACAGG AGAGTGGCCC CCACCCGCCC TGTTTGGAAC

**orangutan**  ATGTTCTTCT CCACTCAGTT TCACGGAGAG ACTTTCCCAG GT-CCAGCCC ACGC**TAG**GAG GCTAGACAGG AGAGTGGCCC CCACCCGCCC TGTTTGGAAC

**human**  CCAGCTGGGG CATGCGGTGT TTGGAACACA TGGGCAGATT GGGGAGAGGC CAGAAGAGCC TCATCCAGAG CCAAGGACAA GCCTTGAGGC TGAAGAGCTC

**chimpanzee** CCAGCTGGGG CATGTGGTGT TTGGAACACA TGGGCAGACT GGGGAGAGGC CAGAAGAGCC TCATCCAGAG CCAAGGACAA GCCTTGAGGC TGAAGAGCTC

**orangutan**  CCAGCTGGGG CATGCGGTGT TTGGAACACA TGGGCAGATG GGGGTGAGGC CAGAAGAGCC TGATCCAGAG CCAAGGACAA GCCTTGAGGC TGAAGAGCTC

**human**  ACCAAGGCCA CTACAAGAGA GCAAAAAAAA GAGCCTCAGA GACTCCTCGA TAAATAACCA GAACAGAGAG CAAAGGTGGA GAGCAGGAGG GCAAGGCACT

**chimpanzee** ACCGAGGCCA CTACAAGAGA GCAAAAAAAA GAGCCTCAGA GACTCCTCGA TAAATAACCA GAACAGAGAG CAAAGGTGGA GAGCAGGAGG GCAAGGCACT

**orangutan**  ACCGAGGCCA CTACAAGAGA GAAAGAAAAA GAGCCTCAGA GATTCCTCAA TAAATAACCA GAACAGAGAG CAAAGGTGGA GTGCAGGAGG GCAAGGCACT

**human**  GAGACTTTAG AAGCTTCCAG GGAGAAGAAG A---GTCAGA TGCTTGAAGA TGCCTCCTCA ATGCTGCAAA GCTCTGAGGG AAAGTGGCTT TGA

**chimpanzee** GAGACTTTAG AAGCTTCCAG GGAGAAGAAG A---GTCAGA TGCTTGAAGA TGCCTCCTCA ATGCTGCAAA GCTCTGAGGG AAAGTGGCTT TGA

**orangutan**  GAGACTCTAG AAGCTTCCAG GGAGAAGAAG AAGAGTCAGA TGCTTGAAGA TGCCTCCTCA ATGCTGCAAA GCTCTGAGGG AAAGTGGCTT TCA

ENSG00000214467

**human**  ATGAAAAGAA AACAATTTAA AACTTTTGGG CTAAATAATG CTATTATTTT CCCAGAGATG CTGCACCTGA GATCTACTGT CTCTTTATCA ATAGGGGATA

**chimpanzee** ATGAAAAGAA AACAATTTAA AACTTTTGGG CTAAATAATG CTATTATCTT CCCAGAGATT CTGCACCTGA GATCTACTGT CTCTTTATCA ATAGGGGATA

**orangutan**  ATGAAAAGAA AACAATTTAA AACTTTTGGG CTAGATAATG CTATTATCTT CCCAGAGATT CTGCACCTGA GATCTACTGT CTCTTTATCA ATAGGGGATA

**human**  TTTGTAAGTG TCTGAGAGGT GTTTTTGTGT TTTGTCTGTT TGTTTGTTTG TTTGTTT**GTT TGTTT**TTGAG ATGGAGTCTC ATTCTGTCAC CCAAGCTGGA

**chimpanzee** TTTGTAAGTG TCTGAGAGGT GTTTTTGTGT TTTGTCTGTT TGTTTGTTTG TTTGTTT**--- -----**TTGAG ATGGAGTCTC ATTCTGTCAC CCAAGCTGGA

**orangutan**  TTTGCAGGTG TCTGAGAGGT GTTTTTGTGT TTTGTTTGTT TGTTTGTTTG TTTGTTT**--- -----**TTGAG ATGGAGTCTC ATTCTGTCAC CCAAGCTGAA

**human**  GGACAGTAGC AGGATCTCAG CTCACTGCAA CCTCAGCCTC CTGGGTTCAA GCGATTCTCC TGACTCAGCC TCCCAAGTAG CTAAGATTAC AGGCATGCAC

**chimpanzee** GGACAGTAGC AGGATCTCAG CTCACTGCAA CCTCAGCCTC CTGGGTTCAA GCGATTCTCC TGACTCAGCC TCCCGAGTAG CTAGGATTAC AGGCATGCAC

**orangutan**  GGACAGTAGT GAGATCTCAG CTCACTGCAA CCTCAGCCTC CTGGGTTCAA GCGATTCTCC TGCCTCAGCC TCCCGAGTAG CTAGGATTAC AGGCGTGCAC

**human**  CACCACATCC AGCTAACTTT TGTATTTTTA GTAGAGACGA GGTTTCTCCA ATGTTGGCCA GGCTGGTCTC AAACTCCTGA CCTCAGGTGA

**chimpanzee** CACCACATCC AGCTAATTTT TGTATTTTTA GTACAGACGA GGTTTCTCCA ATGTTGGCCA GGCTGGTCTC AAACTCCTGA CCTCAGGTGA

**orangutan**  CACCACATCC AGCTAATTTT TGTATTTTTA GTAGAGACGA GGTTTCTCCA GTGTTGGCCA GGCTGGTCTC AAACTCCTGA CCTCAGGTGA

ENSG00000136242

**human**  ATGACGATGG CCGCCAGGTG GGACAGGTCC CCAGTCAGCC GGAAAATGTT CATGGCGGCG GCGGCGGTGG CGGTCGGCGC AGCGCGGCGG CCCCGGGGCT

**chimpanzee** ATGACGATGG CCGCCAGGTG GGACAGGTCC CCAGTCAGCC GGAAAATGTT CATGGCGGCG GCGGCGGTGG CGGTCGGCGC AGCGCGGCGG CCCCGGGGCT

**orangutan**  ATGACGATGG CCGCCAAGTG GGACAGGTCC CCAGTCAGCC GGAAGATGTT CATGGCGGCG GCGGCGGT-- ----CGGCGC AGCGCGGCGG CCCCGGGGCT

**human**  GGGCGGCTCA GGAGGCGGCG GCCCCTGAGA GGAAGCGGCG AAGATGGCGA GATCGCCCGC GACGTGGCCA GGGACGTGGC CGAACTGCCG GCGCGCGCGC

**chimpanzee** GGGCGGCTCA GGAGGCGGCG GCCCCTGAGA GGAAGCGGCG AAGATGGCGA GATCGCCCGC GACGTGGCCA GGGACGTGGC CGAACTGCCG GCGCGCGCGC

**orangutan**  GGGCGGCTCA GGCAGCGGCA GCCCCTGAGA GGAAGCGGCG AAGATGGCGA GAGCGCCCGC GACGTGGCCA GGGACGTGGC CGAACTGCCG GCGCGCGCGC

**human**  GCGCGGGGCA CCCTGGGAGA GCGGGGCCAG GCCCCGCCCC TT**-------- ---**AAAGGGG GCGCGCCCCA GCGCAGGCTC AGCAGGTCAG CTCGCAGGGA

**chimpanzee** GCGCGGGGCA CCCTGGGAGA GCGGGGCCAG GCCCCGCCCC TT**CCCCGCCC CTT**AAAGGGG GCGCGCCCCA GCGCAGGCTC AGCAGGGCAG CTCGCAGGGA

**orangutan**  GCGCAGGGCA GCCTGGGAGA GCGGGGCCAG GCCCCGCCCC TT**CTCCGCCC CTT**AAAGGGA GCGCGCCCCA GCGCAGGCTC AGCAGTGCCC CTCGCAGGGA

**human**  CTCCCTTGCA AGAAGACCCC GGACTCTGCC AAATTTTCCC TGCCGGGTTT CCGGCTTCTC CTCGGGTGCC AGCAGTTTCC TTTACTTTTT TGAAATAA

**chimpanzee** CTCCCTTGCA AGAAGACCCC GGACTCTGCA AAATTTTCCC TGCCGGGTTT CCGGCTTCTC CTCGGGTGCC AGCAGTTTCC TTTACTTTTT TGAAATAA

**orangutan**  CTCCCTTGCA AGAAGACCCC GAACTCTGCC AAATTTTCCC TGCCGGGTTT CCGGCTTCTC CCCGGGTGCC AGCAGTTTCC TTTACTTTTT TGAAATAA

ENSG00000206096

**human**  ATGGAAGGTG ATTGTCTCAA GGTGGTCTGC TCTTCGCAAC TCTCCTTCTT TCTGGTAACC TCGCCCAGCA TCCCTCTAGG TCGAGGGAGG GGTGGTGTTG

**chimpanzee** ATGGAAGGTG ATTGTCTCAA GGTGGTCTGC TCTTCACAAC TCTCCTTCTT TCTGGTAACC TCGCCCAGCA TCCCTCTAGG TCGAGGGAGG GGTGGTGTTG

**orangutan**  ATGGAAGGTG ATTGTCTCAA GGTGGTCTGC TCTTCGCAAC TCTCCTTCCT TCTGGTAACC TCGCCCAGCG TCCCTCTAGG TCGAGGGAAG GGTGGTGTTG

**human**  GCCTCATTGC TTTTAACCTG GATTCCTGTA CCATCCCTCA TGGTTCTCTC CTACAAACCT TTGCAAAGAA TCCCTCCCCA CAC**GAG**CAGA ATTTTAGTGC

**chimpanzee** GCCTCATTGC TTTTAACCTG GATTCCTGTA CCATCCCTCA TGGTTCTCTC CTACAAACCT TTGCAAAGAA TCCCTCCCCA CAC**TAG**CAGA ATTTTAGTGC

**orangutan**  GCTTCAGTAC TGCTAACTTG GATTCCTGTA CCATCCCTCA TGGTTCTCTC CTACAAACCT TTGCAAAGAA ACCCTCCCCA TAC**TAG**CAGA ATTTTAGTGC

**human**  ATGCTCTCTG TGTTCTTCTA AGGTCCTCTT AGGGAATAGA CAATTCTGTA GGCACTTTCA TTGCAAACAG AATCCCAGTG AAGAAATGGT AGCGTCAGTC

**chimpanzee** ATGCTCTCTG TGTTCTTCTA AGGCCCTCTT AGGGAACAGA CAATTCTGTA GGCACTTTCA TTGCAAACAG AATCCCAGTG AAGAAATGGT AGCGTCAGTC

**orangutan**  ATGCTCTCTG TGTTCTTCTA AGGCCCTCTC AGGGAATAGA CAATTCTGTA GGCACTTTCG TTGCAAACAG AATCTCAGTG AAGAAATGGT AGCATCAGTC

**human**  CAAGATACTA ATCAACATGG CAATCTTCAC TACAGGATGA GAACTGTTTT CCCTTTGCCC CAGGTAGGAC ATAGGCCGCC TTAG

**chimpanzee** CAAGATACTA ATCAACATGG CAATCTTCAC TACAGGATGA GAACTGTTTT CCCTTTGCCC CAGGTAGGAC ATAGGCCACC TCAG

**orangutan**  CAAGATACTA ATCAACACAG CAATCTTCAC TACAGGATGA GAATTGTTTT TCCTTTGCCC CAGGTAGGAC ATAGGCTGCC TCAG

ENSG00000204581

**human**  ATG**-**GAGAAA GCCTCAGGTG CACTAAGGTG CCAACAGAAA CGATGCATCT CAGAGTTGAC AGTCATGACT CAAATAGGAC GTGAAGGCAA CATGTGGGTG

**chimpanzee** ATG**G**GAGAAA GCCTCAGGTG CACTAAGGTG CCAACAGAAA CAATGCATCT CAGAGTTGAC AGTCATGACT CAAATAGGAC GTTAAGGCAA CATTTGGGTG

**orangutan**  ATG**G**GAGAAA GCCTCAGGTG CACTAAGGTG CCAACAGAAA CAATGCATCT CGGAGTTGAC AGTCCTGACT CAAATAGGAC ATTAAGGCAA CATTTGGGTG

**human**  AGAGTAGCAG CTATGGGTGT AAATATGATC AAATATGGGG GAGGTGTCCA GATTTCTTGG ACATGGTTAC AATCAGTATT TATTTTCTCT CTTAGTGAAC

**chimpanzee** AGAGTAGCAG CTATGGGTGT AAATATGATC AAATATGGGG GAGGTGTCCA GATTTCTTGG ACATGGTTAC AATCAGTATT TATTTTCTCT CTTAGTGAAC

**orangutan**  AGAGTAGCAG CTATGGGTGT AAATATGATC AAATACGGGG GAGGTGTCCA GATTTCTCGG ACATGATTAC AATCAGTATT AATTTTCTCT CTTAGTGAAC

**human**  GAGTTTTTGG TTTTTCAATA CTGCTAATTT TACAGGCAAT TCACTACGTT CCCTGGGAGG TGGAGTGGCC TTCCTCCCTG CTGTGCGTGG GTTACACAGC

**chimpanzee** GAGTTTTTGG TTTTTCAATA CTGCTAATTT TACAGGCAAT TCACTACGTT CCCTGGGAGG TGGAGTGGCC TTCCTCCCTG CTGTGCGTGG GTTACACAGC

**orangutan**  GAGTTTTTGG TTTTTCAATA CTGCTAATTT TACAGGCAAT TCACTATGTT CCCTGGGAGG TGGAGTGGCC TTCCTCCCTG CTGTGCGTGG GTTACACAGC

**human**  CTGCCTCACT TCCCTGTGGG TCCTTCATCA CCTGCATGCT CACATGATTC CATTATTTGA GCTCATGGCA GGAAATAGAA CCTGA

**chimpanzee** CTGCCTCACT TCCCTGTAGG TCCTTCATCA CCTGCATGCT CACATGATTC CATTATTTGA GCTCATGGCA GGAAATAGAA CCTGA

**orangutan**  TCACCTCACG TCCCTGCAGG TCCTTCATCA CCTGCATACT CGCATGATTC CATTATTTGA GCTCATGGCA GGAAATAGAA CCTGA

ENSG00000205424

**human**  **ATG**TGTTCA**C GA**TCCTCCCA TGGAGGGGCT CAGAGGAGCG GCCTAAGAGG AGATGCCTGC ACTGTGCAGG AAAGAGGGGC TCCCTGCAGA GCCAGTGCCG

**chimpanzee** **GTG**TGTTCA**T GA**TCCTTCCA TGGAGGGGCT CAGAGGAGCG GCCTAAGAGG AGATACCTGC ACTGTGCAGG AAAGAGGGGC TCCCTGCAGA GCCAGTGCCG

**orangutan**   **GTG**TGTTCA**T GA**TCCTCCCA TGGAGGGGCT CAGAGAAGCA GCCTCAGAGG AGATGCCTGC ACTGTGCAGG AAAGGGGGGC TCCCTGCAGA GGCAGTGCCG

**human**  TTGGTGGGGC TCAGGCTCCC AGGGTAGGGG CAGGAGTGGT CTCCACAGTG CACATTTGCA CGTATGTTAG GACGAGGCTA TGGGGCACAG AGGGGCCATT

**chimpanzee** TTGGTGGGGC TCAGGCTCCC AGGGTAGGGG CAGGAGTGGT CTCCACAGTG CACATTTGCA CGCATGTTAG GACGAGGCTA TGGGGCACAG AGGGGCTATC

**orangutan**  TTGGAGGGGC TCAGGCTCCC AGGGTAGGGG CAGGAGTGGT CTCCACAGTG CACATTTGCA C--ACGTTAG GACGAGGCTA TGGGTCACAG AGGGGCCATT

**human**  TGCCCTGCCT GGAGACTGGT CTAGGGTTGC AGGGCCCACA TGTACTGCAT GCCCCCAAAA GGGTCAGGGG AAGGCTTCCT CCATCCCCTT GGGGCCACAG

**chimpanzee** TGCCCTGCCT GGAGACTGGT CTAGGGTTGC AGGGCCCACA TGCACTGCAT GCCCCCAAAA GGGTCAGGGG AAGGCTTCCT TCATCCCCTT GGGGCTGCAG

**orangutan**  TGCCCTGCCT GGAGACTGGT CTAGGGTTGC AGGGCCCACA TGCACTGCAT GCCCCCAAAA GGGTCAGGGG AAGGCTTTCT CCATCCCCTT GGGGCCGCAG

**human**  CCTCCTACTT GCCTAGGGAA ACATGGCTCT TGGAGGCCCA GGGAGGCCAC TACCCTGCTG AGCAGGCAGG CCCCAAACTA A

**chimpanzee** CCTCCTGCTT GCCTAGGGAA ACATGGCTCT TGGAGGCCCA GGGAGGCCGC TCCCCTGCTG AGCAGACAGG CCCCAAACTA A

**orangutan**  CCTCCCACTT GCCTAGGGAA ATATGGCTCT TGGAGGCCCA GGGAGGCCGC TCCCCTGCTG AGCAGGCAGG CCCCAAACTA A

ENSG00000176833

**human**  ATGCAAATGA CACAAAGATC CCATCACTTT CTAGTTTGGG GAGGGGGCG**G GCG**CAAGGGG AAGGAAGGAG GGAAGAAAGA CATCTTCCAA CTGTTATTAT

**chimpanzee** ATGCAAATGA CACAAAGATC CCATCACTTT CTAGTTTGGG GAGGGGGCG**- ---**CAAGGGG AAGGAAGGAG GGAAGAAAGA CATCTTCCAA CTGTTATTAT

**orangutan**  ATGCAAATGA CACAAAGATC CCATCACTTT CTAGTTTGGG GAGGGAGCG**- ---**CAAGGGG AAGGAAGGAG GGAAGAAAGA CATCTTCCAA CTGTTCTTAT

**human**  CTAGTCTCTG CAGGCTTAGA TTGGTGCTCT GCCTACATTG GAGGTGTTCT GCAAACCGGA GTCTTCCTCG CCAGAGTCCC ATAAACGTGC CTCAGTCAGA

**chimpanzee** CTAGTCTCTG CAGGCTTAGA TTGGTGCTCT GCCTACATTG GAGGTGTTCT GCAAACTGGA GTCTTCCTCG CCAGAGTCCC ATAAACGTGC CTCAGTCAGA

**orangutan**  CTAGTCTCTG CAGGCTTAGA TTGGTGCTCT GCCTACATTG GAGGTGTTCC GCAAACCGGA GTCTTCCTCA CCAGAGTCCC ATAAATGTGC CTCAGTCAGA

**human**  TGCTGGAGAG ACAGAGAAGG GAAGAGTCCA TCAGAGCCTC CTGATTCGAG CATCACACAG CAA-CCCTTC TGAGCTCGGT GGTCCACACA GCGCAGGGTT

**chimpanzee** TGCTGGAGAG ACAGAGAAGG GAAGAGTCCA TCAGAGCCTC CTGATTCGAG CATCACACAG CAA-CCCTTC TGAGCTCGGT GGTCCACACA GCGCAGGGTT

**orangutan**  CCCTGGAGAG ACAGAGAAGG GAAGAGTCCA TCAGAGCCTC CTGATTCGAG CATCACACAG CAAACCCTTC TGAACTCAGT GCTCCACACA GCGCAGAGTT

**human**  CTATGAACTC CTGAGGAGAG CCAGGAGTGC TGCTAAGCTT TCCCAGGTAT GCGTGACCAT GTGTGCCTTC ATCTGTGTGT GA

**chimpanzee** CTATGAACTC CTGAGGAGAG CCAGGAGTGC TGCTAAGCTT TCCCAGGTAT GCGTGACCAT GTGTGCCTTC ATCTGTGTGT GA

**orangutan**  CTATGAACTC CTGAGGAGAG CCAGGAGTGC TGCTAAGCTT TCCCAGGTGT GCGTGACCAT GTGTGCCTTC ATCTGTGTGT GA

ENSG00000204707

human ATGGGGAGGA GGGGTTTTGC AGAGCTGGGG AGAGCACAGA **--**CCACCGCG AGAGGCAGGT ACAATAGAAA CAGGTGTACT TTGCGCATCA CACAGAGGTG

chimpanzee ATGGGGAGGA GGGGTTTTGC AGAGCTGGGG AGAGCACAGA **GA**CCACCGCG AGAGGCAGGT ACAAGAGAAA CAGGTGTACT TTGCGCATCA CACAGAGGTG

orangutan ATGGGGAGGA GGGGTTTTGC AGAGCTGGGG AGAGCACAGA **GA**CCACCGCG AGAGGCAGGT ACAATAGAAA CAGGTGCACT TTGCCCGTCA CACAGAGGTG

human GGGACGACAG GCATGGGGTG GGGGCGGTGA GGCAGGAGCG CACCAGAGGA TATGTGGGAC CCGAAGGGGA GAGACGCATA AAAGCCGCAG CAGAGATGGG

chimpanzee GGGACGACAG GCATGGCGTG GGGGCAGTGA GGCAGGAGCG CACCAGAGGA TGTGTGGGAC CCGAAGGGGA GAGACGCATA AAAGCCGCAG CAGAGATGGG

orangutan GGGACGACAG GCATGGAGTG GGGGCAGCGA GGCAGGAGTG CACCAGAGGA TGTGTGGGAC CCAAAGGGGA GAGATGCATC AAAGCCGCAG CAGAGATGGG

human AGTACAGGGA GGGGAGGTCC CAATGCCCGC TCACCTCACC CCTTGGGGCC TGCCGGTGCC GGGGTCCCCA GGTCCCTGTC CCCCAGGCCT GTACCAGGGT

chimpanzee AGTACAGGGA GGGGAGGTCC CAATGCCCGC TCACCTCACC CCTTGGGGCC TGCCGGTGCC GGGGTCCCCA GGTCCCTGTC CCCCAGGCCT GTACCAGGGT

orangutan AGTACAGGGA GGGGAGGTCC CAGTGCCCGC TCACCTCACC CCTTGGGGCC TGCCGGTGCC GGGGTCCCCA GGTCCCTGTC CCCCAGGCCT GTACCAGGGT

human CTGAGCCCAG CACTGGTACA AGGGTTGGGA GACAGGGCCA TGGGGAGAGG AGCCGCCGCT GCCGCTGCTG CCTCGGGGAC GCTGCCTCCC CGTGGGCCCA

chimpanzee CTGAGCCCAG CACTGGTACA AGGGTTGGGA GACAGGGCCA TGGGGAGAGG AGCCGCCGCT GCCGCTGCTG CCTCGGGGAC GCTGCCTCCC CGTGGGCCCA

orangutan CTGAGCCCAG CACTGGTACA AGGGTTGGGA GACAGGGCCA TGGGGAGAGG AGCTGCCGCT GCCGCTGCTG CCTCGGGGAT GCCGCCTCCC CTTGGGCCCA

human GTCCCTGCCT TATACCCAGG TCCAGGGAGG GCGGGGGTTC CTGCCAGAGG AAGTGGGGCC GCATCAAAGA GGGCAGGAAG GGGCAGGGAC AGGTTGGGGG

chimpanzee GTCCCTGCCT TATACCCAGG TCCAGGGAGG GCGGGGGTTC CTGCCAGAGG AAGTGGGGCC GCATCAAAGA GGGCAGGAAG GGGCAGGGAC AGGTTGGGGG

orangutan GTCCCTGCCT TATACCCAGG TCCAGGGAGG GCGGGGGTTC CTGTCAGAGG AAGTGGGGCC GCATCAAAGA GGGCAGGAAG GGGCAGGGAC AGGTTGGGGG

human GACGCCACCC TGAGTCGCAG AAACTGCACA CCCACTCCAC CCTATGCTGG GGGCAGTAGA GAACCAGCAA GGGGCAAAGA GGGAGTGTGG ACTCCAGAGG

chimpanzee GACGCCGCCC TGAGTCGCAG AAACTGCACA CCCACTCCAC CCTATGCTGG GGGCAGTAGA GAACCAGCAA GGGGCAAAGA GGGAGTGTGG ACTCCAGAGG

orangutan GACGCCGCCC TGAGTCGCAG AAACTGCACA CCCACTCCAC CCTATGCTGG GGGCAGTAGA GAACCAGCGA GGGGCAAAGA GGGAGTGTGG ACTCCAGAGG

human GAGGGGACGG AGACCCAGAG GGGAATAGGA GGTTGCGGCC CCCTCCCCTC AGCCGCAGGT GTTTCCGTTC GGGTGGGGCT GCTGTGTTTA CAACAACAGC

chimpanzee GAGGGGACGG AGACCCAGAG GGGAATAGGA GGTTGCGGCC CCCTCCCCTC AGCCGCAGGT GTTTCCGTTC GGGTGGGGCT GCTGTGTTTA CAACAACAGC

orangutan GAGGGGACGG AGACCCAGAG GGGGATGGGA GGTTGCGGCC CCCTCCCCTC GGCCGCAGGT GTTTCCGTTC GGGTGGGGCT GCTGTGTTTA CAACAACAGC

human CCGGCTCCCC GGTTTCCTCC CTGATTGTTG GGGCGTCTCC TTGGCACCCC ATCACGTCAG GTGGTGTGCT GAGGTCAGAG TGTGTGCTGG GGAAGGACCT

chimpanzee CCGGCTCCCC GGTTTCCTCC CCGATTGTTG GGGCGTCTCC TTGGCACCCC ATCACGTCAG GTGGTGTGCT GAGGTCAGAG TGTGTGCTGG GGAAGGACCT

orangutan CCGGCTCCCC GGTTTCCTTC CCGATTGTTG GGGCGTCTCC TTGGCACCCC ATCACGTCAG GTGGTGTGCT GAGGTCAGAG TGTGTGCTGG GGAAGGACCA

human CTGCTGGGCC TGCTGGATTG CAAAGAGCAA GTTTAA

chimpanzee CTGCTGGGCC TGCCAGATCG CAAAGAGCAA GTTTAA

orangutan CTGCTGGGCC TGCTGGATCG CAAAGACCAA GTTTAA

ENSG00000212693

human **ATG**TATGACC ATGGGTATGA T---ACCTCC AGAAATGTTA ACAACTTATT CTTCTGCCAT GAGTACCCCT CATCAGGGTT GGTTTCAATG ACAGGTTTTG

chimpanzee **GTG**TATGACC ATGGGTATGA TGATACCTCC AGAAATGTTA ACAACTTATT CTTCTGCCAT GAGTACCCCT CGTCAGGGTT GGTTTCAATG ACAGGTTTTG

orangutan  **GTG**TATGACC ATGAGTATGA T---ACCTCC AGAAATGATA ACAACTTATT CTTCTGCTAT GAGCACCCCT CATCAGGGTT GGTTTCAATG ACAGGTTTTG

human GTGATGTTCC TGACCATATG AAGTGGTTTA TGTTTAGAAA CATTCAAATT GAGGGACATC ATTTACAGCA TCGAGTGTGT CAGTTATACA TGCATTCATC

chimpanzee GTGATGTTCC TGACCATATG AAGTGGTTTA TGTTTAGAAA CATTCAAATT GAGGGACATC ATTTACAGCA TCGAGTGTGT CAGTTATACA TGCATTCATC

orangutan GTGATGTTCC TGACCGTATG AAGTGGTTTA TGTTTAGAAA CATTCAAATT GAGGGACATC ATTTACAGCA TCGAGTGTGT CAGTTATACA TGCATTCATC

human ATTAAATATC CTAGGTTTTA TGACAGACAT TGAAATTATC ACTCAGATGT GTTTAACAGC AAAAAATTCC CAGCACATCC TGGCAAAGGC TTTTATTTCC

chimpanzee ATTAAATATC CTAGGTTTTA TGACAGACAT TGAAATTATC ACTCCGATGT GTTTAACAGC AAAAAATTCC CAGCACATCC TGGCAAAGGC TTTTATTTCC

orangutan ATTAAATATC CTGGGTTTTA TGACAGACAT TGAAATTATC ACTCAGATGT GTTTAACAGC AAAAA-TTCC CAGCACATCC TGGCAAAAGC TTGTGTTTCC

human AACCGTTGCA TTCTTCATCT CTGCCTCCCA TTGCCCACTG AATGCTTTGC TTTCTGTGCA TCAAGACAGA GTTCTAAAAC CAGAAAACAT CCATCTTGA

chimpanzee AACCGTTGCA TTCTTCATCT CTGCCTCCCA TTGCCCACTG AATGCTTTGC TTTCTGTGCA TCAAGACAGA GTTCTAAAGC CAGAAAACAT CCATCTTGA

orangutan AACCGTTGGG TTCTTCATCT CTGCCTCCCA TTGCCCACTG AATGCTTTGC TTTCTGTGCA TCAAGACAGA GTTCTAAAAC CAGAAAACAT CCATCTTGA

ENSG00000197916

human ATGCAGCCCT GTACTCCTGG CCTCAAGCCA TCCTCCCACC TCAGCCTCCA GAGTAGCTGG GAGCACAAGT GTGCACCATC ACACCCAGCT AACGTTTATT

chimpanzee ATGCAGCCCT GAACTCCTGG CCTCAAGCCA TCCTCCCACC TCAGCCTCCA GAGTAGCTGG GAACACAGGT GTGCACCATC ACACCCAGCT AACGTTTATT

orangutan ATGCAGTCCT GACCTCCTGG CCTGAAGCCA TCCTCCCACC TCAGCCTCCA GAGTAGCTGG GAGCACAAGT GTGCACCATC ACACCCAGCT AACGTTTATT

human TTTTTGTAGA GAGAGTCTCA CT**CACT**ATGT TGCCCAGGCT GGTCTTAAAA CTCCTGGCCT CAACTGATCC TCCTC--CTT TGGCCTCCCA AAGTGCTGGG

chimpanzee TTTTTGTAGA GAGAGTCTCA CT**----**ATGT TGCCCAGGCT GGTCTTAAAA CTCCTGGCCT CAACTGATCC TCCTCTCCTT TGGCCTCCCA AAGTGCTGGG

orangutan TTTTTGTAGA GAGAGTCTCA CT**----**ATGT TGCCCAGGCT GGTCTTAAA- CTCCTGGCCT CAACTGATCC TCCTC--CTT TGGCCTCCCA AAGTGCTGGG

human ACCATAGGCA TGAGCTGCCA TTGCCAGCTA ATTGTTTTAT TTTTACATTT GCTGAGGCAG GGTCTCGCT- TGTGGCC-GT GCAGATCTTG AACTCCTGGC

chimpanzee ACCATAGGCA TGAGCTGCCA TTGCCAGCTA ATTGTTTTAT TTTTACATTT GCTGAGGCAG GGTCTCGCT- TGTTGCC-GT GCAGATCTTG AACTCCTGGC

orangutan ACCATAGGCA TGAGCTGCCA TTGCCAGCTA ATTTTTTAAT TTTTACATTT GCTGAGGCAG GGTCTCACTG TGTTGCCCAG GCAGATCTTG AACTCCTGGC

human CTTGAGCAGT ACCTCCCTCC TGGGTCTCTT ACAGTGCTGG GATGATAGGG GTGAGCCGCT GTGCCCGGCC CGGGGTTTCT TTTTGAAGTG GTGA

chimpanzee CTTGAGCAGT ACCTCCCTCC TGGGTCTCTT ACAGTGCTGG GATGACAGGC GTGAGCCGCT GTGCCCGGCC CGGGGTTTCT TTTTGAAGTG GTG-

orangutan CTTGAGCAGT ACCTCCCTCC TCGATCTCTT AAAGTGCTGG GATGACAGGT GTGAGCCGCT ATGCCCGGCC CAGGGTTTCT TTTTGAAGTG GTGA

ENSG00000205373

human ATGACCGCAA CCCTCGGGTC CCGAGGCGCC GCTCCAGGCG GGAGCGCCAA CTCCCACGGC CCTTCCGACC CCCGCGTCCC ATTGTTCCGA ATCTCACTCA

chimpanzee ATGACCGCAA CCCTCGGGTC CCGAGGCGCC GCTCCAGGCG GGAGCGCCAA CTCGCACGGC CCTTCCGACC CCCGCGTCCC ATTGTTCCGA ATCTCACTCA

orangutan ATGACCGCAG CACTCGGGTC CCGAGGCGCC GCTCCAGGCG GGAGCGCCAG CTCGCACGGC CCTTCCGACC CCCGCGTCCC ATTGTTCCGA ATCTCACTCA

human CCTCGGAGGA TGCCGGCGCA GGCCTGGCTG GGGCGCTCGC CGCGAGCTCC CGGCAGCCCA GAAAGCTAGG TCGG**TGT**CGG CAGGATCCGC GAAGGCGTAC

chimpanzee CCTCGGAGGA TGCCGGCGCA GGCCTGGCTG GGGCGCTCGC CGCGAGCTCC CGGCAGCCCA GAAAGCTAGG TCGG**TGA**CGG CAGGATCCGC GAAGGCGTAC

orangutan CCTCGGAGGA TGCCGGCGCA GGCCTGGCTG GGGCGCTCGC CGCGAGCTCC CGGCAGCCCA GAAAGCTAGG TCGG**TGA**CGG CAGGATCCGC GAAGGCGTAC

human CCGGCGACTT CTCCGCCTAC CCTCCGAAGC GGGAGCAGGC GGGGGCGCTT CACCCTCCTC GCGAGACAGC GGAGACCCCA GCGGCGGCTG CGACCCCTGT

chimpanzee CCGGCGACTT CTCCGCCTAC CCTCCGAAGC GGGAGCAGGC GGGGGCGCTT CACCCTCCTC GCGAGACAGC GGAGACCCCA GCGGCGGCTG CGACCCCTGT

orangutan CCGGCGACTT CTCCGCCTAC CCTCCGAAGC GGGAGCAGGC GGGGGCGCTT CACCCTCCTC GCGAGACAGC GGAGACCCCA GCGGCGGCTG CGACCCCTGT

human GACGCAAAGG GCTCATCACC AATGGCAGCT CGGAAGCAGG GCAGAGAGCG AGACCCACCC CCCAGCTCCC G-CGCATCAA AACACAACAC CCGCCTTCCT

chimpanzee GACGCAAAGG GCTCATCACC AATGGCAGCT CGGAAGCAGG GCAGAGAGCG AGACCCACCC CCCAGCTCCC G-CGCATCAA AACACAACAC CCGCCTTCAC

orangutan GACGCAAAGC GCTCATCACC AATGGCAGCT CGGAAGCAGG GCAGAGAGCG AGACCCACCC CCCAGCTCCC GGCGCGTCAA AACACAACAC CCGCCTTTGC

human CACGCATGCG CAGCAGATGG ACGCCGGCCT CGGGGGCGGG GCTCAGCGCA CCGGCCCAAA TGCGGCCCCT CCCCCGCCGC TCCAGACCCC GGAGGAGGGC

chimpanzee CACGCATGCG CAGCAGATGG ACGCCGGCCT CGGGGGCGGG GCTCAGCGCA CCGGCCCAAA TGCGGCCCCT CCCCCGCCGC TCCAGACCCG GGAGGAGGGC

orangutan CACGCATGCG CAGCAGATGG ACGCCGGCCC CGAGGGCGGG GCTCAGCGCA CCAGCCGAAA CGCGGCCCCT CCCCCGCCGC TCCAGACCCG GGAGGAGGGC

human AGCGCGACCC TCCCCATCTT GTTACTGGTA CTCAGCCACT GGGGACACT- GACTGAATGT TCGCCCCTAA CTTACCCGGA AAACACGAGA ACTAGTTCGA

chimpanzee AGCGCGACCC TCCCCATCTT GTTACTGGTA CTCAGCCACT GGGGACACT- GACTGAATGT TCGCCCCTAA CTTACCCGGA AAACACGAGA ACTAGTTCGA

orangutan AGGGAGACCC TCCCTATCTT GTTACTGGTA CTCAGCCACT GGGGACACTT GACTGAATGT TCGCCCCTAA CTTACCCGGA AAACACGAGA ACTAGTTCGA

human TGTCGTTTCA ACCTTACCAA ACTCTGCGGA AACTTAGGAA ATTATCTTTA GTGTCTAGAT AG

chimpanzee TGTCGTTTCA ACCTTACCAA ACTCTGCGGA AACTTAGGAA ATTATCTTTA GTGTCTAGAT AG

orangutan TGTCGTTTCA ACCTCACCAA ACTCTGCGGA AACTTAGGAA ATTGTCTCTA GTGTCTAGAT A-
