# Supplementary material for: De Novo Origin of Human Protein-Coding Genes
Source: PLoS Genet. 2011 Nov 10;7(11):e1002379. doi: 10.1371/journal.pgen.1002379 (PMC3213175; doi:10.1371/journal.pgen.1002379)
Supplement: Dataset S5 — Protein evidence for the 33 de novo genes in Table S2. (DOC) [file pgen.1002379.s012.doc]

ENSG00000187488

489: AQEVPALGTASSVAPAR

8543: KAPRSWDSATPSEMLCPF

ENSG00000183853

350 FNELHAYQPQAITTLMDGSGGRHSWPTPCR

8412 KMFLALSPWSPFIIHVRF

8538 KMFLALSPWSPFIIHVRF

ENSG00000203862

8659 CHLLNEVIMIFFLK

ENSG00000198447

453 MKSISFSLGSGGEAAPGR

ENSG00000205965

8660PLSLWVGLGNWRGGR

8665PLSLWVGLGNWRGGR

ENSG00000184827

297KPPPPHPSSMPGHSWGQSDVAGVGMPK

375EVGAGSDCSDVQSLWDASSALSSLPLPLGR

1622TTRICHLLAKR

8322WGQSDVAGVGMPKMK

8669PPPPHPSSMPGHSWGQSDVAGVGMPKMK

8672RGLGLQVSALASVGPHSFASFAPCLSFL

15390LGRMTQSGRR

ENSG00000188745

8670 PRASVSR; TGPLQCGTVGDAEHNTGWWIPGR

ENSG00000206028

8666 AIPCLRPGPVDSDLTANMGQQSGTSVYR

8670 DVILRVQPPTSTGPPLPPLSLVNR

8671 PTGLHTHVPEISLVPPTPSGNQTSAHRR; VQPPTSTGPPLPPLSLVNR

ENSG00000204626

8670 NPHSWGIKAHGLR; LERCMVPESEWAPWQPQLPCEPK

ENSG00000198411

239 YILGSRIAGSYGSSIVR

366 YILGSRIAGSYGSSIVR

ENSG00000218478

8667 MYFCGLGVRWGWGR

8664 MYFCGLGVRWGWGR

8667 VLSTDPAAHPCAVR; MYFCGLGVRWGWGR

ENSG00000204380

8538 RMVPPRPDSLTGLRSSYAS

8658 DLGVSLFPPFPRS;

8670 DLGVSLFPPFPRS; SAPGPVLSSSLHSPRCLLLLVFNTTYGHR

ENSG00000167117

8669 VWLGACCASLASPPKGTIPSGEYYR

8670 MTLLYLTGLARTHCCYLADR; THCCYLADRCAEAVESAFYLVGSLCINAR; VWLGACCASLASPPKGTIPSGEYYR

9297 GAAHLTD

9301 PAPSSSGDSLR

ENSG00000162968

8667 VFHPKGLCCGR; GLCCGRCSDPR

ENSG00000204292

8668 MMLSIRVPGQPAALR; RMMLSIR

ENSG00000205557

8663 SLVTLSAHQGR

ENSG00000204506

8660 WQHLFRIFLIR

8665 QEVSGGYVGCRCPSSCSLGAIPG

8667 GMWRQEVSGGYVGCR

8670 QEVSGGYVGCRCPSSCSLGAIPG

ENSG00000204079

8668 HCMASGAVLNK

8669 MISVHCNLCLPGSSDPPASASQVAGITGVR

ENSG00000205201

8653 TSPYIFLPHHESIYQQAYR

8657 AAPEEVAGCGILRSLHSSK

ENSG00000204412

8531 HHQRGTSLSWGSHVELYLE

8653 SHGGVGIKEK

ENSG00000176911

203 RNAEWHVHMMEYYAAENMNNWSHGMNEAER

ENSG00000180838

156 CSPGLCRSSTAGQCLWGGVGGASEAAP

ENSG00000206110

1904 GQKSLIQSQGQALRLK

8659 KSQMLEDASSMLQSSEGK

8668 KSQMLEDASSMLQSSEGK

8669 KSQMLEDASSMLQSSEGK

ENSG00000214467

8531 LFLRWSLILSPKLEDSSRISAHCNLS

8653 STVSLSIGDICKCLR

8665 STVSLSIGDICKCLR

8672 STVSLSIGDICKCLR; CLRGVFVFCLFVCLFVCLFLR

ENSG00000136242

8476 GQGRGRTAGARARGAPWES

8653 KMFMAAAAVAVGAAR

ENSG00000206096

8667 TVFPLPQVGHR; QNPSEEMVASVQDTNQHGNLHYRMR

8668 QNPSEEMVASVQDTNQHGNLHYRMR

ENSG00000204581

8668 EGNMWVRVAAMGVNMIK

8670 CISELTVMTQIGREGNMWVR; CISELTVMTQIGREGNMWVR

ENSG00000205424

1915 VAGPTCTACPQK

8653 ASAVGGAQAPR

10885 SSHGGAQRSGLR

ENSG00000176833

673 QSPINVPQSDAGETEKGR

1624 SAAKLSQVCVTMCAFICV

8661 SAAKLSQVCVTMCAFICV

8664 SAAKLSQVCVTMCAFICV

8669 LSQVCVTMCAFICV

8671 SAAKLSQVCVTMCAFICV

ENSG00000204707

8670 QAWGGGGEAGAHQRICGTR; WGRQAWGGGGEAGAHQR

ENSG00000212693

8234 YPSSGLVSMTGFGDVPDHMKWFMFR

8669 CILHLCLPLPTECFAFCASR

8670 VCQLYMHSSLNILGFMTDIEIITQMCLTAK

ENSG00000197916

8666 QGLACGR

ENSG00000205373

8543 SLTSEDAGAGLAGALAASSRQPRKLGRCRQD

8662 VPLFRISLTSEDAGAGLAGALAASSR; MTATLGSRGAAPGGSANSHGPSDPR

8670 VPLFRISLTSEDAGAGLAGALAASSR; MTATLGSRGAAPGGSANSHGPSDPR

8672 LPSEAGAGGGASPSSR
